# Utility of EST-SNP Markers for Improving Management and Use of Olive Genetic Resources: A Case Study at the Worldwide Olive Germplasm Bank of Córdoba

**DOI:** 10.3390/plants11070921

**Published:** 2022-03-29

**Authors:** Angjelina Belaj, Antònia Ninot, Francisco J. Gómez-Gálvez, Milad El Riachy, Melek Gurbuz-Veral, Mariela Torres, Adhurim Lazaj, Tatjana Klepo, Sergio Paz, Javier Ugarte, Luciana Baldoni, Ignacio J. Lorite, Zlatko Šatović, Raúl de la Rosa

**Affiliations:** 1 Centro “Alameda del Obispo”, Instituto Andaluz de Investigación y Formación Agraria, Pesquera, Alimentaria y de la Producción Ecológica, IFAPA, 14004 Cordoba, Spain; franciscoj.gomez.galvez@juntadeandalucia.es (F.J.G.-G.); ignacioj.lorite@juntadeandalucia.es (I.J.L.); raul.rosa@juntadeandalucia.es (R.d.l.R.); 2 IRTA, Mas Bové, Constantí, 43120 Tarragona, Spain; antonia.ninot@irta.cat; 3 Department of Olive and Olive Oil, LARI, Bekaa, Zahle P.O. Box 287, Lebanon; mraichy@lari.gov.lb; 4 Department of Breeding and Genetics, Olive Research Institute, İzmir 35100, Turkey; melekgurbuz11@gmail.com; 5 Estación Experimental Agropecuaria (SJ) and CONICET, INTA, San Juan 5400, Argentina; torres.mariela@inta.gob.ar; 6 Qendra e Transferimit te Teknologjise Bujqesore, QTTB, 9400 Vlorë, Albania; adhurimlazaj@gmail.com; 7 Center of Pomology, Croatian Agency for Agriculture and Food, 21210 Solin, Croatia; tatjana.klepo@hapih.hr; 8 Servicio de Transferencia de Tecnología Conselleria de Agricultura, Desarrollo Rural, Emergencia Climática y Transición Ecológica—Generalitat Valenciana, 46113 Valencia, Spain; paz_ser@gva.es; 9 Servicio de Investigación Agraria y Sanidad Vegetal, Gobierno de La Rioja, 26071 Logroño, Spain; jugarte@larioja.org; 10 Institute of Biosciences and Bioresources, CNR, 06100 Perugia, Italy; luciana.baldoni@ibbr.cnr; 11 Department of Seed Science and Technology, Faculty of Agriculture, University of Zagreb, 10000 Zagreb, Croatia; zsatovic@agr.hr; 12 Centre of Excellence for Biodiversity and Molecular Plant Breeding (CoE CroP-BioDiv), 10000 Zagreb, Croatia

**Keywords:** olive germplasm, EST-SNPs, genotyping, synonyms, homonyms, genetic diversity

## Abstract

Olive, the emblematic Mediterranean fruit crop, owns a great varietal diversity, which is maintained in ex situ field collections, such as the World Olive Germplasm Bank of Córdoba (WOGBC), Spain. Accurate identification of WOGBC, one of the world’s largest collections, is essential for efficient management and use of olive germplasm. The present study is the first report of the use of a core set of 96 EST-SNP markers for the fingerprinting of 1273 accessions from 29 countries, including both field and new acquired accessions. The EST-SNP fingerprinting made possible the accurate identification of 668 different genotypes, including 148 detected among the new acquired accessions. Despite the overall high genetic diversity found at WOGBC, the EST-SNPs also revealed the presence of remarkable redundant germplasm mostly represented by synonymy cases within and between countries. This finding, together with the presence of homonymy cases, may reflect a continuous interchange of olive cultivars, as well as a common and general approach for their naming. The structure analysis revealed a certain geographic clustering of the analysed germplasm. The EST-SNP panel under study provides a powerful and accurate genotyping tool, allowing for the foundation of a common strategy for efficient safeguarding and management of olive genetic resources.

## 1. Introduction

In olive (*Olea europaea* subsp. *europaea*) tree crop species many efforts have been devoted to the collection and conservation of genetic resources. This has led to the establishment of over 100 ex situ field collections in Mediterranean countries and beyond [1,2]. These collections represent essential tools for the acquisition, maintenance, documentation, assessment, and use of the genetic diversity of the crop, which is estimated to include around 1200 clonally propagated cultivars with more than 3000 different names [1,2,3,4]. In this sense, the International Olive Council (IOC) launched in 1994 a network of National Germplasm Banks in olive growing countries. This network, which currently includes 23 germplasm collections, made possible sampling and cataloguing of around 1700 accessions by means of a common method of morphological characterisation [5,6]. Three world olive germplasm banks have been acknowledged and/or created in Córdoba (Spain), Marrakech (Morocco), and Izmir (Turkey) within this network [2].

The awareness in the 1970s of the importance of conserving olive germplasm, prior to suffering genetic erosion or loss, led to the creation of the first World Olive Germplasm Bank of Córdoba (WOGBC). This international collection was established at the experimental field “Alameda del Obispo” of the Andalusian Institute for Research and Training in Agriculture, Fishery, Food and Organic Production (IFAPA) through a joint project between Food and Agriculture Organization (FAO) and National Institute for Agriculture and Food Research (INIA) with the IOC support [2,7,8]. It represents the reference olive germplasm bank (ESP046) commissioned for the safeguard of national olive genetic resources, belonging to the Spanish Genebanks Network co-ordinated by INIA [7,9]. From its foundation, WOGBC has been continuously enriched with new accessions from national and international prospecting surveys, as well as accessions provided by partners of the IOC network and/or different scientific institutions [2,8,10]. Nowadays, WOGBC accounts for more than 1000 accessions from 29 countries, around 33% of them being of national (Spanish) origin. The plant material maintained at the WOGBC collection and its study have contributed to the generation of important knowledge of species diversity at the morphological, agronomical [5,11], molecular [12,13], and genomic level [14], as well as to make it available for comparative trials and olive breeding programmes [15].

Management and evaluation of olive genetic resources in a germplasm collection is a complex multi-disciplinary, costly, and everlasting task. Therefore, efficient strategies to maximise the value of this infrastructure and of olive germplasm resources are needed. Accurate genotype identification is crucial and represents the first step toward a correct management of olive germplasm [3,6,16]. In this sense, different morphological and molecular markers [5,16,17], especially SSRs, have been developed and applied at WOGBC germplasm collection for olive cultivars’ identification and genetic diversity studies [6,18]. Although SSR markers have contributed significantly to improving management and knowledge of olive diversity maintained in germplasm collections [3,6], their genotyping presents some main drawbacks and limitations [19,20]. For instance, establishing a clear cut-off between intra versus inter-cultivar variability is not easy and it may lead to difficulties for cultivar discrimination [3,6,8]. In addition, allele size discrepancies need to be adjusted and harmonised for comparisons among different collections and within a large SSR dataset [3,19,20].

Recently, the application of SNP markers for olive germplasm management has revealed that they may have clear advantages over previously used molecular markers in terms of their efficiency. Thus, olive fingerprinting by SNP markers can be fully automated in high-throughput assays, i.e., cost-effective, they display low genotyping error rates and may become very useful to compare data across different laboratories, germplasm collections, and genotyping platforms. These advantages have resulted in increasing efforts for development and use of SNP markers as the markers of choice for identification and diversity studies in the last years [21,22,23,24,25,26]. Meanwhile, their low levels of diversity may be overcome by selecting an optimal number of markers [22].

The present research is part of an ongoing project aimed at improving the management and use of the genetic resources maintained at WOGBC by means of reliable, practical, and cost-effective fingerprinting techniques. The first stage of this project consisted of using EST sequences [27] as a means of developing a set of SNP markers [24]. In this sense, the 1043 new EST-SNPs were able to reliably discriminate among different accessions to reveal a clear cut-off between inter- and intra-cultivar variation in olive, as well as to efficiently detect possible homonymy cases and the presence of redundant germplasm in the collection. The high number of markers developed and their efficiency allowed the selection of an optimum core set of 96 EST-SNP markers. The present study is the first report of the use of this set of 96 markers for the fingerprinting of the plant material maintained at the WOGBC collection. Including a total of 1273 accessions from 29 countries, this research is, to the best of our knowledge, the largest one performed to date in olive. In the present study, the set of the selected 96 EST-SNP markers was used in order to: (a) reliably identify the accessions maintained in the field and at different propagation facilities of the WOGBC, (b) use the information for duplication assessment and management strategies to reduce them as much as possible, as well as to devise sampling strategies for future collection of olive germplasm, (c) to propose a common identification protocol by means of 96 EST-SNPs that can be used by regional, national, and international olive germplasm collections, as well as (d) to study the genetic structure and the relationships among the different olive cultivars identified in the present research.

## 2. Results

### 2.1. Genotyping of WOGBC by Means of EST-SNPs

The genotyping by means of 96 EST-SNPs showed total concordance between independent DNA extractions from the same trees of the two reference cultivars (“Picual” and “Frantoio”), as well as different trees and accessions of the same cultivar, demonstrating their accuracy for generating olive DNA fingerprints. The histogram constructed on distances proportional to the number of different alleles for all allele comparisons showed an exceptionally low genotyping error rate and also a low possible intra-cultivar variation (Figure S1). In this sense, a very clear separation was observed between the possible intra-cultivar variability (ranging from zero to four different alleles) and the inter-cultivar variability (ranging from 19 to 86 alleles).

The EST-SNPs were first used to identify the olive cultivars maintained in the field collection. The information obtained was then used for genotyping new accessions maintained at different propagation facilities prior to their planting in the field (Table S1).

Overall, the EST-SNP genotyping of the 1273 olive accessions (3105 trees/plants) made possible the identification of 668 different cultivars. Most of them (520 cultivars) were already planted in the field, while the remaining 148 were identified among the new accessions. In total, 45 out of these 148 cultivars belonged to the new plant material coming from regional germplasm collections and local Spanish prospecting surveys.

### 2.2. Evaluation of WOGBC Redundancies

In spite of the high number of cultivars identified, the pairwise comparison of the accessions also revealed a considerable level of redundant germplasm. Thus, 605 accessions shared the same EST-SNP genetic profile, with at least another accession from WOGBC. Among the redundant accessions, 489 were field accessions, representing 48.5% of the total accessions maintained in the field. The remaining 116 redundancies were detected among the new accessions (43.6% of the total). The redundant accessions clustered in 204 different genotypes (out of the 668 identified), with redundancy sizes ranging from 2 to 39 accessions. The largest group of redundant accessions was that of the Lebanese cultivar Baladi that included 39 identical accessions, followed by the groups of cultivars Frantoio and Safrawi composed of 27 and 19 redundant accessions, respectively.

The redundancies detected among WOGBC accessions could mostly be classified in three main cases: (i) accessions/cultivars with different names but identical fingerprints (synonymy cases, prospecting redundancies), (ii) accessions/cultivars with identical and/or very similar names distinguished by different register numbers but sharing the same EST-SNP fingerprints, and (iii) mislabelled accessions or mistakes at different stages of their inclusion into the collection. Most of the redundancies (63.04% of the total ones) detected in the present study fall into the first case, followed by redundant accessions (23.49% of the total) included in the second case, while 7.80% of the total redundant accessions belonged to the third case. In addition, a much lower percentage (5.67%) includes uncatalogued and/or unsolved redundancy cases.

A total of 510 accessions belonging to 140 different cultivars were identified as possible synonyms. Out of the 510 accessions, 183 were identified as newly observed synonyms (Table S3). Besides, 215 of those 510 accessions shared the same genotype with at least one accession from the same country, while the rest (295) included synonymy cases at both within and between olive growing countries. This is the reason why the total number of different cultivars/genotypes found in the present study was 668 but, if we sum the different cultivars per country, we have a total of 764 (Table 1).

**Figure 1:**
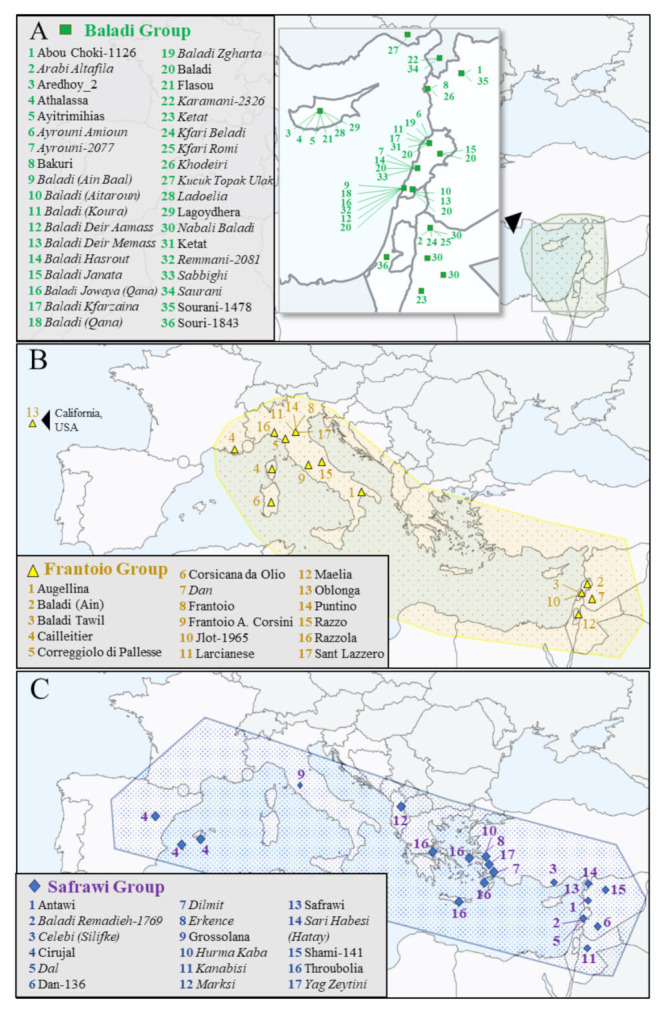

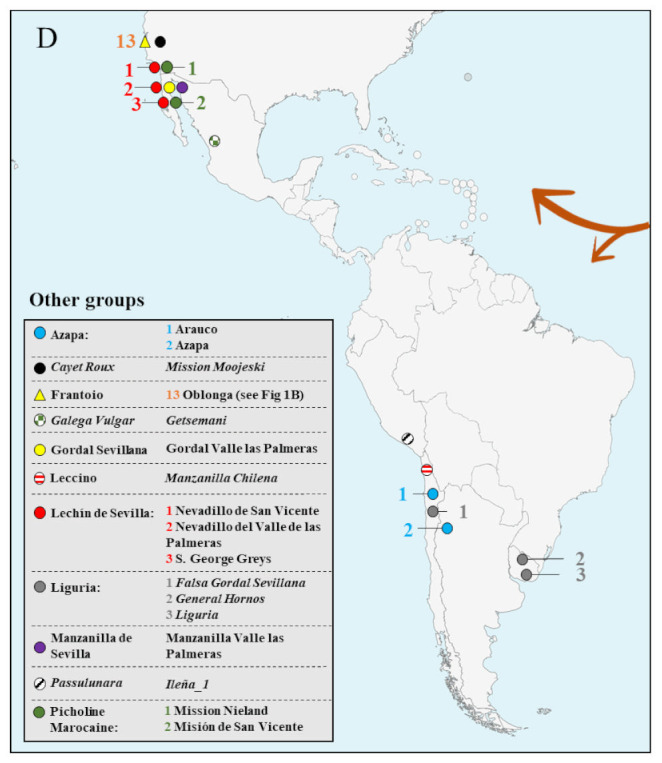
Geographical areas of distribution of the synonymy cases included in the biggest redundancy groups of “Baladi” (**A**, green squares), “Frantoio” (**B**, yellow triangles), and “Safrawi” (**C**, blue diamonds) in the Mediterranean basin (**A**–**C**), as well as synonyms found in America (**D**, circles in different colours). Accessions within each group are presented alphabetically and numbered accordingly. Italics indicate new observed synonymy cases.

In this regard, the redundancy groups of cultivars Baladi, Frantoio, and Safrawi represent good examples of the spreading of the same cultivars in olive growing regions of the same country and/or in different countries but under different names, i.e., synonymy cases (Figure 1A–C; Table S3).

In the case of “Baladi”, the fact that this name means “local” or “from the country” in Arabic could explain the large number of synonymies (17) found within Lebanon (Figure 1A, Table S3). Most of these synonymies (11 out of 17) were named after the generic name “Baladi” followed by the name of the localities of their cultivation in Lebanon (Aitaroun, Qana, Koura, Ain Baal, Deir Aamass, Deir Memass, Hasrout, Janata, Kfarzaina, Jowaya, and Zgharta). On the other hand, 19 accessions from neighbouring and nearby countries were also included in this redundancy group. Thus, five cultivars from different olive growing areas of Jordan, one from Israel, four from west and northwest Syria, three from southeast Turkey, as well as six from Cyprus shared the same genotype with “Baladi”. Among the 39 redundant accessions, 23 were identified in the present work for the first time. Besides, 24 redundant accessions were identified among the new accessions and prior to their introduction into the field collection. Finally, in addition to the 36 synonymy cases (Figure 1A), the three remaining redundant accessions included one accession with an identical name but different register number, as well as two possible mistakes at different stages of their inclusion into the collection.

The redundancy group of the main Italian cultivar Frantoio (its name means olive mill), comprised nine synonymy cases from almost all olive growing areas of the country (“Augellina”, “Frantoio A. Corsini”, “Correggiolo di Pallese”, “Larcianese”, “Razzo”, “Razzola”, “Puntino”, and “San Lazzero”), including the Island of Sardinia (“Corsicana da Olio”). Besides, “Frantoio” not only shared the same EST-SNP genotype with cultivars from neighbouring countries, such as France (“Calletier”), but from distant olive growing countries as well, including cultivars from Lebanon (“Baladi Ain”, “Baladi Tawil”, “Jlot-1965”), Syria (“Dan”), Israel (“Maelia”), and USA (“Oblonga”) (Figure 1B).

The third largest group of redundancy was that of the Syrian cultivar “Safrawi”. Meaning “yellow colour” in Arabic, probably referring to the colour of its fruits, this cultivar is received/collected with different names (“Dan-136”, “Antawi”, and “Shami-141”) from Syrian olive growing areas, thus representing synonymy cases within the country. At the same time, many synonymy cases of this cultivar were detected in southern and northern neighbouring countries, including two cultivars from Lebanon, one from Jordan, and six from southeast and Mediterranean Turkey. In addition, cultivars from Greece (“Throubolia”), Albania (“Marksi”), Italy (“Grossolana”), and Spain (“Cirujal”) also shared the same EST-SNP genotype with “Safrawi” (Figure 1C). It is worth mentioning that the cultivar “Safrawi” did match the endocarp profiles of voucher stones received from prospecting trials in Syria (Caballero and del Río, unpublished data), as well as DNA samples from neighbouring countries, including centennial olive trees (Ninot A., unpublished data). For this reason, it was considered appropriate to name the group as “Safrawi” instead of “Cirujal” cultivar as it was previously reported [3,6].

As expected, most of the synonyms detected included accessions/cultivars from traditional olive growing areas (Table S3). However, in new growing areas, such as those of the American continent, the introduced cultivars also acquired new names. For instance, the redundancy group of “Picholine Marocaine” that encompassed 17 redundant genotypes also included two North American cultivars Misión de San Vicente from Mexico and Mission Nieland from the USA. Similarly, the Spanish cultivar Lechín de Sevilla was renamed as “Nevadillo Valle las Palmeras” and “Nevadillo de San Vicente” in Mexico, while, in USA, as “S. George Greys” (Figure 1D; Table S3). It is interesting to mention that exclusive synonymy cases have also been observed in South America, such as the redundancy group of the cultivar Azapa including three accessions from Chile and two from Argentina. Besides, new synonymy cases including the accession “Liguria” from Chile and the pair of accessions “Falsa Gordal Sevillana” and “General Hornos”, from Uruguay, were also detected (Figure 1D; Table S3). In addition, synonymy cases were also detected among accessions collected in prospected surveys in Spain (“Olivo de Nueva Carteya”—“Hendero”), Albania (“Marksi”—“Safrawi”) and Bosnia and Herzegovina (“Studenci”— “Ljubuski Stari Grad”—“Oblica”).

Despite the general tendency of renaming the introduced cultivars, in some cases, the synonyms are due to almost literal translation of cultivar´s names from one language to the other. This is the case of the pairs of accessions “Sari Habesi (Hatay)”—“Safrawi” (yellow in Turkish and Arabic), “Esek zeytini (Odemis)”—“Gaydoyrelia” (donkey olive, i.e., big fruit, both in Turkish and Greek), while two independent cases of synonymies “Ulliri i Bardhe i Tiranes”—“Bjelica” and “Bianchera”—“Istarska Bjelica”—“Istrska Belica” were found to include the same meaning “white colour” in Albanian, Croatian, Italian, and Slovenian languages, respectively.

The EST-SNP marker set also confirmed 190 redundancy cases of accessions with identical names but introduced at different times in the collection (data not shown). Besides, different transcriptions of the accession names at the time of shipment (in most cases) and/or introduction at WOGBC were also observed. This would be the case for the pairs of accessions “Abadi Shlal”—“Abbadi Shalal”, “Agizi Shami”—“Aggizi Shame”, “Ensasi”—“Ansasi”, and “Masabi”—“Mossabi” sharing the same genotypes and names but with slight spelling differences (Table S3).

In addition to the above-described, this set of EST-SNPs also identified redundancies due to possible errors at different stages of germplasm sharing, conservation, and management. Thus, a total of 63 redundancy cases (representing 4.95% of the total number of accessions) were detected as possible errors.

### 2.3. Evaluation of Homonymy Cases at the WOGBC

The set of EST-SNP markers under study, in addition to their effectiveness to detect redundancies, was very useful to discriminate a significant number of homonymy cases, i.e., cultivar denominations that share the same etymological root. Thus, a total of 132 cases of homonymy (the same generic name for different olive cultivars) were identified (Table S4). In this sense, in addition to well-known homonyms, such as “Abbadi” (black fruit), “Toffahi”, and “Manzanilla” (both meaning apple-like fruit), new homonymy cases were also detected. For instance, under the generic name “local” or “from the country” were named eight different cultivars in Lebanon (“Baladi”), two cultivars in Greece (“Dopia”), as well as one cultivar from Egypt (“Balady”) and Tunisia (“Beldi”), respectively. While the fruit colour “yellow” (“Safrawi”/“Sari”/“Zard”) was used to name different cultivars in Syria, Turkey, and Iran, respectively. The names of cultivars frequently refer to the fruit shape. Thus, the generic name “round fruit” (“Yuvarlak”/“Tonda”/“Doebli”/“Redondilla”/“Ronde”) was used to denominate many cultivars in various countries, such as Turkey, Italy, Syria, Spain, Morocco, and Algeria, including either “big” (“Doebli”) or “small” (“Redondilla”, “Tondello”) fruit size cultivars (Table S4). Interestingly, when referring to their fruit shape, many olive cultivars have been named after other native Mediterranean plants, such as myrtle (“Hemblasi”), or other fruit tree species, such as dates (“Datilero(a)”/“Balah”/“Balhi”/“Hurma”), grapes (“Racimal”), pears (“Injassi”), lemon (“Limoncillo”/“Llimonenca”/“Pico Limón”), and apple (“Toffahi”/“Manzanilla”). In other cases, cultivar denominations may refer to their erect (“Alameño”) and weeping growth habit (“Chorruo”/“Llorón”/“Pendolino”), dense canopy (“Cerruda”), or high vigour (“Mawi”). Meanwhile, denominations “Yaglik”, “Yag”, “Ogliarola”, and “Sayali” refer to the main use of cultivars for “oil” or “oily”. In addition, new homonymy cases referring to the origin or the main area of olive cultivars were also observed, as in the case of the toponyms “Cordobés”/“Cordovil” and “Sinop” that included different olive cultivars from Spain (two), Portugal (two) and Turkey (three). Finally, it is interesting to mention that the word “olive” (“Elia”/“Olia”, “Zeitoun”, “Zeytin”, “Olivo”, “Ulliri”, and “Maslina”) has been frequently used to assign the names of different genotypes in many olive growing countries.

### 2.4. Assessment of Genetic Diversity and Relationships among Nonredundant Olive Cultivars

The genetic diversity of the 96 EST-SNP markers was evaluated in the nonredundant identified genotypes, 1.86 being the mean number of effective alleles per locus (N_e_) found. In general, data on allelic frequencies and other genetic parameters revealed a relatively wide diversity in the cultivars under study (Table S5). Thus, minor allele frequency (MAF) values ranged from 0.17 to 0.50, with an average value of 0.38. MAF is a measure of the discriminating ability of markers. In the case of bi-allelic markers, such as EST-SNPs, the closer the MAF is to 0.50, the better it is. In the present study, 84 out of 96 EST-SNPs displayed MAF values over 0.30 and, among them, 39 (40.6% of the total) showed MAF values ≥ 0.40, while only two EST-SNPs displayed MAF values below 0.20. Shannon’s information index (I) values ranged from 0.46 to 0.69, with the mean value of 0.65. The observed heterozygosity (H_O_) values ranged from 0.28 to 0.69, averaging 0.50, whereas the mean expected heterozygosity (H_e_) was 0.46, ranging from 0.29 to 0.50. All but four EST-SNPs showed polymorphic information content (PIC) values over 0.30.

The one-way AMOVA revealed that most of the EST-SNP diversity (90.92%) was attributable to differences among accessions within regions (western, central, and eastern Mediterranean). In fact, *ϕ_ST_* value among regions was significant (*p* < 0.0001), although very weak considering the low percentage of variance (Table S6).

**Figure 2:**
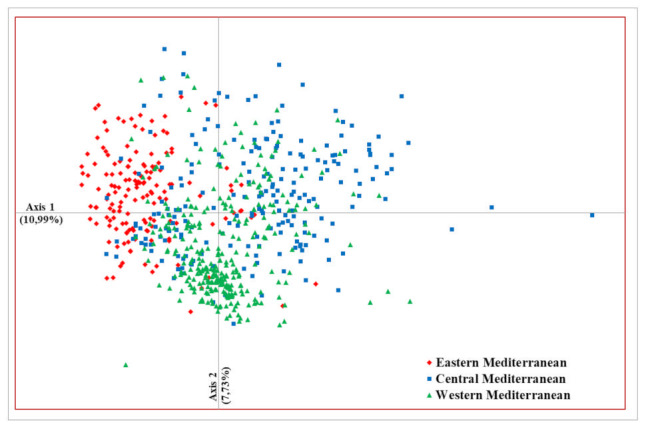
Principal co-ordinate analysis of 668 olive accessions based on 96 EST-SNP markers. Each accession is coloured according to their region of origin: eastern Mediterranean, central Mediterranean, and western Mediterranean.

The first two axes of PCoA analysis accounted for 10.99% and 7.73% of the total variance, respectively (Figure 2). Clustering by geographical origin is observed in the PCoA plot. Thus, the first axis separated the majority of olive accessions from the western Mediterranean region from those belonging to the eastern and, to a certain extent, the central Mediterranean regions. Along the second axis, the majority of accessions from the central Mediterranean region clustered separately from those belonging to the eastern one.

The STRUCTURE analysis revealed K = 3 (∆K = 738.33) as the most likely number of clusters, while the second-best solution was K = 2 (∆K = 315.67). The proportions of membership (Q) of each individual in each cluster were calculated (Figure S2 and Table S7).

**Figure 3:**
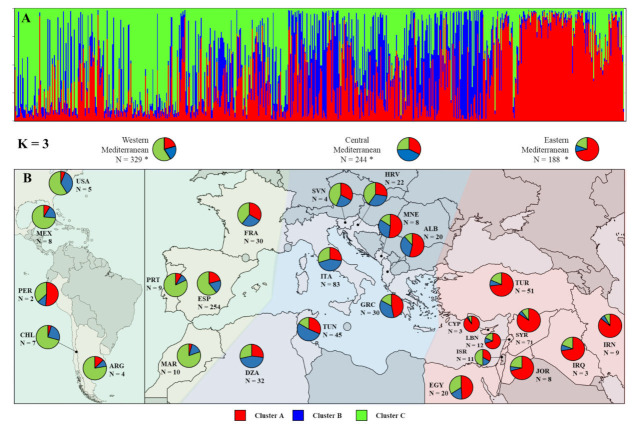
(**A**) Structure of olive genotypes from WOGBC characterized by means of EST-SNP markers following STRUCTURE analysis. Each cultivar is represented by a single vertical line divided into colours. Each colour represents one cluster, and the length of the coloured segment shows the individual’s estimated proportion of membership in that cluster. Clusters A, B, and C are associated with eastern, central, and western Mediterranean countries, respectively. (**B**) Distribution of different clusters from different countries in the Mediterranean Basin and beyond. * For each country, the total number of different cultivars was considered, regardless of synonymies between countries.

At K = 3, eastern Mediterranean accessions had an average proportion of membership assigned to cluster A of Q = 75.1% (Figure 3A and Table S7). The accessions from Cyprus (Q = 92.0%), Iran (Q = 85.2%), Syria (Q = 84.4%), and Jordan (Q = 76.2%) were mainly assigned to this cluster (Figure 3A,B). Although with a lower proportion of membership on average (Q = 44.8%), the cluster B was found mostly in accessions from central Mediterranean countries. Tunisian (Q = 55.9%), Algerian (Q = 48.5%), and Italian (Q = 41.2%) accessions were assigned in this cluster. The cluster C included accessions from western Mediterranean and from the New World (Q = 58.6%) regions. Thus, accessions from Portugal (Q = 80.4%), Mexico (Q = 75.3%), and Argentina (Q = 73.3%), followed by Morocco (Q = 64.8%) and Spain (Q = 60.8%) were assigned to this cluster.

Some accessions were assigned to different clusters than that of the region in which they were sampled and/or recorded (Table S7). In this sense, the central Mediterranean region was the most admixed one, containing accessions assigned to each of the three gene pools with the proportion of membership (Q) greater than 75%. Thus, Greek, Albanian, and Montenegrin accessions were mainly assigned to the clusters A and B. Accessions from Slovenia and Croatia were assigned to each of the three clusters, the proportion of the ones that belonged to cluster C being higher. A high level of admixture was also observed in the French olive accessions, which were assigned almost equally to all three clusters, while the accessions from Israel and Chile were assigned to two different clusters. However, all Cypriot accessions, as well as some Jordanian, Syrian, Iranian, Spanish, Tunisian, and Turkish accessions, were always assigned to the region from which they originated with a proportion of membership (Q) greater than 90%.

## 3. Discussion

### 3.1. Utility of the Set of 96 EST-SNP Markers for Olive Cultivar Identification

Management of olive germplasm collections is a complex, multidisciplinary, costly, and continuous task. Thus, selection of a reliable, practical, and cost-effective genotyping method is important, especially when a large number of accessions need to be identified [2]. In this sense, the recent experience acquired in the use of EST-SNPs for olive cultivar discrimination at the WOGBC [24] allowed the selection of a core set of 96 EST-SNP markers. In the present study, we focused on the efficiency of this set of EST-SNP markers for reliable olive cultivar discrimination and for increasing the effectiveness and accuracy of olive germplasm collections. The high reproducibility of this set of markers was expectable since they derive from a much larger but still very effective set of EST-SNP markers [24]. Besides, the observation of a clear cut-off between the highest possible intra-cultivar and the lowest inter-cultivar variability fully agrees with the low intra-cultivar mutation rates and the high stability found by means of SNP markers in previous studies in olive [22,23].

The practical utility of any molecular approach for germplasm management is determined by the ability to differentiate between a large number of accessions [2,28]. In the present study, which includes the largest number of samples analysed to date in olive, the panel of 96 EST-SNP markers allowed a thorough characterisation of all the accessions maintained at WOGBC. Their use allowed accurate identification of up to 668 nonredundant genotypes, 69.70% of them resulting as unique in that they did not match with any other accession/cultivar at WOGBC. On the other hand, among the unique accessions/cultivars identified, 136 belonged to the new plant material recently acquired at WOGBC. The identification of some unique genotypes in new olive growing areas, such as Argentina, Chile, and USA, could indicate a possible seedling selection and further spreading of the new genotypes [20] and/or the presence and preservation of “minor” local cultivars from the Mediterranean basin that have been lost or displaced by other cultivars in the original areas of diffusion in the course of centuries. These findings should have direct implications on olive breeding and germplasm conservation approach. However, as previously suggested [3,6], the identification studies should be completed by authentication of the accessions, i.e., to guarantee that the plant material hosted in the WOGBC matches with the putative original cultivar to which it belongs.

### 3.2. Assessment of Redundant Germplasm by Means of EST-SNP Markers

To ascertain the presence of duplicates in germplasm collections is as important as verifying and safeguarding as many variants as possible. In this regard, the set of 96 EST-SNPs efficiently identified the presence of redundant genotypes, i.e., accessions displaying the same SNPs profile. The largest number of duplicates was observed in the field collection (489 accessions, 48.5%), but a high level of redundancies was also detected among the new accessions (116 accessions, 43.6%). Previous studies by means of SSR markers have evidenced the presence of redundant genotypes in olive germplasm banks but in variable proportion. Thus, Muzzalupo et al. (2014) [29] reported 10.2% of redundant genotypes in an Italian olive germplasm collection, whereas Mousavi et al. (2017) [30] found 18% of duplicates within a local collection. Trujillo et al. (2014) [6] reported 33.4% duplicates in a study encompassing 499 WOGBC accessions, while the use of DArT markers [16] in the same collection revealed that redundant germplasm involved 68 out of 323 cultivars under study (around 21%). Similarly, the SSR genotyping of the international germplasm collection of Marrakech [3] revealed the presence of 41.7% of redundant germplasm among the 554 accessions under study. The unbalanced number and origin of accessions genotyped, as well as the predominant use of SSR markers which display a less pronounced difference between possible intra-cultivar versus inter-cultivar variability [3,6,8], may explain the variability range of redundant germplasm found in these studies. The efficient identification of duplicate accessions is particularly important as they represent a burden for the curators and certainly contribute to increasing the already extremely high costs of preserving olive germplasm under field conditions. In this sense, along with ongoing morpho-agronomical evaluation [31,32], passport data, and relevant information of other studies involving cultivars maintained at WOGBC, the EST-SNP results are being used to critically re-examine the composition of the collection, paying special attention to internal redundancies/duplicates. The use of this integrative information, at both cultivar and tree level, would be very useful to make cost rationalization decisions [16,24] and improve the management strategies.

In agreement with previous morphological [5,6] and molecular [3,6,16] studies, our results revealed that the redundant genotypes corresponded mainly to synonymy cases. In addition, most of them included cultivars from the same country, followed by pairs or groups of cultivars from close neighbouring countries, as previously reported [3,33,34]. However, although to a less extent, synonymy cases between cultivars from distant Mediterranean olive growing countries, as well as in the New World, have also been observed [6]. In this sense, it is worth noting that a high number of accessions (510) were identified to belong to a much lower number of cultivars (140). Such contrast may likely reflect that, during the long history of olive cultivation, a continuous interchange and human displacement of popular, successful, and probably very ancient cultivars into nearby regions or cultivation areas may have been favoured [35,36]. This process, probably boosted by the knowledge and implementation of vegetative propagation and, in particular, grafting techniques [37,38], may have also contributed to the migration of cultivars with interesting agronomic traits throughout the Mediterranean Basin and beyond [17,18,39,40]. In fact, most of the synonymy cases identified within and between olive growing countries include well-known cultivars at both a national [41,42,43,44,45,46,47] and international level [5]. The introduction of cultivars into different regions and countries was usually accompanied by their renaming according to general criteria referring to their fruit and tree morphological traits, their agronomic value, and practical utility, as well as their putative geographic location and different local customs [2,10,39]. Migration direction of cultivars is not easy to decipher, thus making it difficult to prove their exact origin. However, most of the synonymy cases (such as the synonymy group of cultivars Safrawi and Baladi, among others) reflect an east to west movement of olive cultivars in the Mediterranean Basin [36,40]. Meanwhile, the case of the synonymy group of the cultivar Frantoio may also suggest a likely west–east migration of olive cultivars due to other possible commercial routes, political, or environmental changes in the past. Besides, our results suggest that borders in agriculture are artificial, and delineating clear-cut boundaries between neighbouring and nearby olive growing areas may be an overly complicated task, testifying, thus, that olive genetic resources are (and should remain), above all, a universal heritage.

In addition to the synonyms found, and in accordance with previous studies in olive [10,37,48], our results showed that acquisition of redundant genotypes in germplasm collections may also occur through prospecting surveys in the same or close geographic areas. Besides, recollecting missions of plant material at the same locations, as well as germplasm reception from the same donor source and its further introduction at different times into the collection, may have also resulted in redundant olive germplasm [24]. At the same time, similar to previous molecular analysis in olive [6,8,16], the set of 96 EST-SNP markers has also been able to identify redundant genotypes due to possible errors in different stages of plant material acquisition, conservation, and management. On the other hand, the finding of redundancy cases also reflects the presence of duplicates within and between different olive germplasm banks (either regional or national). In fact, collaboration and sharing of germplasm with other collections during its long history as the first international olive germplasm bank, may have contributed, at least partially, to include further duplications at WOGBC [3,6]. This is probably due to the use of different criteria of sampling, the lack of representativeness of plant material, the unequal efforts on cultivar identification and characterization among the collections, and the presence of several collections per country [2,3,8].

### 3.3. Discrimination of Homonymy Cases and Naming of Olive Cultivars

The set of EST-SNP markers was very efficient for identifying many homonymy cases in the collection. The presence of numerous homonymy cases in olive germplasm, involving well-known and widely diffused cultivars, has also been reported in various previous studies [3,6,29,33,42]. The consideration of the etymological root shared by some cultivar denominations allowed us to broaden our approach to cases of homonymy, as well as to the naming of cultivars within and between olive growing countries. It seems that during the long history of olive cultivation in the Mediterranean basin and beyond, farmers may have followed the same approach for cultivar naming, resulting in both homonymy and synonymy cases [6,24,42]. Such common and universal background of cultivar naming can hardly be casual and may likely reflect a continuous exchange of information or “know how” on agricultural practices [49], as well as a common selection criterion in different olive growing areas. Thus, following the background of cultivars’ denominations it can be deduced that olive phenotypes selected by farmers mainly included agronomic traits related to productivity (“Ontha”, “Antha”), tree vigour (“Mawi”), and growth habit (“Chorruo”, “Llorón”, “Piangente”, “Alameño”), early (“Tempranillo”, “Saifi”, “Negrillo”) or late fruit ripening (“Chetoui”), fruit size (“Gordal”, “Grossa”, “Grossane”, “Esek Zeytini”–“Gaydorelia”, “Chemlal”, “Kokerrvogel”), oil and/or fruit flavour (“Meski”, “Amargoso”, “Pikrolia”, “Dulce”, “Dolce”), texture of the pulp (“Mollar”, “Ocal”), as well as their preferential use for oil (“Ogliarola”, “Rowghani”, “Sayali”, “Zaity”, “Ladoelia”), table production (“Olivo da mensa”, “Tuzlamalik”, “Salamuralik”), as pollinators (“Macho”, “Dhokkar”, “Polinizador”), or for lighting (“Llumeta”). The use of toponyms for cultivar naming is widespread in almost all olive growing areas (villages, municipalities, regions) of the world, from Iran to Mexico. This may probably reflect an empirical local selection of varieties, a restricted distribution around their possible area of origin, and a very close link with olive tree culture in each area [5,42,49]. Besides, many olive cultivars’ denominations refer to eye-catching traits like shape (“Redondilla”, “Tonda”, “Ronde”), colour of fruits (“I Bardhe”, “Bianchera”, “Bjelica”, “Beyaz”), and/or leaves (“Hojiblanca”, “Nevadillo”), as well as to local religious celebrations (“Madonna dell’Impruneta”, “Sant’ Agostino”, “Santa Caterina”, “San Francesco”, “San Pedro”) among others [41,42,43,44,45,46,47]. Eye-catching traits of olive cultivars have often been compared to other common Mediterranean animals (beetle, birds, cow, donkey, etc.) and plant or tree species (fennel, bean, myrtle, oak, aspen, etc.). In this regard, in addition to their naming referring to wild olives (“Acebuche”, “Azeboudi”, “Berri”, “Olivastra”, and “Zeboudj”), olive cultivars have also been named after other important fruit trees that were domesticated at the same time (date palm, grapes) or later (cherry, apple, lemon) than olive [50,51]. Although tracing back to the age of the olive cultivars is a difficult and complex task [10,37,38], co-ordinated and multidisciplinary studies, including genetic, archaeological, historical, and linguistic approaches [49,51,52,53,54,55], may shed some light on possible connections between cultivars names and their age, or they likely reflect a continuous and recurrent denomination process.

### 3.4. Implementation of a Protocol for Efficient Safeguard and Management of Olive Genetic Resources

The results reported herein demonstrate the utility of both the set of 96 EST-SNP markers and the genotyping method used for olive germplasm identification. At the same time, our findings support implementation of a protocol to efficiently curate an olive germplasm collection internally and raise the need of cross-cutting co-ordination and collaboration across the IOC network of germplasm banks, as previously suggested [3,6,24]. Thus, in order to collect and preserve as much olive diversity as possible, taking into account both the EST-SNP fingerprinting data of the present study and the previous experience acquired in the management of WOGBC collection [2,3,6,16,18,24,42], a specific management protocol should contemplate: (a) efficient sampling collection strategies, preferably performed during the autumn season to obtain as much plant material (fruits, stones) and information (morphology, productivity, etc.) as possible on the new accessions, (b) detailed information on passport data (location, uses, history of the plant material, accession register number at both the receptor and original collection, etc.) for each new accessions collected or received/donated, (c) ascertain and ensure the phytosanitary status of the new material through visual observations, molecular methods, and appropriate quarantine measures, (d) a priori identification by means of DNA markers of the new accessions before their introduction into the collection, (e) case-by-case revision, integrating all relevant past and present information, of the duplicates/redundant accessions detected to further discard and/or reduce their presence into the collection, (f) introduction and stewardess into the field collection of unique accessions, i.e., different cultivars identified, ensuring their maintenance and back-up at the best management condition, (g) authentication of each accession, i.e., to guarantee that it matches the DNA and/or endocarp profiles of the putative original cultivar to which it belongs, (h) documenting, storing, and managing all information related to the accessions/cultivars into an open and friendly user database. It is beyond any doubt that collections and/or reception of new accessions should comply with regional, national, and international laws on plant genetic resource protection and transfer.

### 3.5. Genetic Diversity and Relationships among Olive Cultivars

The variability range displayed by the set of 96 EST-SNPs was comparable with previous studies in olive [21,22,23] and in other fruit species [56,57]. The preliminary selection of the most polymorphic and discriminative EST-SNPs, as well as the use of a larger and diverse plant material, may explain the higher values of some diversity parameters compared to our previous study with the same type of markers in the collection [24]. However, due to their biallelic nature, these markers are usually considered to be two to five times less informative than multi-allelic microsatellites [58,59,60], highlighting the need to use a large set of SNPs to reach the same diversity levels and discriminatory power [22,56,57]. In this regard, considering that a common set of 11–17 microsatellite loci have been suggested for population studies and cultivar discrimination in olive [3,6,19], we believe that the set of 96 EST-SNP loci under study may be an optimum number of markers with equivalent efficiency to describe the real diversity presented in olive and olive germplasm collections.

In general, the goal of an ex situ olive germplasm collection is to acquire, maintain, document, assess, and make available as much genetic diversity of the crop as possible [2]. In the present study, as opposed to the redundant germplasm assessed, the identification of a high number of cultivars indicates that, although the diversity maintained at germplasm collections is, to a certain extent, overestimated, the olive crop still has a high genetic variability [3,6,24]. In this regard, the finding of a considerable level of unique germplasm, among both field and recently acquired accessions, is another striking outcome of our study. These results indicate that conservation efforts in olive should be focused both on the prioritisation of the unique accessions, either within the same country and/or at a global level [61], as well as on the prospecting of the uncovered and unknown diversity before its disappearance. In many olive growing areas, despite the richness of olive genetic patrimony, most of the olive cultivars play a local game and are being progressively displaced by a limited number of both traditional and new bred cultivars able to fulfil the requirements of the new olive growing system [2,62]. Thus, the likelihood of preservation and finding of untapped diversity in olive would be higher in those areas with less pressure of cultivar turnover and productivity. In addition, as mentioned above, new and uncatalogued diversity may also be found in olive growing countries of the New World. Accordingly, establishment of appropriate strategies for exploring and incorporating of new accessions in olive germplasm collections is fundamental to acquire the additional local olive genetic diversity, which has potential value for breeders and growers. In fact, local cultivars could be a very useful source of diversity against new or enhanced biotic and abiotic stresses associated to climatic change and in cases of outburst of new pests and diseases, such as the case of *Xylella fastidiosa*, as well as to enlarge the selection base for olive breeding programmes [2].

Both the PCoA and STRUCTURE analysis, in agreement with previous studies in olive [3,18,30,40,63], revealed a certain geographic clustering of the olive accessions under study into three main gene pools, the accessions from eastern and western Mediterranean being the best differentiated ones. These findings likely support that multi-local selection and breeding of olive cultivars occurred in each area of present diffusion, but also reflect a diversification process of cultivated olive from the east to west Mediterranean [36,53,64,65]. The high level of admixture in the central Mediterranean gene pool and the preferential clustering of many accessions with the eastern Mediterranean cultivars, as well as the clear clustering of western Mediterranean cultivars into an independent gene pool, may permit to envisage various scenarios for the development of olive cultivars in these regions: (a) an east to west dispersal pattern of olive cultivars with human migration [36,64,65]; (b) a possible local selection of wild genotypes best adapted to environmental conditions and to agronomic expectations [35,63,66]; and (c) a further breeding of cultivars introduced from abroad with local material, either wild and/or cultivated [17,18,39,40,64,67]. Local selection specifically adapted to particular environmental conditions can explain some differences between accessions and could be of great interest for olive breeding. However, the events of human selection in these areas may have been blurred during the long history of introduction and spread of eastern olive cultivars, which were later crossed with local cultivars, giving rise to further diversification [53,68]. Overall, the study of the genetic similarity among genotypes may facilitate the efficient sampling and utilization of germplasm resources by identifying unique or very distinctive gene pools, over-representations, or gaps of cultivars from certain geographic areas and the need to evaluate phenotypic variability on a restricted set of genotypes [6,8,18,69].

## 4. Materials and Methods

### 4.1. Plant Material

The plant material under study comes from the WOGBC, located at IFAPA Centre “Alameda del Obispo” in Córdoba, southern Spain (37°51′39″ N, 4°48′30″ W). It comprises accessions introduced at different times in the collection, including recently received and/or prospected ones (Table S1). Each accession is provided by a permanent and unique collection register number.

The research was carried out on 1009 WOGBC field accessions (2473 trees) planted from 1987–2016. Around half of them were characterized and identified by means of molecular markers and/or morphological descriptors in previous works [6,16,18,24,28], their identification status being continuously updated. In addition, 264 new olive accessions (considering one to three plants per accession, up to a total of 632 plants), maintained at different propagation facilities of WOGBC, were also included in the study prior to their introduction to the collection. The new accessions were obtained from international collaboration with IOC network of germplasm collection, European projects (MSCA-Before), and other regional collections of Institut de Recerca i Tecnologia Agroalimentaria (IRTA), Instituto Valenciano de Investigaciones Agrarias (IVIA), and Servicio de Investigación Agraria y Sanidad Vegetal (Gobierno de La Rioja). Besides, some of the new accessions were acquired through international (Albania, Bosnia and Herzegovina, Croatia) and ongoing local prospecting surveys.

In total, the present study was carried out in 1273 olive accessions (3105 trees/plants) from 29 different olive growing countries (Table 1 and Table S1).

### 4.2. EST-SNP Genotyping of WOGBC

For each sample under study, total genomic DNA was extracted from fresh leaves according to the CTAB method described by de la Rosa et al., 2002 [70]. DNA quantity and quality were estimated using spectrophotometry (Nanodrop 2000, Thermo Scientific, Wilmington, DE), while its integrity was assessed on 0.8% agarose gels. A core set of 96 EST-SNPs loci (Table S2) was selected from a set of 1043 EST-SNPs identified in a previous study [24] at our collection. They were selected for their discrimination capacity and amplification accuracy, each of them coming from different contigs with at least 200 bp length [27]. Based on the sequences of these selected loci, a genotyping panel of 96 SNPs type assays was further designed by Fluidigm, using its web-based Fluidigm D3™ assay design software. Fluidigm SNP genotyping was carried out following its user guide specifications. In the first step, two preamplification primers (Locus-Specific Primer (LSP) and Specific Target Amplification (STA) primer) amplified the target region containing the SNP to be genotyped. All 96 SNPs were preamplified simultaneously in one multiplex PCR, for each sample separately, on a Veriti Thermal Cycler (Applied Biosystems by ThermoFisher, Waltham, MA, USA), with the following conditions: hold at 95 °C for 15 min, 14 cycles at 95 °C for 15 s, and 60 °C for 4 min. Afterwards, an additional PCR amplified a portion of the target SNP region, using the LSP and two fluorescently labelled allele-specific internal primers ASP1 and ASP2, containing either the first or the sond allele, respectively. The sond PCR was performed on a Fluidigm 96.96 Dynamic Array IFC (Integrated Fluidic Circuit), where reactions were performed in separate nano-wells for each SNP and sample combination, allowing simultaneous genotyping of 94 samples (+2 negative test controls—NTCs) at 96 SNP loci. This PCR was performed on a BioMark HD System (Fluidigm, South San Francisco, CA, USA), with the following PCR cycling conditions: 1 cycle of Thermal Mix at 70 °C for 30 min and 25 °C for 10 min; 1 hold of Hot Sart at 95 °C for 5 min; 1 cycle of Touchdown at 95 °C for 15 s, 45 s of annealing (from 64.0 °C to 61.0 °C, dropping 1 °C per cycle), and 72 °C for 15 s; 34 cycles of additional PCR at 95 °C for 15 s, 60 °C for 45 s, and 72 °C for 15 s; and a final hold at 25 °C for 10 s. Finally, SNP genotypes were then determined by measuring the fluorescence intensity of both alleles normalised with respect to NTCs values, using SNP Genotyping Analysis Software (Fluidigm, South San Francisco, CA, USA).

Two reference cultivars (“Picual” and “Frantoio”) were included in all PCR reactions. In addition, only accessions with less than eight EST-SNP missing data were included for further analysis. As it is logical, the genotyping data obtained by the new set of 96 EST-SNP loci have considered previous identification studies by means of molecular [6,16,18,24,28] and/or morphological descriptors [5,6,42] at WOGBC. Besides, the new EST-SNP data obtained were confirmed and/or combined with passport information of the accessions, morphological, and molecular bibliographic references on cultivar´s description and discrimination, olive germplasm database [4], and, in some cases, reference material from the donor collections and/or prospecting sites. Both the field accessions and the new ones found at different propagation facilities were considered as redundant or duplicates when they shared the same EST-SNP profiles. The redundant accessions were excluded from further diversity and genetic structure analysis. In addition, for each redundancy group, a representative cultivar was selected, considering both historical identification [3,5,6,16,24,42] and passport data at WOGBC collection.

### 4.3. Data Analysis

Pairwise multi-locus matching was applied within the entire set of samples in order to measure the distance between each pair by using the GenAlex 6.5 software [71]. Key genetic parameters were calculated only on nonredundant genotypes. The following parameters were analysed: average number of alleles (*N_avg_*), number of effective alleles (N_e_), minor allele frequency (MAF), Shannon’s information index (I), and observed (H_O_) and expected heterozygosity (H_e_). Cervus v.3.0.7 software [72,73] was used to calculate the polymorphic information contents (PIC) for each EST-SNP locus.

Pairwise genetic distances, as defined by Peakall and Smouse (2012) [71], were computed using the distance procedure implemented in GenAlEx 6.5 to assess the relationships among the nonredundant genotypes. The genetic distance matrix, constructed by GenAlEx, was subjected to the analysis of molecular variance (AMOVA) approach [74] using the same program. Three Mediterranean regions were established considering the countries of origin of the different cultivars identified: (1) eastern Mediterranean (Cyprus, Egypt, Iran, Israel, Iraq, Jordan, Lebanon, Syria, and Turkey), (2) central Mediterranean (Albania, Algeria, Croatia, Greece, Italy, Montenegro, Slovenia, and Tunisia), and (3) western Mediterranean (France, Morocco, Portugal, and Spain), including New World cultivars (Argentina, Chile, Mexico, Peru, and USA). AMOVA analysis was used to partition the total genetic diversity among and within the three Mediterranean regions. Pairwise comparisons between different genotypes examined with AMOVA resulted in values of *ϕ_st_* that were equivalent to the proportion of the total variance that is partitioned between two populations/groups.

Principal co-ordinate analysis (PCoA) based on the genetic distance matrix was performed using GenAlEx 6.5 to graphically display genetic relationships among olive accessions.

A model-based clustering method was applied to infer genetic structure and to define the number of clusters using the STRUCTURE v.2.2.4 software [75]. Thirty runs of STRUCTURE were performed by setting the number of clusters (K) from 1 to 11. Each run consisted of a burn-in period of 200,000 steps, followed by 1000,000 Monte Carlo Markov Chain (MCMC) replicates, assuming an admixture model and correlated allele frequencies. No prior information was used to define the clusters. The choice of the most likely number of clusters (*K*) was carried out by comparing the average estimates of the likelihood of the data, *ln*[Pr(X|*K*)], for each value of *K*, as well as calculating an ad hoc statistic *∆K* [76] using STRUCTURE HARVESTER v. 0.6.94 [77]. Results of independent runs were clustered and averaged using Clumpak [78] to obtain the Q-value (i.e., proportion of membership) matrix. The analysis of distribution of different clusters from different countries (Figure 3B) in the Mediterranean Basin and beyond excluded countries (Bosnia and Herzegovina, Pakistan, and Uruguay) that included only one genotype.

## 5. Conclusions

This study reports the development and use of a set of 96 EST-SNP markers for the fingerprinting of the accessions maintained at the WOGBC collection. The panel of EST-SNP under study allowed the accurate identification of a high number of cultivars, the largest to date. They have also proven to be useful for the assessment of redundant germplasm and homonymy cases, thus demonstrating their utility for efficient safeguarding and management of the olive germplasm. In this sense, our findings reinforce the need of a priori identification of the new accessions to avoid the accumulation of identical material through prospecting surveys and exchange of plant material in olive germplasm collections. Besides, the thorough characterisation of the WOGBC collection by means of EST-SNP markers has enabled the implementation of a protocol to efficiently curate and safeguard olive genetic resources. The utility of this set of markers for cultivar identification, as well as the relatively wide range of variability detected, suggest their use across laboratories and germplasm collections. Thus, a global use of the SNP panel developed in the present study would not only contribute to accurate identification and removal of identical accessions within each olive germplasm collection, but also, and above all, to the discovery of the presence of identical genotypes among germplasm collections, a task still difficult in olive. In turn, co-ordinated efforts across all olive germplasm banks would also contribute to identifying globally unique genotypes whose safeguard and backup should be prioritised at both national and international olive germplasm collections. Recent efforts based on different techniques (NGS, GBS) have allowed the discovery of a considerable number of SNP markers very useful for identification, diversity, and marker-assisted selection studies in olive. Overall, the ability to integrate and combine all this information on SNP genotyping of olive cultivars with an international consortium initiative will allow the development of a public SNP database, which will have important application for efficient and cost-effective management of olive genetic resources and better safeguard of them.

## Figures and Tables

**Table 1:** Number of accessions (field and new acquired ones) genotyped per country and number of different cultivars identified in each country.

Countries	Field Accessions	New Accessions at Different Propagation Facilities	No. of Accessions	Total No. Trees/Plants	No. of Different Genotypes/Country *
Albania	20	5	25	51	20
Algeria	51		51	112	32
Argentina	6		6	13	4
Bosnia and Herzegovina	0	2	2	2	1
Chile	13		13	35	7
Croatia	24	3	27	71	22
Cyprus	11	1	12	22	3
Egypt	26	3	29	66	20
France	14	18	32	92	30
Greece	27	13	40	105	30
Iran	10		10	28	9
Iraq	0	3	3	8	3
Israel	14		14	37	11
Italy	170	1	171	408	83
Jordan	5	10	15	32	8
Lebanon	18	22	40	119	12
Mexico	8	1	9	20	8
Montenegro	8	1	9	22	8
Morocco	22		22	49	10
Pakistan	1		1	2	1
Peru	1	2	3	9	2
Portugal	11		11	22	9
Slovenia	0	4	4	6	4
Spain	330	71	401	991	254
Syria	81	37	118	301	71
Tunisia	114		114	297	45
Turkey	18	66	84	169	51
Uruguay	1	1	2	5	1
USA	5		5	11	5

* These data do not consider synonymies between different countries.

## Data Availability

Data can be made available upon reasonable request. An ongoing project on the construction of a database will facilitate data exchange in the future.

## Supplementary Materials

The following supporting information can be downloaded at: https://www.mdpi.com/article/10.3390/plants11070921/s1, Figure S1: Representation of the genetic distances among the entire set of WOGBC’s accessions. The distances were measured in number of different alleles for the pairwise comparisons among the set of accessions with 96 EST-SNPs. The small window is a zoom for the smallest distance zone among accessions; Figure S2: Log likelihood values for the data conditional of *K*, *ln* Pr(X|*K*) as suggested by Pritchard et al. (2000) [75] and on Δ*K* values; Table S1: Accessions from WOGBC (ESP-046) genotyped by means of EST-SNPs and including both field accessions and new acquired ones. The register number in the collection, name, number of trees/plants and the main area of cultivation are also indicated per each accession.; Table S2: Sequences of the sub-set of 96 EST-SNP markers used in the study; Table S3: Accessions included in each synonymy group detected by means of the 96 EST-SNP markers and previous bibliographic references; Table S4: Cultivars included in each homonymy group considered in the present study. General denomination of each homonymy group, possible meaning of homonyms´ denomination and related bibliographic references are also indicated; Bibliographic references [79,80,81,82,83,84,85,86,87,88,89,90,91,92,93,94,95,96,97,98,99,100,101,102,103,104,105,106,107,108] refer only to references related with Supplementary Tables S3 and S4; Table S5: Diversity parameters of the 96 EST-SNPs genotyped in the 668 non-redundant cultivars; Table S6: Analysis of molecular variance (AMOVA) for the partitioning of genetic variation in olive genotypes within and between Mediterranean regions (eastern/central/western); Table S7: Proportion of genome of the 668 different genotypes assigned to the three clusters (gene pools) defined with the model-based clustering method from Pritchard et al. (2000) [75].
