# Supplementary material for: Utility of EST-SNP Markers for Improving Management and Use of Olive Genetic Resources: A Case Study at the Worldwide Olive Germplasm Bank of Córdoba
Source: Plants (Basel). 2022 Mar 29;11(7):921. doi: 10.3390/plants11070921 (PMC9002360; doi:10.3390/plants11070921)
Supplement: Supplementary file 1 [file plants-11-00921-s001.zip › Fig S2_revised.pdf]

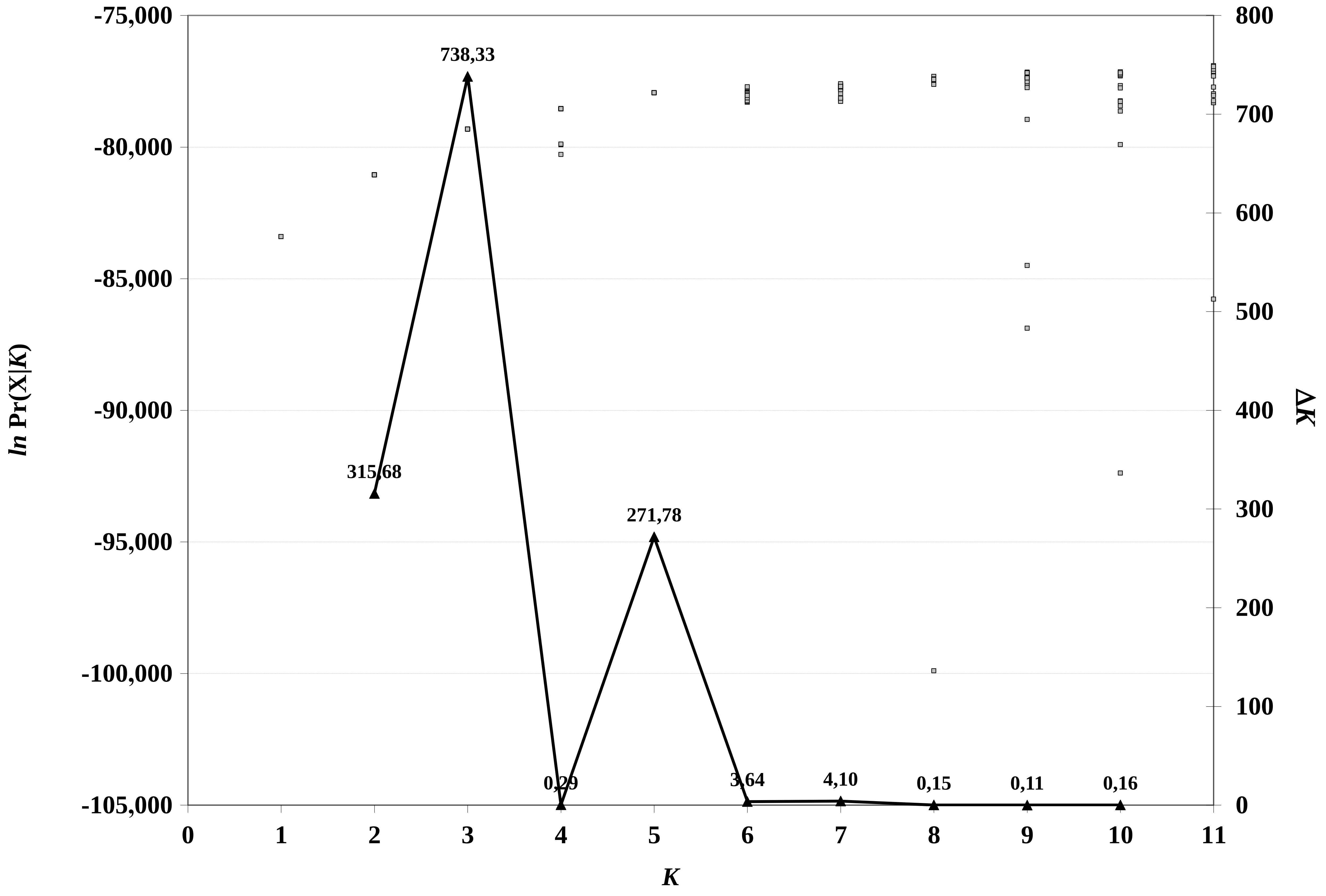

Supplemental Figure S2: Log-likelihood values for the data conditional of *K*, *ln* Pr(X|*K*), as suggested by Pritchard *et al*. (2000)[75], and on Δ*K* values.
