# Supplementary material for: Utility of EST-SNP Markers for Improving Management and Use of Olive Genetic Resources: A Case Study at the Worldwide Olive Germplasm Bank of Córdoba
Source: Plants (Basel). 2022 Mar 29;11(7):921. doi: 10.3390/plants11070921 (PMC9002360; doi:10.3390/plants11070921)
Supplement: Supplementary file 1 [file plants-11-00921-s001.zip › Figure S1.pdf]

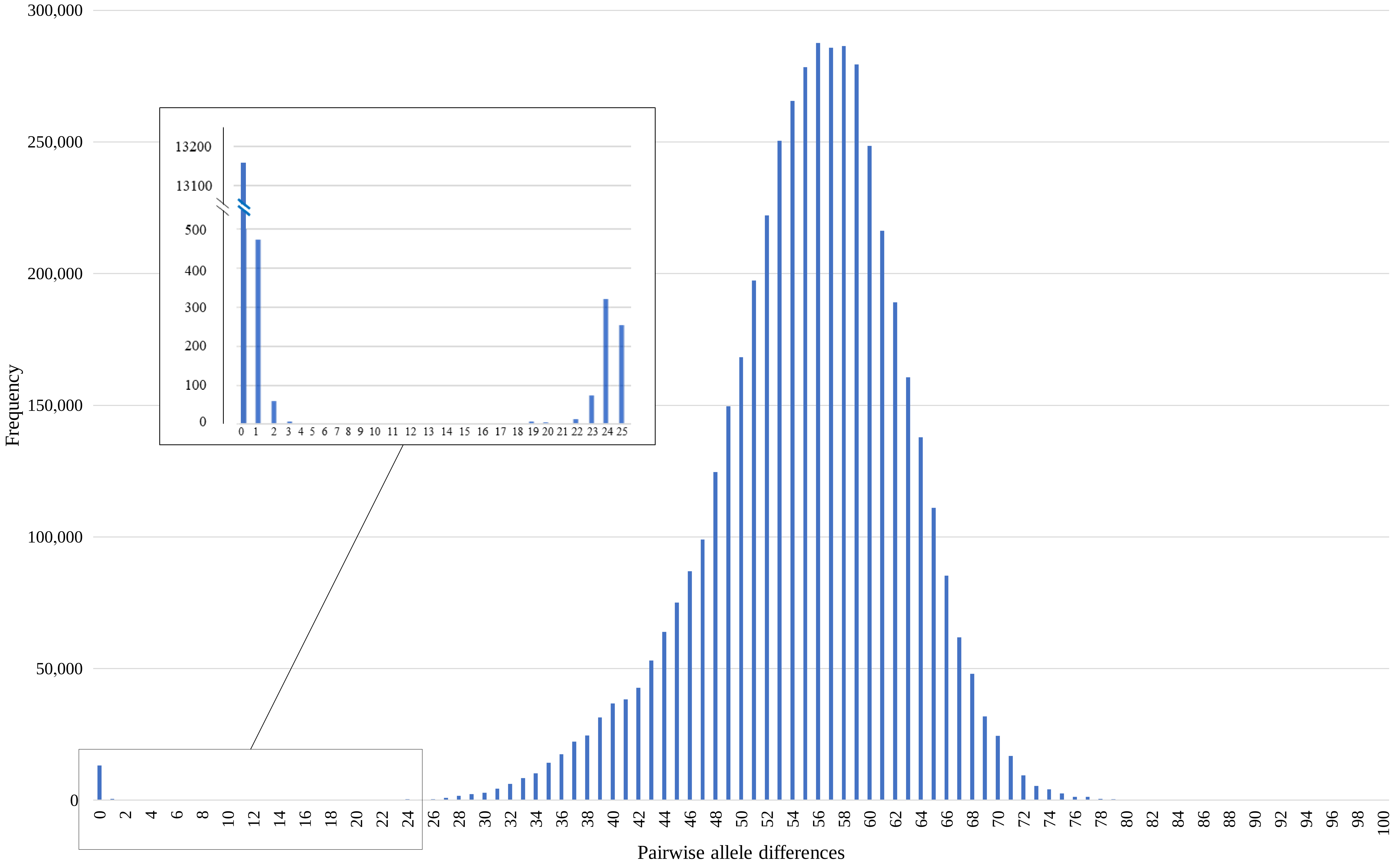

Supplemental Figure S1: Representation of the genetic distances among the entire set of WOGBC's accessions. The distances were measured in number of different alleles for the pairwise comparisons among the set of accessions with 96 EST-SNPs. The small window is a zoom for the smallest distance zone among accessions.
