# Supplementary material for: Utility of EST-SNP Markers for Improving Management and Use of Olive Genetic Resources: A Case Study at the Worldwide Olive Germplasm Bank of Córdoba
Source: Plants (Basel). 2022 Mar 29;11(7):921. doi: 10.3390/plants11070921 (PMC9002360; doi:10.3390/plants11070921)
Supplement: Supplementary file 1 [file plants-11-00921-s001.zip › Table S1_revised.pdf]

Supplemental Table S1: Accessions from WOGBC (ESP-046) genotyped by means of EST-SNPs and including both field accessions and new acquired ones. The register number in the collection, name, number of trees/plants and the main area of cultivation are also indicated per each accession.

INIA-FAO collection code	Register Number of accessions	Type of material	Accesion name	No Trees/Plants	Origin*
ESP-046	1623	New accession at propagation facilities	Abade Helo-1623	3	SYR
ESP-046	1783	Field accession	Abade Helo-1783	3	SYR
ESP-046	1615	New accession at propagation facilities	Abade Mour	3	SYR
ESP-046	1576	Field accession	Abadi Abou Chabra	2	SYR
ESP-046	1780	Field accession	Abadi Rassasi	3	SYR
ESP-046	1579	Field accession	Abadi Shlal	2	SYR
ESP-046	1540	Field accession	Abani	2	DZA
ESP-046	845	Field accession	Abbadi	2	SYR
ESP-046	1033	Field accession	Abbadi Abou Gabra-1033	2	SYR
ESP-046	842	Field accession	Abbadi Abou Gabra-842	1	SYR
ESP-046	1034	Field accession	Abbadi Shalal	2	SYR
ESP-046	1621	New accession at propagation facilities	Abd Shawka	2	SYR
ESP-046	1624	New accession at propagation facilities	Abo Akfa	1	SYR
ESP-046	1616	New accession at propagation facilities	Abo Anaked	3	SYR
ESP-046	1788	Field accession	Abo Shawke	4	SYR
ESP-046	1758	Field accession	Abou Chawkeh	3	LBN
ESP-046	1841	Field accession	Abou Chawkeh	3	ISR
ESP-046	1115	Field accession	Abou Choki-1115	2	SYR
ESP-046	1126	Field accession	Abou Choki-1126	1	SYR
ESP-046	840	Field accession	Abou Kanani	2	SYR
ESP-046	1755	Field accession	Abou Monkar	3	EGY
ESP-046	1043	Field accession	Abou Satl Mohazam	3	SYR
ESP-046	1324	Field accession	Abunara	2	ITA
ESP-046	2155	New accession at propagation facilities	Acebuche	3	ESP
ESP-046	800	Field accession	Acebuche de Caravaca-800	2	ESP
ESP-046	565	Field accession	Acebuchera	2	ESP
ESP-046	2022	Field accession	Aceitunero-2022	3	ESP

INIA-FAO collection code	Register Number of accessions	Type of material	Accesion name	No Trees/Plants	Origin*
ESP-046	2249	New accession at propagation facilities	Aceitunero-2249	3	ESP
ESP-046	102	Field accession	Adramitini	2	GRC
ESP-046	1541	Field accession	Aeleh	3	DZA
ESP-046	2329	New accession at propagation facilities	Agac no 7 (K. Maras)	1	TUR
ESP-046	1100	Field accession	Aggezi	3	EGY
ESP-046	1738	Field accession	Aggizi Akse	2	EGY
ESP-046	1739	Field accession	Aggizi Oshime	2	EGY
ESP-046	1408	Field accession	Aggizi Shame	3	EGY
ESP-046	1737	Field accession	Aggizi Shame	5	EGY
ESP-046	1525	Field accession	Aghchren de Titest	2	DZA
ESP-046	1522	Field accession	Aghenaou	2	DZA
ESP-046	1517	Field accession	Aghenfas	2	DZA
ESP-046	1360	New accession at propagation facilities	Agizi Aas	3	EGY
ESP-046	2055	Field accession	Agizi Shami	3	EGY
ESP-046	1846	Field accession	Aglandau	3	FRA
ESP-046	692	Field accession	Agouromanakolia	2	GRC
ESP-046	1377	Field accession	Agouromanakolia	3	GRC
ESP-046	1543	Field accession	Aharoune	2	DZA
ESP-046	1638	Field accession	Ahia-Ousbaa	2	DZA
ESP-046	1529	Field accession	Aimel	2	DZA
ESP-046	1645	Field accession	Aimel (Setif)	3	DZA
ESP-046	1325	Field accession	Aitana	3	ITA
ESP-046	2363	New accession at propagation facilities	Ak Zeytin	2	TUR
ESP-046	1951	Field accession	Akenane	2	DZA
ESP-046	1516	Field accession	Akerma	2	DZA
ESP-046	284	Field accession	Alameño Blanco	2	ESP
ESP-046	285	Field accession	Alameño de Cabra	2	ESP
ESP-046	254	Field accession	Alameño de Marchena	4	ESP
ESP-046	39	Field accession	Alameño de Montilla	2	ESP
ESP-046	50	Field accession	Alameño de Montilla	1	ESP

INIA-FAO collection code	Register Number of accessions	Type of material	Accesion name	No Trees/Plants	Origin*
ESP-046	2042	New accession at propagation facilities	Albareta	3	ESP
ESP-046	2246	New accession at propagation facilities	Albas	2	ESP
ESP-046	1187	Field accession	Albatro	3	ITA
ESP-046	605	Field accession	Alfafara	4	ESP
ESP-046	2041	Field accession	Alía	2	ESP
ESP-046	1188	Field accession	Allora	3	ITA
ESP-046	778	Field accession	Aloreña de Iznalloz	2	ESP
ESP-046	2283	New accession at propagation facilities	Aloreña_01	1	ESP
ESP-046	2284	New accession at propagation facilities	Aloreña_02	1	ESP
ESP-046	2285	New accession at propagation facilities	Aloreña_03	1	ESP
ESP-046	2286	New accession at propagation facilities	Aloreña_04	1	ESP
ESP-046	2287	New accession at propagation facilities	Aloreña_05	1	ESP
ESP-046	2288	New accession at propagation facilities	Aloreña_06	1	ESP
ESP-046	829	Field accession	Aloreña-829	2	ARG
ESP-046	2039	New accession at propagation facilities	Alquecerana	2	ESP
ESP-046	367	Field accession	Amargoso	6	ESP
ESP-046	1189	Field accession	Americano	2	ITA
ESP-046	1243	Field accession	Americano	2	ITA
ESP-046	2181	New accession at propagation facilities	Ampolleta	3	ESP
ESP-046	228	Field accession	Amygdalolia Nana	4	GRC
ESP-046	696	Field accession	Amygdalolia Nana	4	GRC
ESP-046	1560	New accession at propagation facilities	Ansasi	2	SYR
ESP-046	1591	New accession at propagation facilities	Antawi	3	SYR
ESP-046	2230	New accession at propagation facilities	Araban	3	FRA
ESP-046	2261	New accession at propagation facilities	Arabi Altafeleh	3	JOR
ESP-046	1807	Field accession	Arabi Altafila	1	JOR
ESP-046	1190	Field accession	Arancino	2	ITA
ESP-046	833	Field accession	Arauco	2	ARG
ESP-046	1409	Field accession	Arauco Cuatricentenario	3	ARG
ESP-046	231	Field accession	Arbequina	3	ESP

INIA-FAO collection code	Register Number of accessions	Type of material	Accesion name	No Trees/Plants	Origin*
ESP-046	1086	Field accession	Arbequina	1	ESP
ESP-046	1488	Field accession	Arbequina	7	ESP
ESP-046	666	Field accession	Arbosana	3	ESP
ESP-046	1955	Field accession	Aredhioy_2	1	CYP
ESP-046	1068	Field accession	Argudell	2	ESP
ESP-046	994	Field accession	Arroniz	2	ESP
ESP-046	1455	Field accession	Ascolana Huasco	2	CHL
ESP-046	62	Field accession	Ascolana Tenera	2	ITA
ESP-046	1244	Field accession	Ascolana Tenera	2	ITA
ESP-046	2279	New accession at propagation facilities	Ashrasse	3	IRQ
ESP-046	2368	New accession at propagation facilities	Asi Yeli	1	TUR
ESP-046	437	Field accession	Asnal	2	ESP
ESP-046	852	Field accession	Athalassa	2	CYP
ESP-046	1642	Field accession	Atounsi (Setif)	3	DZA
ESP-046	1245	Field accession	Augellina	2	ITA
ESP-046	1952	Field accession	Ayitrimihias	3	CYP
ESP-046	134	Field accession	Ayrouni	3	LBN
ESP-046	2079	New accession at propagation facilities	Ayrouni Amioun	3	LBN
ESP-046	1599	New accession at propagation facilities	Ayrouni-1599	3	SYR
ESP-046	2077	New accession at propagation facilities	Ayrouni-2077	3	LBN
ESP-046	2352	New accession at propagation facilities	Ayvalik	1	TUR
ESP-046	1456	Field accession	Azapa	3	CHL
ESP-046	381	Field accession	Azapa	5	CHL
ESP-046	726	Field accession	Azapa	4	CHL
ESP-046	1549	Field accession	Azeboudi de Kirane-1549	2	DZA
ESP-046	1659	Field accession	Azeboudi de Kirane-1659	1	DZA
ESP-046	323	Field accession	Azeitera	1	PRT
ESP-046	1530	Field accession	Azeradj	1	DZA
ESP-046	1950	Field accession	Azeradj Tamorka	3	DZA
ESP-046	343	Field accession	Azul	2	ESP

INIA-FAO collection code	Register Number of accessions	Type of material	Accesion name	No Trees/Plants	Origin*
ESP-046	959	Field accession	Azulejo	2	ESP
ESP-046	1748	Field accession	Baid El Hamam	2	EGY
ESP-046	1610	New accession at propagation facilities	Bakuri	3	SYR
ESP-046	133	Field accession	Baladi	2	LBN
ESP-046	1765	Field accession	Baladi (Ain Baal)	2	LBN
ESP-046	1757	Field accession	Baladi (Ain)	2	LBN
ESP-046	2046	New accession at propagation facilities	Baladi (Aitaroun)	3	LBN
ESP-046	1761	Field accession	Baladi (Fekhe)	5	LBN
ESP-046	1773	Field accession	Baladi (Qana)	5	LBN
ESP-046	1764	Field accession	Baladi (Koura)	4	LBN
ESP-046	2086	New accession at propagation facilities	Baladi Deir Aamass	3	LBN
ESP-046	2092	New accession at propagation facilities	Baladi Deir Memass	1	LBN
ESP-046	2075	New accession at propagation facilities	Baladi Hasrout	4	LBN
ESP-046	2072	New accession at propagation facilities	Baladi Hermel-2072	3	LBN
ESP-046	2074	New accession at propagation facilities	Baladi Hermel-2074	3	LBN
ESP-046	2085	New accession at propagation facilities	Baladi Ijd Ibrine	3	LBN
ESP-046	2090	New accession at propagation facilities	Baladi Janata	3	LBN
ESP-046	2087	New accession at propagation facilities	Baladi Jowaya (Qana)	3	LBN
ESP-046	2078	New accession at propagation facilities	Baladi Kfarzaina	3	LBN
ESP-046	1966	Field accession	Baladi Mabroum	3	LBN
ESP-046	2045	Field accession	Baladi Mabroum	3	LBN
ESP-046	2084	New accession at propagation facilities	Baladi Qana	3	LBN
ESP-046	1769	Field accession	Baladi Remadieh-1769	2	LBN
ESP-046	2082	New accession at propagation facilities	Baladi Remadieh-2082	2	LBN
ESP-046	1759	Field accession	Baladi Roumani	3	LBN
ESP-046	1756	Field accession	Baladi Tawil	2	LBN
ESP-046	2080	New accession at propagation facilities	Baladi Zgharta	3	LBN
ESP-046	1744	Field accession	Balady	2	EGY
ESP-046	2073	New accession at propagation facilities	Balah	4	LBN
ESP-046	1545	Field accession	Balbal	4	DZA

INIA-FAO collection code	Register Number of accessions	Type of material	Accesion name	No Trees/Plants	Origin*
ESP-046	1916	Field accession	Balhi Sig	4	TUN
ESP-046	711	Field accession	Barnea	2	ISR
ESP-046	1661	Field accession	Barouni-1661	2	TUN
ESP-046	1823	Field accession	Barouni-1823	2	TUN
ESP-046	1026	Field accession	Barri	3	SYR
ESP-046	1808	Field accession	Bathni	1	JOR
ESP-046	2258	New accession at propagation facilities	Bathni	3	JOR
ESP-046	1922	Field accession	Becarut	3	ESP
ESP-046	1561	New accession at propagation facilities	Bed al Igel	3	SYR
ESP-046	1663	Field accession	Beldi	3	TUN
ESP-046	2221	New accession at propagation facilities	Belgentieroise	3	FRA
ESP-046	1246	Field accession	Bella di Spagna	2	ITA
ESP-046	690	Field accession	Belluti	3	TUR
ESP-046	1466	Field accession	Belluti	1	TUR
ESP-046	1029	Field accession	Bent al Kadi	1	SYR
ESP-046	1927	Field accession	Berri Meslal 3	1	MAR
ESP-046	1662	Field accession	Besbessi-1662	3	TUN
ESP-046	1870	Field accession	Besbessi-1870	3	TUN
ESP-046	683	Field accession	Beyaz Yaglik	2	TUR
ESP-046	1753	Field accession	Bez el Anza	4	EGY
ESP-046	1247	Field accession	Bianchera	2	ITA
ESP-046	1248	Field accession	Biancolilla	2	ITA
ESP-046	1814	Field accession	Biancolilla-1814	1	ARG
ESP-046	387	Field accession	Bical	2	ESP
ESP-046	1917	Field accession	Bidh Hmam	3	TUN
ESP-046	2242	New accession at propagation facilities	Biequeruda	3	ESP
ESP-046	1768	Field accession	Bissani	4	LBN
ESP-046	1975	Field accession	Bjelica	3	HRV
ESP-046	2245	Field accession	Blancal	1	ESP
ESP-046	11	Field accession	Blanqueta	3	ESP

INIA-FAO collection code	Register Number of accessions	Type of material	Accesion name	No Trees/Plants	Origin*
ESP-046	48	Field accession	Blanqueta	2	ESP
ESP-046	2172	New accession at propagation facilities	Blanqueta Gorda	3	ESP
ESP-046	1550	Field accession	Blanquette de Gastu	2	DZA
ESP-046	1532	Field accession	Blanquette de Guelma	2	DZA
ESP-046	631	Field accession	Bodoquera	2	ESP
ESP-046	18	Field accession	Bolvino	2	ESP
ESP-046	46	Field accession	Bolvino	2	ESP
ESP-046	57	Field accession	Bolvino	4	ESP
ESP-046	641	Field accession	Bolvino	2	ESP
ESP-046	41	Field accession	Borriolenca	2	ESP
ESP-046	611	Field accession	Borriolenca	2	ESP
ESP-046	1170	Field accession	Bosana	2	ITA
ESP-046	1327	Field accession	Bottone di Gallo	3	ITA
ESP-046	1937	Field accession	Bouchouika	1	MAR
ESP-046	1542	Field accession	Bouchouk Lafayette	3	DZA
ESP-046	1938	Field accession	Bouchouk Laghlid	2	MAR
ESP-046	1512	Field accession	Bouchouk Soummam 1	2	DZA
ESP-046	1546	Field accession	Bouchoukra	2	DZA
ESP-046	1518	Field accession	Boughenfous	2	DZA
ESP-046	1528	Field accession	Boukaila	2	DZA
ESP-046	1655	Field accession	Bouricha	4	DZA
ESP-046	63	Field accession	Bouteillan	2	FRA
ESP-046	1174	Field accession	Brandofino	3	ITA
ESP-046	1328	Field accession	Brandofino	3	ITA
ESP-046	1475	Field accession	Branquita de Elvas Red	2	PRT
ESP-046	2276	New accession at propagation facilities	Brava	3	ESP
ESP-046	733	Field accession	Buga	2	HRV
ESP-046	2271	New accession at propagation facilities	Buga	1	SVN
ESP-046	2387	New accession at propagation facilities	Buharica	2	HRV
ESP-046	255	Field accession	Buidiego	1	ESP

INIA-FAO collection code	Register Number of accessions	Type of material	Accesion name	No Trees/Plants	Origin*
ESP-046	1159	Field accession	Buidiego	2	ESP
ESP-046	2295	New accession at propagation facilities	Butko	3	TUR
ESP-046	2318	New accession at propagation facilities	Buyuk Topak Ulak	1	TUR
ESP-046	1987	Field accession	Buža	3	HRV
ESP-046	333	Field accession	Caballo	5	ESP
ESP-046	1847	Field accession	Cailleitier	2	FRA
ESP-046	1751	Field accession	Cairo_7	2	EGY
ESP-046	1094	Field accession	Cairo_7	3	EGY
ESP-046	96	Field accession	Cakir	1	TUR
ESP-046	1329	Field accession	Calatina	2	ITA
ESP-046	2222	New accession at propagation facilities	Calian	3	FRA
ESP-046	2171	New accession at propagation facilities	Calles_1	3	ESP
ESP-046	24	Field accession	Callosina	4	ESP
ESP-046	40	Field accession	Callosina	2	ESP
ESP-046	60	Field accession	Callosina	2	ESP
ESP-046	653	Field accession	Canetera	2	ESP
ESP-046	77	Field accession	Caninese	4	ITA
ESP-046	1163	Field accession	Caninese	2	ITA
ESP-046	52	Field accession	Cañivano Blanco	2	ESP
ESP-046	256	Field accession	Cañivano Negro	2	ESP
ESP-046	259	Field accession	Cañivano Negro	2	ESP
ESP-046	55	Field accession	Cañivano Negro-55	1	ESP
ESP-046	1319	Field accession	Cariasina	2	ITA
ESP-046	736	Field accession	Carolea	2	ITA
ESP-046	1249	Field accession	Carolea	2	ITA
ESP-046	2180	New accession at propagation facilities	Carrasco	3	ESP
ESP-046	1372	Field accession	Carrasqueña de Cañada	3	ESP
ESP-046	1457	Field accession	Carrasqueña Huasco	2	CHL
ESP-046	494	Field accession	Carrasqueño de Alburquerque	2	ESP
ESP-046	332	Field accession	Carrasqueño de Alcaudete	2	ESP

INIA-FAO collection code	Register Number of accessions	Type of material	Accesion name	No Trees/Plants	Origin*
ESP-046	125	Field accession	Carrasqueño de Elvas	2	PRT
ESP-046	591	Field accession	Carrasqueño de Jumilla	2	ESP
ESP-046	286	Field accession	Carrasqueño de la Sierra	2	ESP
ESP-046	795	Field accession	Carrasqueño de Porcuna	2	ESP
ESP-046	2196	New accession at propagation facilities	Carrasqueta de Ayora	3	ESP
ESP-046	785	Field accession	Carrasquillo	2	ESP
ESP-046	2248	Field accession	Caspolina	2	ESP
ESP-046	950	Field accession	Castacabra	1	ESP
ESP-046	576	Field accession	Castellana	3	ESP
ESP-046	1331	Field accession	Castriciana Rapparina	3	ITA
ESP-046	1332	Field accession	Cavalieri	2	ITA
ESP-046	2228	New accession at propagation facilities	Cayet Noir	1	FRA
ESP-046	2205	New accession at propagation facilities	Cayet Roux	3	FRA
ESP-046	1848	Field accession	Cayon	2	FRA
ESP-046	2364	New accession at propagation facilities	Cekiste	3	TUR
ESP-046	2317	New accession at propagation facilities	Celebi (Iznik)	1	TUR
ESP-046	2321	New accession at propagation facilities	Celebi (Silifke)	1	TUR
ESP-046	1333	Field accession	Cerasuola	3	ITA
ESP-046	331	Field accession	Cerezuela	1	ESP
ESP-046	2214	New accession at propagation facilities	Cerisier	3	FRA
ESP-046	2036	Field accession	Cerruda de Liesa	3	ESP
ESP-046	2034	Field accession	Cerruda de Olvena	3	ESP
ESP-046	1668	Field accession	Chaïbi Antha	3	TUN
ESP-046	220	Field accession	Chalkidikis	4	GRC
ESP-046	1450	Field accession	Changlot	3	ESP
ESP-046	15	Field accession	Changlot Real	3	ESP
ESP-046	42	Field accession	Changlot Real	3	ESP
ESP-046	1556	Field accession	Chemchali	1	TUN
ESP-046	1673	Field accession	Chemchali	2	TUN
ESP-046	1878	Field accession	Chemchali Gafsa	2	TUN

INIA-FAO collection code	Register Number of accessions	Type of material	Accesion name	No Trees/Plants	Origin*
ESP-046	1526	Field accession	Chemlal	2	DZA
ESP-046	118	Field accession	Chemlal de Kabilye	3	DZA
ESP-046	1827	Field accession	Chemlali Balhi	2	TUN
ESP-046	1874	Field accession	Chemlali Bent Louzir	3	TUN
ESP-046	1912	Field accession	Chemlali Ghraiba	4	TUN
ESP-046	1892	Field accession	Chemlali Mahares	4	TUN
ESP-046	1910	Field accession	Chemlali Meliane	2	TUN
ESP-046	1552	Field accession	Chemlali Nord	1	TUN
ESP-046	1555	Field accession	Chemlali Nord	1	TUN
ESP-046	1865	Field accession	Chemlali Nord	3	TUN
ESP-046	1899	Field accession	Chemlali Ontha Tataouine	3	TUN
ESP-046	1914	Field accession	Chemlali Ouled Msallem	4	TUN
ESP-046	1894	Field accession	Chemlali Ouled Youssef	3	TUN
ESP-046	1507	Field accession	Chemlali Sfax	1	TUN
ESP-046	1682	Field accession	Chemlali Sfax	3	TUN
ESP-046	1871	Field accession	Chemlali Sfax	3	TUN
ESP-046	1873	Field accession	Chemlali Sig	3	TUN
ESP-046	1901	Field accession	Chemlali Tataouine	2	TUN
ESP-046	1825	Field accession	Chemlali Zarzis	2	TUN
ESP-046	1880	Field accession	Chemlali Zarzis	4	TUN
ESP-046	374	Field accession	Chesna	2	ESP
ESP-046	113	Field accession	Chetoui	2	TUN
ESP-046	744	Field accession	Chetoui	2	TUN
ESP-046	1664	Field accession	Chetoui	3	TUN
ESP-046	1678	Field accession	Chetoui Tunis	1	TUN
ESP-046	1723	Field accession	Chetoui Tunis	1	TUN
ESP-046	1868	Field accession	Chetoui Tunis-1868	4	TUN
ESP-046	1632	Field accession	Chetous	1	DZA
ESP-046	1920	Field accession	Chiquitita	2	ESP
ESP-046	2206	New accession at propagation facilities	Chondrolia	3	GRC

INIA-FAO collection code	Register Number of accessions	Type of material	Accesion name	No Trees/Plants	Origin*
ESP-046	402	Field accession	Chorreao de Montefrio	2	ESP
ESP-046	29	Field accession	Chorrúo	2	ESP
ESP-046	361	Field accession	Chorrúo de Castro del Río-361	2	ESP
ESP-046	1191	Field accession	Ciliegino	3	ITA
ESP-046	2356	New accession at propagation facilities	Cilli	2	TUR
ESP-046	1250	Field accession	Cima di Melfi	2	ITA
ESP-046	90	Field accession	Cipresino	2	ITA
ESP-046	634	Field accession	Cirujal	2	ESP
ESP-046	2027	Field accession	Cirujal-2027	3	ESP
ESP-046	2153	New accession at propagation facilities	Cirujal-2153	3	ESP
ESP-046	124	Field accession	Cobrancosa	2	PRT
ESP-046	1316	Field accession	Confetto	2	ITA
ESP-046	79	Field accession	Coratina	2	ITA
ESP-046	1251	Field accession	Coratina	2	ITA
ESP-046	645	Field accession	Corbella	3	ESP
ESP-046	659	Field accession	Corbella	1	ESP
ESP-046	817	Field accession	Corbella-817	5	ESP
ESP-046	514	Field accession	Cordobés de Arroyo de la Luz	2	ESP
ESP-046	879	Field accession	Cordobés de la Aliseda	2	ESP
ESP-046	886	Field accession	Cordovil de Castelo Branco	2	PRT
ESP-046	131	Field accession	Cordovil de Serpa	2	PRT
ESP-046	511	Field accession	Corneja	2	ESP
ESP-046	20	Field accession	Cornezuelo de Jaén	2	ESP
ESP-046	10	Field accession	Cornicabra	2	ESP
ESP-046	957	Field accession	Cornicabra de Jerez Caballeros	2	ESP
ESP-046	522	Field accession	Cornicabra de Mérida	2	ESP
ESP-046	1087	Field accession	Cornicabra Murciana	2	ESP
ESP-046	790	Field accession	Corralones de Andújar	2	ESP
ESP-046	1721	Field accession	Corralones de Andújar	2	ESP
ESP-046	1192	Field accession	Correggiolo di Pallesse	3	ITA

INIA-FAO collection code	Register Number of accessions	Type of material	Accesion name	No Trees/Plants	Origin*
ESP-046	1320	Field accession	Corsicana da Mensa	3	ITA
ESP-046	1321	Field accession	Corsicana da Olio	2	ITA
ESP-046	1252	Field accession	Craputea	2	ITA
ESP-046	1334	Field accession	Crastu	2	ITA
ESP-046	734	Field accession	Crnica	2	HRV
ESP-046	2274	New accession at propagation facilities	Crnica	1	SVN
ESP-046	1976	Field accession	Crnica-1976	3	HRV
ESP-046	2000	Field accession	Crnjaka	3	MNE
ESP-046	1193	Field accession	Cucca	1	ITA
ESP-046	1194	Field accession	Cuoricino	2	ITA
ESP-046	1440	Field accession	Cuquello de la Jana	2	ESP
ESP-046	2170	New accession at propagation facilities	Cuquello de la Jana	3	ESP
ESP-046	2189	New accession at propagation facilities	Cuquello_1	3	ESP
ESP-046	1072	Field accession	Curivell	2	ESP
ESP-046	2229	New accession at propagation facilities	Curnet	1	FRA
ESP-046	1099	New accession at propagation facilities	D. de Egipto	3	EGY
ESP-046	1380	New accession at propagation facilities	Dafnelia	2	GRC
ESP-046	2280	New accession at propagation facilities	Dahgan	3	IRQ
ESP-046	2067	New accession at propagation facilities	Dal	3	LBN
ESP-046	1585	Field accession	Dan	3	SYR
ESP-046	136	Field accession	Dan-136	2	SYR
ESP-046	2186	New accession at propagation facilities	Datilera de Caudiel	2	ESP
ESP-046	403	Field accession	Datilero	5	ESP
ESP-046	420	Field accession	De Sal	1	ESP
ESP-046	1940	Field accession	Dehbia	3	MAR
ESP-046	1609	New accession at propagation facilities	Delle	3	SYR
ESP-046	1634	Field accession	Derdi	3	DZA
ESP-046	1667	Field accession	Derras	2	TUN
ESP-046	2277	New accession at propagation facilities	Desconocida Vx1	2	ESP
ESP-046	1481	Field accession	Desconocida-1481	3	ESP

INIA-FAO collection code	Register Number of accessions	Type of material	Accesion name	No Trees/Plants	Origin*
ESP-046	2021	Field accession	Desconocido-2021	2	ESP
ESP-046	2026	Field accession	Desconocido-2026	3	ESP
ESP-046	1853	Field accession	Dezfool	3	IRN
ESP-046	1898	Field accession	Dhokkar Nafti	3	TUN
ESP-046	1511	Field accession	Dhokkar Tataouine	3	TUN
ESP-046	1686	Field accession	Dhokkar Tataouine	3	TUN
ESP-046	1734	Field accession	Dhokkar Tataouine	4	TUN
ESP-046	1900	Field accession	Dhokkar Tataouine	2	TUN
ESP-046	1717	Field accession	Diferente	3	ESP
ESP-046	2360	New accession at propagation facilities	Dilmit	2	TUR
ESP-046	1572	Field accession	Djabali Kini-1572	3	SYR
ESP-046	2057	New accession at propagation facilities	Djabali Kini-2057	3	SYR
ESP-046	1044	Field accession	Doebli	3	SYR
ESP-046	1596	New accession at propagation facilities	Doebli-1596	3	SYR
ESP-046	539	Field accession	Dokkar	4	TUN
ESP-046	2017	Field accession	Dolça	3	ESP
ESP-046	724	Field accession	Dolce	2	EGY
ESP-046	65	Field accession	Dolce Agogia	2	ITA
ESP-046	1363	Field accession	Dolce Agogia	2	ITA
ESP-046	94	Field accession	Domat	2	TUR
ESP-046	2234	New accession at propagation facilities	Dopia Pierias	3	GRC
ESP-046	2238	New accession at propagation facilities	Dopia Verias	3	GRC
ESP-046	1594	Field accession	Drmlali	3	SYR
ESP-046	1990	Field accession	Drobnica	3	HRV
ESP-046	1992	Field accession	Drobnica	3	MNE
ESP-046	352	Field accession	Dulzal	4	ESP
ESP-046	31	Field accession	Dulzal de Carmona	6	ESP
ESP-046	1998	Field accession	Dužica	3	MNE
ESP-046	707	Field accession	Dwarf D	2	USA
ESP-046	1830	Field accession	Ech Chahla	4	TUN

INIA-FAO collection code	Register Number of accessions	Type of material	Accesion name	No Trees/Plants	Origin*
ESP-046	2311	New accession at propagation facilities	Edincik	3	TUR
ESP-046	97	Field accession	Edremit Yaglik	3	TUR
ESP-046	2338	New accession at propagation facilities	Egriburum (Nizip)	1	TUR
ESP-046	2340	New accession at propagation facilities	Egriburum (Tatayn)	3	TUR
ESP-046	1849	Field accession	El Chatan	3	DZA
ESP-046	1850	Field accession	El Kharfi	3	DZA
ESP-046	1745	Field accession	El Salam	2	EGY
ESP-046	686	Field accession	Elmacik	2	TUR
ESP-046	1195	Field accession	Emilia	3	ITA
ESP-046	13	Field accession	Empeltre	3	ESP
ESP-046	586	Field accession	Enagua de Arenas	2	ESP
ESP-046	1124	Field accession	Ensasi	2	SYR
ESP-046	681	Field accession	Erdek Yaglik	2	TUR
ESP-046	2355	New accession at propagation facilities	Erkence	2	TUR
ESP-046	779	Field accession	Escarabajillo	2	ESP
ESP-046	1720	Field accession	Escarabajo	2	ESP
ESP-046	408	Field accession	Escarabajuelo de Atarfe	2	ESP
ESP-046	293	Field accession	Escarabajuelo de Posadas	2	ESP
ESP-046	353	Field accession	Escarabajuelo de Úbeda	2	ESP
ESP-046	2312	New accession at propagation facilities	Esek zeytini (Odemis)	2	TUR
ESP-046	2309	New accession at propagation facilities	Eşek zeytini (Tekirdag)	1	TUR
ESP-046	1957	Field accession	Evrihoy_1	1	CYP
ESP-046	1904	Field accession	Fakhari	2	TUN
ESP-046	1928	Field accession	Fakhfoukha	3	MAR
ESP-046	1839	Field accession	Falsa Gordal Sevillana	2	URU
ESP-046	1253	Field accession	Faresana	2	ITA
ESP-046	12	Field accession	Farga	2	ESP
ESP-046	1787	Field accession	Faruke	1	SYR
ESP-046	1254	Field accession	Fasolina	2	ITA
ESP-046	1255	Field accession	Fasolona	2	ITA

INIA-FAO collection code	Register Number of accessions	Type of material	Accesion name	No Trees/Plants	Origin*
ESP-046	1514	Field accession	Ferkani	2	DZA
ESP-046	822	Field accession	Figueretes	2	ESP
ESP-046	1196	Field accession	Filare	2	ITA
ESP-046	1197	Field accession	Firenzuolo	3	ITA
ESP-046	1141	Field accession	Fishomi	3	IRN
ESP-046	851	Field accession	Flasou	1	CYP
ESP-046	1958	Field accession	Flasoy	1	CYP
ESP-046	652	Field accession	Forastera de Tortosa	2	ESP
ESP-046	1675	Field accession	Fouji Asli Gafsa-1675	1	TUN
ESP-046	1884	Field accession	Fouji Asli Gafsa-1884	3	TUN
ESP-046	1666	Field accession	Fouji Vert	3	TUN
ESP-046	1367	Field accession	Fraga	3	ESP
ESP-046	1995	Field accession	Fram	2	MNE
ESP-046	80	Field accession	Frantoio	3	ITA
ESP-046	179	Field accession	Frantoio	2	ITA
ESP-046	723	Field accession	Frantoio	2	ITA
ESP-046	1038	Field accession	Frantoio	3	ITA
ESP-046	1080	Field accession	Frantoio	2	ITA
ESP-046	1198	Field accession	Frantoio	2	ITA
ESP-046	1256	Field accession	Frantoio	2	ITA
ESP-046	81	Field accession	Frantoio A. Corsini	2	ITA
ESP-046	2496	New accession at propagation facilities	FS17	3	ITA
ESP-046	656	Field accession	Fulla de Salze	2	ESP
ESP-046	674	Field accession	Fulla de Salze	2	ESP
ESP-046	2207	New accession at propagation facilities	Galatistas	3	GRC
ESP-046	128	Field accession	Galega Vulgar	2	PRT
ESP-046	380	Field accession	Gatuno	2	ESP
ESP-046	695	Field accession	Gaydoyrelia	2	GRC
ESP-046	92	Field accession	Gemlik	2	TUR
ESP-046	2064	New accession at propagation facilities	General Hornos	3	URU

INIA-FAO collection code	Register Number of accessions	Type of material	Accesion name	No Trees/Plants	Origin*
ESP-046	970	Field accession	Genovesa	2	ESP
ESP-046	1725	Field accession	Gerboua	1	TUN
ESP-046	538	Field accession	Gerboui	3	TUN
ESP-046	1551	Field accession	Gerboui-1551	1	TUN
ESP-046	1672	Field accession	Gerboui-1672	3	TUN
ESP-046	1862	Field accession	Gerboui-1862	3	TUN
ESP-046	1854	Field accession	Gerdeh	3	IRN
ESP-046	1438	New accession at propagation facilities	Getsemani	3	MEX
ESP-046	1336	Field accession	Giarraffa	2	ITA
ESP-046	2199	New accession at propagation facilities	Gileta	3	ESP
ESP-046	1199	Field accession	Ginestrino	2	ITA
ESP-046	1200	Field accession	Giogolino	3	ITA
ESP-046	2361	New accession at propagation facilities	Girit Zeytini	1	TUR
ESP-046	1084	Field accession	Gjykatesi	2	ALB
ESP-046	1365	Field accession	Gorda Limoncillo	3	ESP
ESP-046	755	Field accession	Gordal de Archidona	2	ESP
ESP-046	261	Field accession	Gordal de Écija	2	ESP
ESP-046	761	Field accession	Gordal de Granada	2	ESP
ESP-046	567	Field accession	Gordal de Hellín	4	ESP
ESP-046	804	Field accession	Gordal de Hellín	2	ESP
ESP-046	2044	Field accession	Gordal de Somontano	3	ESP
ESP-046	781	Field accession	Gordal de Vélez Rubio	2	ESP
ESP-046	234	Field accession	Gordal Sevillana	2	ESP
ESP-046	1467	Field accession	Gordal Sevillana	3	ESP
ESP-046	1127	Field accession	Gordal Valle las Palmeras	3	MEX
ESP-046	396	Field accession	Gordalejo	3	ESP
ESP-046	2291	New accession at propagation facilities	Gorvele	3	TUR
ESP-046	181	Field accession	Grappolo	1	ITA
ESP-046	1201	Field accession	Grappolo	3	ITA
ESP-046	1458	Field accession	Grappolo Limari	2	CHL

INIA-FAO collection code	Register Number of accessions	Type of material	Accesion name	No Trees/Plants	Origin*
ESP-046	2210	New accession at propagation facilities	Grassois	3	FRA
ESP-046	1202	Field accession	Gremignolo	2	ITA
ESP-046	1203	Field accession	Gremignolo di Bolgheri	3	ITA
ESP-046	680	Field accession	Girit Zeytini	2	TUR
ESP-046	1994	Field accession	Groginja	3	MNE
ESP-046	811	Field accession	Grosal de Albocácer	2	ESP
ESP-046	802	Field accession	Grosal de Cieza	1	ESP
ESP-046	1069	Field accession	Grosal Vimbodí	2	ESP
ESP-046	1258	Field accession	Grossa di Spagna	2	ITA
ESP-046	1845	Field accession	Grossanne	3	FRA
ESP-046	67	Field accession	Grossanne-67	3	FRA
ESP-046	1527	Field accession	Grosse du Hamma	1	DZA
ESP-046	1204	Field accession	Grossolana	2	ITA
ESP-046	295	Field accession	Habichuelero de Baena	2	ESP
ESP-046	2137	New accession at propagation facilities	Habichuelero de Baena	2	ESP
ESP-046	2163	New accession at propagation facilities	Habichuelero de Baena	3	ESP
ESP-046	366	Field accession	Habichuelero de Grazalema	4	ESP
ESP-046	2322	New accession at propagation facilities	Halhali (Hatay)	1	TUR
ESP-046	2332	New accession at propagation facilities	Halhali Celebi	3	TUR
ESP-046	2168	Field accession	Hamid	1	EGY
ESP-046	1538	Field accession	Hamra	2	DZA
ESP-046	1942	Field accession	Hamrani	2	MAR
ESP-046	2333	New accession at propagation facilities	Hamza Celebi	2	TUR
ESP-046	1608	New accession at propagation facilities	Hanefa	3	SYR
ESP-046	835	Field accession	Haouzia	2	MAR
ESP-046	2054	Field accession	Hembalassi	3	SYR
ESP-046	1567	New accession at propagation facilities	Hembalassi-1567	3	SYR
ESP-046	1002	Field accession	Hemblasi	2	SYR
ESP-046	2164	New accession at propagation facilities	Hendero	2	ESP
ESP-046	2157	New accession at propagation facilities	Hijarol	3	ESP

INIA-FAO collection code	Register Number of accessions	Type of material	Accesion name	No Trees/Plants	Origin*
ESP-046	2053	Field accession	Hiloso	3	ESP
ESP-046	2342	New accession at propagation facilities	Hirhali Celebi	1	TUR
ESP-046	2	Field accession	Hojiblanca	5	ESP
ESP-046	59	Field accession	Hojiblanca	2	ESP
ESP-046	1373	Field accession	Hojiblanca de Navarrés	3	ESP
ESP-046	1559	New accession at propagation facilities	Humaisi	3	SYR
ESP-046	2354	New accession at propagation facilities	Hurma Kaba	2	TUR
ESP-046	2353	New accession at propagation facilities	Hurma Karaca	1	TUR
ESP-046	2347	New accession at propagation facilities	Hursuki	1	TUR
ESP-046	1557	Field accession	Idleb_1	3	SYR
ESP-046	1792	Field accession	Idleb_3	4	SYR
ESP-046	1547	Field accession	Ifiri	2	DZA
ESP-046	2051	New accession at propagation facilities	Ileña_1	3	PER
ESP-046	297	Field accession	Imperial	1	ESP
ESP-046	30	Field accession	Imperial de Jaén	2	ESP
ESP-046	1889	Field accession	Injassi Gafsa	4	TUN
ESP-046	1893	Field accession	Injassi Hchichina	2	TUN
ESP-046	2343	New accession at propagation facilities	Iri Yuvarlak	3	TUR
ESP-046	735	Field accession	Istarska Bjelica	2	HRV
ESP-046	1988	Field accession	Istarska Bjelica	3	HRV
ESP-046	1983	Field accession	Istarska Crnica	3	HRV
ESP-046	2272	New accession at propagation facilities	Istrka Belica	1	SVN
ESP-046	68	Field accession	Itrana	4	ITA
ESP-046	74	Field accession	Itrana	2	ITA
ESP-046	1259	Field accession	Itrana	2	ITA
ESP-046	99	Field accession	Izmir Sofralik	3	TUR
ESP-046	1118	Field accession	Jabali	2	SYR
ESP-046	392	Field accession	Jabaluna	2	ESP
ESP-046	1718	Field accession	Jaén_1	3	ESP
ESP-046	1719	Field accession	Jaén_2	3	ESP

INIA-FAO collection code	Register Number of accessions	Type of material	Accesion name	No Trees/Plants	Origin*
ESP-046	1602	New accession at propagation facilities	Janude_1	3	SYR
ESP-046	1603	New accession at propagation facilities	Janude_2	2	SYR
ESP-046	23	Field accession	Jaropo	2	ESP
ESP-046	1869	Field accession	Jeddari Chaal	3	TUN
ESP-046	1510	Field accession	Jemri Bouchouka	1	TUN
ESP-046	1908	Field accession	Jemri Bouchouka	4	TUN
ESP-046	1732	Field accession	Jemri Dhokkar-1732	3	TUN
ESP-046	1907	Field accession	Jemri Dhokkar-1907	3	TUN
ESP-046	1004	Field accession	Jlot	3	SYR
ESP-046	1965	Field accession	Jlot-1965	3	LBN
ESP-046	841	Field accession	Jlot-841	1	SYR
ESP-046	976	Field accession	Jlot-976	3	SYR
ESP-046	667	Field accession	Joanenca	3	ESP
ESP-046	1414	Field accession	Juca	3	MEX
ESP-046	1972	Field accession	Kadeshon	3	ISR
ESP-046	140	Field accession	Kaissy	1	SYR
ESP-046	105	Field accession	Kalamon	3	GRC
ESP-046	2068	New accession at propagation facilities	Kalb el Tair	3	LBN
ESP-046	2335	New accession at propagation facilities	Kalembezi	1	TUR
ESP-046	1171	Field accession	Kalinjot	2	ALB
ESP-046	1151	Field accession	Kallmet	3	ALB
ESP-046	1712	Field accession	Kallmet Kokerrvogel	2	ALB
ESP-046	691	Field accession	Kalokerida	2	GRC
ESP-046	688	Field accession	Kan Celebi	2	TUR
ESP-046	2255	New accession at propagation facilities	Kanabisi	2	JOR
ESP-046	2365	New accession at propagation facilities	Kara Yaprak	3	TUR
ESP-046	1119	Field accession	Karamani	1	SYR
ESP-046	1564	New accession at propagation facilities	Karamani-1564	3	SYR
ESP-046	2326	New accession at propagation facilities	Karamani-2326	1	TUR
ESP-046	1986	Field accession	Karbunčela	3	HRV

INIA-FAO collection code	Register Number of accessions	Type of material	Accesion name	No Trees/Plants	Origin*
ESP-046	1606	New accession at propagation facilities	Karme	3	SYR
ESP-046	2208	New accession at propagation facilities	Karydolia	3	GRC
ESP-046	1959	Field accession	Kato Dris_1	3	CYP
ESP-046	848	Field accession	Kato Drys	2	CYP
ESP-046	1913	Field accession	Kbiret Louzir	4	TUN
ESP-046	144	Field accession	Kelb et Ter-144	2	SYR
ESP-046	145	Field accession	Kelb et Ter-145	1	SYR
ESP-046	1563	New accession at propagation facilities	Kerdi	2	SYR
ESP-046	2256	New accession at propagation facilities	Ketat	2	JOR
ESP-046	2275	New accession at propagation facilities	Kfari Beladi	1	JOR
ESP-046	1804	Field accession	Kfari Romi	1	JOR
ESP-046	1789	Field accession	Kfarzeta	1	SYR
ESP-046	2501	Field accession	Kfarzeta-2501	1	SYR
ESP-046	1548	Field accession	Khadraia	2	DZA
ESP-046	1054	Field accession	Khalkhali	3	SYR
ESP-046	1107	Field accession	Khalkhali-1107	3	SYR
ESP-046	1595	New accession at propagation facilities	Khalkhali-1595	3	SYR
ESP-046	789	Field accession	Khalkhali-789	2	SYR
ESP-046	1120	Field accession	Khashabi	2	SYR
ESP-046	1597	Field accession	Khashabi-1597	3	SYR
ESP-046	1895	Field accession	Khchinet Sig	3	TUN
ESP-046	1573	New accession at propagation facilities	Khello	3	SYR
ESP-046	1618	New accession at propagation facilities	Khnfse	3	SYR
ESP-046	1593	New accession at propagation facilities	Khodeiri	3	SYR
ESP-046	1613	New accession at propagation facilities	Khuokhe	3	SYR
ESP-046	2375	New accession at propagation facilities	Kilis yaglik	1	TUR
ESP-046	1571	New accession at propagation facilities	Killin	3	SYR
ESP-046	679	Field accession	Kiraz	2	TUR
ESP-046	849	Field accession	Klirou	2	CYP
ESP-046	1081	Field accession	Klon_14-1081	3	ALB

INIA-FAO collection code	Register Number of accessions	Type of material	Accesion name	No Trees/Plants	Origin*
ESP-046	1812	Field accession	Klon_14-1812	3	ALB
ESP-046	1716	Field accession	Kokerrmadh Elbasani	2	ALB
ESP-046	2267	New accession at propagation facilities	Kokerrmadhi i Beratit	2	ALB
ESP-046	702	Field accession	Kolybada	2	GRC
ESP-046	219	Field accession	Konservolia	4	GRC
ESP-046	1961	Field accession	Korakoy	3	CYP
ESP-046	218	Field accession	Koroneiki	3	GRC
ESP-046	1480	Field accession	Koroneiki	3	GRC
ESP-046	1754	Field accession	Kosiem	1	EGY
ESP-046	1981	Field accession	Kosmača	2	HRV
ESP-046	1385	Field accession	Kothreiki	3	GRC
ESP-046	1077	Field accession	Kotruvsi	2	ALB
ESP-046	2227	New accession at propagation facilities	Koutsourelia	1	GRC
ESP-046	1713	Field accession	Krypsi i Krujes	2	ALB
ESP-046	2320	New accession at propagation facilities	Kucuk Topak Ulak	1	TUR
ESP-046	1147	Field accession	Kusha-1147	2	ALB
ESP-046	2270	New accession at propagation facilities	Kusha-2270	3	ALB
ESP-046	2219	New accession at propagation facilities	La Bechude	3	FRA
ESP-046	2278	New accession at propagation facilities	Labeed	2	IRQ
ESP-046	2240	New accession at propagation facilities	Ladoelia	2	CYP
ESP-046	1962	Field accession	Lagoydhera	1	CYP
ESP-046	1205	Field accession	Larcianese	2	ITA
ESP-046	704	Field accession	Lastovka	3	HRV
ESP-046	1207	Field accession	Lazzera Reale	3	ITA
ESP-046	82	Field accession	Leccino	2	ITA
ESP-046	1208	Field accession	Leccino	3	ITA
ESP-046	1260	Field accession	Leccino	2	ITA
ESP-046	1838	Field accession	Leccino	1	ITA
ESP-046	1209	Field accession	Leccio del Corno	3	ITA
ESP-046	1210	Field accession	Leccio Maremmano	2	ITA

INIA-FAO collection code	Register Number of accessions	Type of material	Accesion name	No Trees/Plants	Origin*
ESP-046	1261	Field accession	Leccio Maremmano	2	ITA
ESP-046	1211	Field accession	Leccione	2	ITA
ESP-046	54	Field accession	Lechín de Granada	4	ESP
ESP-046	5	Field accession	Lechín de Sevilla	4	ESP
ESP-046	139	Field accession	Lechín de Sevilla	4	ESP
ESP-046	149	Field accession	Lechín de Sevilla	2	ESP
ESP-046	758	Field accession	Lemeño	3	ESP
ESP-046	384	Field accession	Lentisca	4	ESP
ESP-046	2018	Field accession	Leucocarpa	2	ITA
ESP-046	703	Field accession	Levantinka	2	HRV
ESP-046	108	Field accession	Lianolia Kerkyras	3	GRC
ESP-046	694	Field accession	Lianolia Kerkyras	2	GRC
ESP-046	1459	Field accession	Liguria	3	CHL
ESP-046	1537	Field accession	Limli	2	DZA
ESP-046	1949	Field accession	Limli	2	DZA
ESP-046	35	Field accession	Limoncillo	4	ESP
ESP-046	2386	New accession at propagation facilities	Ljubuški Stari Grad	1	BiH
ESP-046	2176	New accession at propagation facilities	Llimonenca	3	ESP
ESP-046	1369	Field accession	Lloma	3	ESP
ESP-046	534	Field accession	Llorón de Atarfe	2	ESP
ESP-046	400	Field accession	Llorón de Iznalloz	6	ESP
ESP-046	376	Field accession	Llorón de Ronda	2	ESP
ESP-046	226	Field accession	Llumeta	3	ESP
ESP-046	414	Field accession	Loaime	4	ESP
ESP-046	1534	Field accession	Longue de Meliana	2	DZA
ESP-046	753	Field accession	Lucio	4	ESP
ESP-046	763	Field accession	Lucio	1	ESP
ESP-046	322	Field accession	Lucques	6	FRA
ESP-046	1997	Field accession	Lumbardina	2	MNE
ESP-046	1337	Field accession	Lumiaro	2	ITA

INIA-FAO collection code	Register Number of accessions	Type of material	Accesion name	No Trees/Plants	Origin*
ESP-046	1125	Field accession	Maarri	2	SYR
ESP-046	28	Field accession	Macho de Jaén	2	ESP
ESP-046	448	Field accession	Machorrón	3	ESP
ESP-046	1212	Field accession	Madonna dell'Impruneta	3	ITA
ESP-046	130	Field accession	Madural	2	PRT
ESP-046	856	Field accession	Maelia	4	ISR
ESP-046	1010	Field accession	Mahati-1010	4	SYR
ESP-046	1581	Field accession	Mahati-1581	3	SYR
ESP-046	846	Field accession	Mahati-846	7	SYR
ESP-046	1262	Field accession	Maiatica di Ferrandina	2	ITA
ESP-046	1059	Field accession	Majhol-1059	4	SYR
ESP-046	1063	Field accession	Majhol-1063	3	SYR
ESP-046	1122	Field accession	Majhol-1122	2	SYR
ESP-046	152	Field accession	Majhol-152	3	SYR
ESP-046	1317	Field accession	Majorca	2	ITA
ESP-046	1711	Field accession	Managjel	2	ALB
ESP-046	1782	Field accession	Manakiri-1782	3	SYR
ESP-046	1600	New accession at propagation facilities	Manakiri	3	SYR
ESP-046	1681	Field accession	Mangar Erragma	2	TUN
ESP-046	1313	Field accession	Manna	2	ITA
ESP-046	489	Field accession	Manzanil de Piedra Buena-489	1	ESP
ESP-046	1451	Field accession	Manzanilla Aceitera	3	CHL
ESP-046	1504	Field accession	Manzanilla Aceitera	3	ARG
ESP-046	430	Field accession	Manzanilla Cacereña	2	ESP
ESP-046	1460	Field accession	Manzanilla Chilena	2	CHL
ESP-046	770	Field accession	Manzanilla de Abla	5	ESP
ESP-046	1492	Field accession	Manzanilla de Abla	1	ESP
ESP-046	406	Field accession	Manzanilla de Agua	2	ESP
ESP-046	760	Field accession	Manzanilla de Agua	2	ESP
ESP-046	772	Field accession	Manzanilla de Agua	2	ESP

INIA-FAO collection code	Register Number of accessions	Type of material	Accesion name	No Trees/Plants	Origin*
ESP-046	775	Field accession	Manzanilla de Agua	2	ESP
ESP-046	784	Field accession	Manzanilla de Almería	2	ESP
ESP-046	971	Field accession	Manzanilla de Ayora	4	ESP
ESP-046	2188	New accession at propagation facilities	Manzanilla de Caudiel	3	ESP
ESP-046	801	Field accession	Manzanilla de Hellín	2	ESP
ESP-046	56	Field accession	Manzanilla de Jaén	2	ESP
ESP-046	401	Field accession	Manzanilla de Montefrío	7	ESP
ESP-046	2178	New accession at propagation facilities	Manzanilla de Requena	3	ESP
ESP-046	1134	Field accession	Manzanilla de San Vicente	2	MEX
ESP-046	21	Field accession	Manzanilla de Sevilla	2	ESP
ESP-046	267	Field accession	Manzanilla de Sevilla	2	ESP
ESP-046	722	Field accession	Manzanilla de Sevilla	2	ESP
ESP-046	881	Field accession	Manzanilla de Sevilla	2	ESP
ESP-046	1172	Field accession	Manzanilla de Sevilla	3	ESP
ESP-046	22	Field accession	Manzanilla de Tortosa	2	ESP
ESP-046	499	Field accession	Manzanilla del Centro	3	ESP
ESP-046	269	Field accession	Manzanilla del Piquito	2	ESP
ESP-046	377	Field accession	Manzanilla Picúa	2	ESP
ESP-046	360	Field accession	Manzanilla Prieta	2	ESP
ESP-046	1129	Field accession	Manzanilla Valle las Palmeras	2	MEX
ESP-046	757	Field accession	Manzanillera de Huércal Overa	3	ESP
ESP-046	1818	Field accession	Manzanillo Cordobés de Cabra	1	ESP
ESP-046	1742	Field accession	Maraki	2	EGY
ESP-046	2292	New accession at propagation facilities	Marantelli	3	TUR
ESP-046	1496	Field accession	Marfil	1	ESP
ESP-046	1923	Field accession	Marfil	3	ESP
ESP-046	1143	Field accession	Mari	4	IRN
ESP-046	2266	New accession at propagation facilities	Marksi	1	ALB
ESP-046	535	Field accession	Marsaline	8	TUN
ESP-046	1509	Field accession	Marsaline-1509	5	TUN

INIA-FAO collection code	Register Number of accessions	Type of material	Accesion name	No Trees/Plants	Origin*
ESP-046	1684	Field accession	Marsaline-1684	6	TUN
ESP-046	299	Field accession	Marteño de la Carlota	2	ESP
ESP-046	1213	Field accession	Marzio	3	ITA
ESP-046	1121	Field accession	Masabi	4	SYR
ESP-046	1970	Field accession	Masepo	3	ISR
ESP-046	2069	New accession at propagation facilities	Masri	3	LBN
ESP-046	345	Field accession	Mastoidis	1	GRC
ESP-046	1388	Field accession	Mastoidis	2	GRC
ESP-046	2177	New accession at propagation facilities	Matias	3	ESP
ESP-046	2209	New accession at propagation facilities	Matolia	3	GRC
ESP-046	84	Field accession	Maurino	2	ITA
ESP-046	1214	Field accession	Maurino	2	ITA
ESP-046	1263	Field accession	Maurino	2	ITA
ESP-046	1389	Field accession	Mavrelia	2	GRC
ESP-046	699	Field accession	Mavreya	2	GRC
ESP-046	990	Field accession	Mawi	2	SYR
ESP-046	1013	Field accession	Mawi	2	SYR
ESP-046	138	Field accession	Mawi Stanboli	2	SYR
ESP-046	2236	New accession at propagation facilities	Megaron	3	GRC
ESP-046	1519	Field accession	Mekki	1	DZA
ESP-046	2349	New accession at propagation facilities	Melkabazi	3	TUR
ESP-046	1891	Field accession	Mellahi Kotti	4	TUN
ESP-046	1747	Field accession	Meloky	2	EGY
ESP-046	513	Field accession	Meloncillo	3	ESP
ESP-046	93	Field accession	Memecik	2	TUR
ESP-046	2359	New accession at propagation facilities	Memeli	3	TUR
ESP-046	836	Field accession	Menara	2	MAR
ESP-046	2224	New accession at propagation facilities	Menudel	3	FRA
ESP-046	669	Field accession	Menya	4	ESP
ESP-046	1071	Field accession	Menya	3	ESP

INIA-FAO collection code	Register Number of accessions	Type of material	Accesion name	No Trees/Plants	Origin*
ESP-046	120	Field accession	Merhavia	2	ISR
ESP-046	115	Field accession	Meski	2	TUN
ESP-046	1554	Field accession	Meski	1	TUN
ESP-046	1665	Field accession	Meski	3	TUN
ESP-046	1676	Field accession	Meski	1	TUN
ESP-046	1727	Field accession	Meski	3	TUN
ESP-046	1866	Field accession	Meski	2	TUN
ESP-046	1831	Field accession	Meski Zarzis	3	TUN
ESP-046	1905	Field accession	Meski Zarzis	2	TUN
ESP-046	1832	Field accession	Meskyet Dawla Gafsa	2	TUN
ESP-046	1885	Field accession	Meskyet Dawla Gafsa	2	TUN
ESP-046	1628	New accession at propagation facilities	Mesyaf_2	3	SYR
ESP-046	1887	Field accession	Mfartah Blettech	2	TUN
ESP-046	1215	Field accession	Mignolo	3	ITA
ESP-046	1216	Field accession	Mignolo Cerretano	3	ITA
ESP-046	1133	Field accession	Misión de San Vicente	1	MEX
ESP-046	708	Field accession	Mission Moojeski	2	USA
ESP-046	716	Field accession	Mission Nieland	1	USA
ESP-046	1079	Field accession	Mixani	2	ALB
ESP-046	1872	Field accession	Mlouki Blettech	3	TUN
ESP-046	2040	Field accession	Mochuto	3	ESP
ESP-046	847	Field accession	Mohazam Abou Satl	3	SYR
ESP-046	859	Field accession	Mohazan	2	ISR
ESP-046	864	Field accession	Mollar Basto	2	ESP
ESP-046	440	Field accession	Mollar de Cieza	2	ESP
ESP-046	450	Field accession	Mollar de Cieza	4	ESP
ESP-046	544	Field accession	Mollar de Cieza	4	ESP
ESP-046	612	Field accession	Mollar de Cieza	2	ESP
ESP-046	2231	New accession at propagation facilities	Moncita	3	FRA
ESP-046	78	Field accession	Moraiolo	2	ITA

INIA-FAO collection code	Register Number of accessions	Type of material	Accesion name	No Trees/Plants	Origin*
ESP-046	85	Field accession	Moraiolo	4	ITA
ESP-046	1217	Field accession	Moraiolo	3	ITA
ESP-046	1264	Field accession	Moraiolo	2	ITA
ESP-046	86	Field accession	Moraiolo T. Corsini	2	ITA
ESP-046	1218	Field accession	Morcaio	3	ITA
ESP-046	1220	Field accession	Morchiaio	3	ITA
ESP-046	1265	Field accession	Morchiaio	2	ITA
ESP-046	1219	Field accession	Morchione	3	ITA
ESP-046	1221	Field accession	Morcone	3	ITA
ESP-046	349	Field accession	Morejona	3	ESP
ESP-046	1340	Field accession	Moresca	2	ITA
ESP-046	17	Field accession	Morisca	2	ESP
ESP-046	740	Field accession	Morisca de Mancor	2	ESP
ESP-046	270	Field accession	Morona	2	ESP
ESP-046	368	Field accession	Morona	2	ESP
ESP-046	2198	New accession at propagation facilities	Morona de Castellón	3	ESP
ESP-046	2174	New accession at propagation facilities	Morruda de Salinas	3	ESP
ESP-046	2192	New accession at propagation facilities	Morruda de Segorbe	1	ESP
ESP-046	224	Field accession	Morrut	5	ESP
ESP-046	809	Field accession	Morrut	3	ESP
ESP-046	607	Field accession	Morrut-607	3	ESP
ESP-046	1588	New accession at propagation facilities	Mossabi	3	SYR
ESP-046	1991	Field accession	Mrčakinja	3	HRV
ESP-046	1611	New accession at propagation facilities	Munkar Kak	3	SYR
ESP-046	700	Field accession	Myrtolia	2	GRC
ESP-046	158	Field accession	Nabali	2	ISR
ESP-046	341	Field accession	Nabali	1	ISR
ESP-046	2260	New accession at propagation facilities	Nabali Baladi	2	JOR
ESP-046	2095	New accession at propagation facilities	Nabali Lari	2	LBN
ESP-046	2262	New accession at propagation facilities	Nabali Muhasan	2	JOR

INIA-FAO collection code	Register Number of accessions	Type of material	Accesion name	No Trees/Plants	Origin*
ESP-046	2035	Field accession	Nación	3	ESP
ESP-046	1341	Field accession	Nasitana	2	ITA
ESP-046	2263	New accession at propagation facilities	Nasouhi Jaba	2	JOR
ESP-046	857	Field accession	Nasuhi	2	ISR
ESP-046	1524	Field accession	Neb Djemel	2	DZA
ESP-046	1859	Field accession	Neb Tataouine	2	TUN
ESP-046	1902	Field accession	Neb Tataouine	2	TUN
ESP-046	2029	Field accession	Negral de Bierge	2	ESP
ESP-046	1075	Field accession	Negral de Sabiñán	2	ESP
ESP-046	27	Field accession	Negrillo de Arjona	2	ESP
ESP-046	794	Field accession	Negrillo de Arjona	2	ESP
ESP-046	301	Field accession	Negrillo de Estepa	2	ESP
ESP-046	411	Field accession	Negrillo de Iznalloz	2	ESP
ESP-046	355	Field accession	Negrillo de la Carlota	2	ESP
ESP-046	410	Field accession	Negrillo Redondo	2	ESP
ESP-046	123	Field accession	Negrinha	2	PRT
ESP-046	2161	Field accession	Negro de Aguilar	3	ESP
ESP-046	750	Field accession	Negro del Carpio	2	ESP
ESP-046	1314	Field accession	Nera di Gonnos	2	ITA
ESP-046	1308	Field accession	Nera di Oliena	5	ITA
ESP-046	2038	New accession at propagation facilities	Neral	1	ESP
ESP-046	2253	New accession at propagation facilities	Neral	3	ESP
ESP-046	1342	Field accession	Nerba	3	ITA
ESP-046	1343	Field accession	Nerba Catanese	3	ITA
ESP-046	44	Field accession	Nevadillo Blanco de Jaén	2	ESP
ESP-046	58	Field accession	Nevadillo Blanco de Lucena	2	ESP
ESP-046	351	Field accession	Nevadillo Blanco de Osuna	2	ESP
ESP-046	1132	Field accession	Nevadillo de San Vicente	2	MEX
ESP-046	335	Field accession	Nevadillo de Santisteban Pto	2	ESP
ESP-046	791	Field accession	Nevadillo de Santisteban Pto	2	ESP

INIA-FAO collection code	Register Number of accessions	Type of material	Accesion name	No Trees/Plants	Origin*
ESP-046	37	Field accession	Nevadillo Negro de Jaén	2	ESP
ESP-046	45	Field accession	Nevadillo Negro de Jaén	3	ESP
ESP-046	2282	New accession at propagation facilities	Nevadillo Negro-2282	3	ESP
ESP-046	1128	Field accession	Nevadillo Valle las Palmeras	2	MEX
ESP-046	306	Field accession	Nevado Azul	2	ESP
ESP-046	307	Field accession	Nevado Azul	2	ESP
ESP-046	305	Field accession	Nevado Basto	2	ESP
ESP-046	2165	New accession at propagation facilities	Nevado Basto de Zuheros	3	ESP
ESP-046	773	Field accession	Nevado Rizado	2	ESP
ESP-046	1267	Field accession	Nocellara del Belice	2	ITA
ESP-046	1344	Field accession	Nocellara del Belice	2	ITA
ESP-046	1266	Field accession	Nocellara Etnea	2	ITA
ESP-046	1268	Field accession	Nocellara Messinese	2	ITA
ESP-046	1346	Field accession	Nocellara Messinese	3	ITA
ESP-046	1269	Field accession	Nociara	2	ITA
ESP-046	2216	New accession at propagation facilities	Noirette de l´Herault	3	FRA
ESP-046	860	Field accession	Novo	3	ISR
ESP-046	706	Field accession	Oblica	3	HRV
ESP-046	210	Field accession	Oblonga	2	USA
ESP-046	282	Field accession	Ocal	3	ESP
ESP-046	327	Field accession	Ocal	2	ESP
ESP-046	378	Field accession	Ocal	4	ESP
ESP-046	525	Field accession	Ocal	3	ESP
ESP-046	427	Field accession	Ocal de Alburquerque	2	ESP
ESP-046	25	Field accession	Ocal-25	2	ESP
ESP-046	1270	Field accession	Ogliarola del Bradano	2	ITA
ESP-046	1271	Field accession	Ogliarola del Vulture	2	ITA
ESP-046	413	Field accession	Ojo de Liebre	3	ESP
ESP-046	878	Field accession	Ojua	2	ESP
ESP-046	2184	New accession at propagation facilities	Olaya	2	ESP

INIA-FAO collection code	Register Number of accessions	Type of material	Accesion name	No Trees/Plants	Origin*
ESP-046	1305	Field accession	Olieddu	2	ITA
ESP-046	2141	New accession at propagation facilities	Olivar de la Marina	1	ESP
ESP-046	1222	Field accession	Olivastra di Populonia	3	ITA
ESP-046	1223	Field accession	Olivastra Seggianese	4	ITA
ESP-046	1844	Field accession	Oliviere	4	FRA
ESP-046	1272	Field accession	Olivo da Mensa	2	ITA
ESP-046	1973	Field accession	Olivo de Aguamarga	1	ESP
ESP-046	2160	New accession at propagation facilities	Olivo de Galicia	3	ESP
ESP-046	797	Field accession	Olivo de Mancha Real	3	ESP
ESP-046	309	Field accession	Olivo de Maura	2	ESP
ESP-046	2151	New accession at propagation facilities	Olivo de Nueva Carteya	3	ESP
ESP-046	1224	Field accession	Olivo del Mulino	3	ITA
ESP-046	1225	Field accession	Olivo di Casavecchia	3	ITA
ESP-046	1347	Field accession	Olivo di Castiglione	2	ITA
ESP-046	1348	Field accession	Olivo di Mandanici	2	ITA
ESP-046	1226	Field accession	Olivo di San Lorenzo	3	ITA
ESP-046	338	Field accession	Olivo Macho de Santisteban Pto	3	ESP
ESP-046	2250	New accession at propagation facilities	Olivonero de Ayerbe	2	ESP
ESP-046	2144	New accession at propagation facilities	Olivos de Cotillas	3	ESP
ESP-046	2148	New accession at propagation facilities	Olivos de El Barranco del Lobo	3	ESP
ESP-046	1622	New accession at propagation facilities	Om Kanane	2	SYR
ESP-046	1227	Field accession	Ornellaia	3	ITA
ESP-046	1930	Field accession	OT2	2	MAR
ESP-046	2197	New accession at propagation facilities	Otos_1	3	ESP
ESP-046	1735	Field accession	Oueslati T-1735	2	TUN
ESP-046	1919	Field accession	Oueslati T-1919	2	TUN
ESP-046	114	Field accession	Ouslati	2	TUN
ESP-046	1931	Field accession	OZ1	3	MAR
ESP-046	2390	New accession at propagation facilities	Pačica	1	HRV
ESP-046	771	Field accession	Pajarera	2	ESP

INIA-FAO collection code	Register Number of accessions	Type of material	Accesion name	No Trees/Plants	Origin*
ESP-046	26	Field accession	Pajarero	2	ESP
ESP-046	1484	Field accession	Pajarero	3	ESP
ESP-046	1307	Field accession	Palma	2	ITA
ESP-046	1273	Field accession	Palmarola	2	ITA
ESP-046	661	Field accession	Palomar	2	ESP
ESP-046	369	Field accession	Palomillo	2	ESP
ESP-046	2244	New accession at propagation facilities	Panseñera	1	ESP
ESP-046	2220	New accession at propagation facilities	Pardiguier	3	FRA
ESP-046	1309	Field accession	Paschixedda	3	ITA
ESP-046	2193	New accession at propagation facilities	Pascual_1	2	ESP
ESP-046	1349	Field accession	Passulunara	2	ITA
ESP-046	1445	Field accession	Patrón de Cabús	1	ESP
ESP-046	649	Field accession	Patronet	2	ESP
ESP-046	272	Field accession	Pavo	2	ESP
ESP-046	1462	Field accession	Pavo	1	ESP
ESP-046	421	Field accession	Pecoso	4	ESP
ESP-046	87	Field accession	Pendolino	4	ITA
ESP-046	1274	Field accession	Pendolino	2	ITA
ESP-046	1136	Field accession	Pendolino-1136	3	PER
ESP-046	1228	Field accession	Pendolino-1228	3	ITA
ESP-046	1442	Field accession	Penjoll	2	ESP
ESP-046	562	Field accession	Pequeña de Casas Ibáñez	4	ESP
ESP-046	1073	Field accession	Perafort	2	ESP
ESP-046	1229	Field accession	Pesciatino	3	ITA
ESP-046	2217	New accession at propagation facilities	Petit Ribier	3	FRA
ESP-046	1230	Field accession	Piangente	3	ITA
ESP-046	1505	Field accession	Piangente	2	ARG
ESP-046	70	Field accession	Picholine	2	FRA
ESP-046	1275	Field accession	Picholine	2	FRA
ESP-046	1471	Field accession	Picholine	5	FRA

INIA-FAO collection code	Register Number of accessions	Type of material	Accesion name	No Trees/Plants	Origin*
ESP-046	101	Field accession	Picholine Marocaine	4	MAR
ESP-046	1091	Field accession	Picholine-1091	2	FRA
ESP-046	273	Field accession	Pico Limón	2	ESP
ESP-046	529	Field accession	Pico Limón	4	ESP
ESP-046	371	Field accession	Pico Limón de Grazalema	4	ESP
ESP-046	2136	New accession at propagation facilities	Picoso	3	ESP
ESP-046	9	Field accession	Picual	6	ESP
ESP-046	287	Field accession	Picual	2	ESP
ESP-046	662	Field accession	Picual	2	ESP
ESP-046	808	Field accession	Picual	3	ESP
ESP-046	1470	Field accession	Picual	5	ESP
ESP-046	798	Field accession	Picual de Almería	2	ESP
ESP-046	354	Field accession	Picual de Estepa	2	ESP
ESP-046	312	Field accession	Picual de Hoja Clara	1	ESP
ESP-046	313	Field accession	Picual de Hoja Oscura	1	ESP
ESP-046	2185	New accession at propagation facilities	Picuda de Luis	3	ESP
ESP-046	3	Field accession	Picudo	2	ESP
ESP-046	276	Field accession	Picudo Blanco de Estepa	1	ESP
ESP-046	316	Field accession	Picudo de Fruto Rojo	2	ESP
ESP-046	2201	New accession at propagation facilities	Picudo de la Axerquía	2	ESP
ESP-046	2156	New accession at propagation facilities	Picudo de la Rioja	2	ESP
ESP-046	2251	New accession at propagation facilities	Picudo de Labata	3	ESP
ESP-046	748	Field accession	Picudo de Montoro	4	ESP
ESP-046	1350	Field accession	Pidicuddara	2	ITA
ESP-046	2247	New accession at propagation facilities	Piga	3	ESP
ESP-046	1392	Field accession	Pikrolia	3	GRC
ESP-046	969	Field accession	Piñonera	2	ESP
ESP-046	1322	Field accession	Pizz'e Carroga	3	ITA
ESP-046	1351	Field accession	Pizzo di Corvo	2	ITA
ESP-046	705	Field accession	Plementa Bjelica	5	HRV

INIA-FAO collection code	Register Number of accessions	Type of material	Accesion name	No Trees/Plants	Origin*
ESP-046	1967	Field accession	PM-M16	1	MAR
ESP-046	1968	Field accession	PM-S8	3	MAR
ESP-046	1130	Field accession	Polinizador	2	MEX
ESP-046	1085	Field accession	Pulazeqin	2	ALB
ESP-046	1231	Field accession	Punteruolo	3	ITA
ESP-046	1232	Field accession	Puntino	3	ITA
ESP-046	1974	Field accession	Puntoža	3	HRV
ESP-046	2187	New accession at propagation facilities	Queixal de Porc	3	ESP
ESP-046	693	Field accession	Rachati	4	GRC
ESP-046	1393	Field accession	Rachati I.Y.E.X	3	GRC
ESP-046	418	Field accession	Racimal	6	ESP
ESP-046	34	Field accession	Racimal de Jaén	2	ESP
ESP-046	2195	New accession at propagation facilities	Racimo	3	ESP
ESP-046	2032	Field accession	Rañinera	3	ESP
ESP-046	277	Field accession	Rapasayo	2	ESP
ESP-046	1799	Field accession	Rasei	3	JOR
ESP-046	1276	Field accession	Ravece	2	ITA
ESP-046	1233	Field accession	Razzaio	2	ITA
ESP-046	1234	Field accession	Razzo	3	ITA
ESP-046	177	Field accession	Razzola	4	ITA
ESP-046	49	Field accession	Real Sevillana	2	ESP
ESP-046	372	Field accession	Rechino	3	ESP
ESP-046	127	Field accession	Redondil	2	PRT
ESP-046	876	Field accession	Redondil	3	ESP
ESP-046	365	Field accession	Redondilla de Grazalema	2	ESP
ESP-046	16	Field accession	Redondilla de Logroño	4	ESP
ESP-046	1161	Field accession	Redondilla de Logroño	1	ESP
ESP-046	966	Field accession	Reixonenca	2	ESP
ESP-046	1762	Field accession	Remmani-1762	4	LBN
ESP-046	2081	New accession at propagation facilities	Remmani-2081	3	LBN

INIA-FAO collection code	Register Number of accessions	Type of material	Accesion name	No Trees/Plants	Origin*
ESP-046	2388	New accession at propagation facilities	Resulja	1	HRV
ESP-046	1671	Field accession	R'Khami	1	TUN
ESP-046	1864	Field accession	R'Khami	4	TUN
ESP-046	2024	Field accession	Roalejo	2	ESP
ESP-046	2173	New accession at propagation facilities	Rogeta de Gorga_1	3	ESP
ESP-046	1448	Field accession	Rojal	2	ESP
ESP-046	2183	New accession at propagation facilities	Rojal de Valencia	3	ESP
ESP-046	1944	Field accession	Ronde de la Ménara	3	MAR
ESP-046	1533	Field accession	Ronde de Meliana	2	DZA
ESP-046	1809	Field accession	Rosai-1809	4	JOR
ESP-046	2254	New accession at propagation facilities	Rosai-2254	3	JOR
ESP-046	88	Field accession	Rosciola	4	ITA
ESP-046	1235	Field accession	Rosino	2	ITA
ESP-046	1236	Field accession	Rossellino	1	ITA
ESP-046	1237	Field accession	Rossello	2	ITA
ESP-046	1977	Field accession	Rosulja	3	HRV
ESP-046	1277	Field accession	Rotondella	2	ITA
ESP-046	2204	New accession at propagation facilities	Rougette de l'Ardèche	3	FRA
ESP-046	1536	Field accession	Rougette de Metidja	1	DZA
ESP-046	2500	Field accession	Rougette de Metidja-2500	1	DZA
ESP-046	2215	New accession at propagation facilities	Rougette de Pignans	3	FRA
ESP-046	1770	Field accession	Roumani (Qana)	2	LBN
ESP-046	1139	Field accession	Rowghani	6	IRN
ESP-046	1182	Field accession	Royal	3	ESP
ESP-046	1186	Field accession	Royal de Belchite	3	ESP
ESP-046	965	Field accession	Royal de Calatayud	2	ESP
ESP-046	47	Field accession	Royal de Calatayud-47	2	ESP
ESP-046	390	Field accession	Royal de Cazorla	3	ESP
ESP-046	962	Field accession	Royal de Sabiñán	2	ESP
ESP-046	806	Field accession	Royal de Villena	4	ESP

INIA-FAO collection code	Register Number of accessions	Type of material	Accesion name	No Trees/Plants	Origin*
ESP-046	2252	New accession at propagation facilities	Royeta de Abizanda	3	ESP
ESP-046	2030	Field accession	Royeta de Asque	2	ESP
ESP-046	2159	Field accession	Royuela	3	ESP
ESP-046	2182	New accession at propagation facilities	Rufina	3	ESP
ESP-046	717	Field accession	S. George Greys	4	USA
ESP-046	2047	New accession at propagation facilities	S:27_4	3	PER
ESP-046	665	Field accession	Sabatera	3	ESP
ESP-046	2070	New accession at propagation facilities	Sabbighi	3	LBN
ESP-046	2297	New accession at propagation facilities	Sacakli otur	3	TUR
ESP-046	142	Field accession	Safrawi	2	SYR
ESP-046	1112	Field accession	Safrawi	4	SYR
ESP-046	1117	Field accession	Safrawi	1	SYR
ESP-046	1116	Field accession	Safrawi-1116	2	SYR
ESP-046	1574	Field accession	Safrawi-1574	2	SYR
ESP-046	1562	Field accession	Saifi	3	SYR
ESP-046	348	Field accession	Salgar Redondo	2	ESP
ESP-046	1241	Field accession	Salicino	2	ITA
ESP-046	73	Field accession	Salonenque	2	FRA
ESP-046	2315	New accession at propagation facilities	Sam (Iznik)	3	TUR
ESP-046	2316	New accession at propagation facilities	Samanli	3	TUR
ESP-046	2294	New accession at propagation facilities	Samsun Kirmizi Tuzlamalik	1	TUR
ESP-046	2306	New accession at propagation facilities	Samsun Salamuralik	3	TUR
ESP-046	2351	New accession at propagation facilities	Samsun Tuzlamalik	3	TUR
ESP-046	684	Field accession	Samsun Ufak Tuzlamalik	4	TUR
ESP-046	2301	New accession at propagation facilities	Samsun Ufak Tuzlamalik	1	TUR
ESP-046	2290	New accession at propagation facilities	Samsun Yaglik	3	TUR
ESP-046	997	Field accession	San Pedro	2	CHL
ESP-046	956	Field accession	Sandalio	2	ESP
ESP-046	71	Field accession	Sant' Agostino	2	ITA
ESP-046	1278	Field accession	Sant' Agostino	2	ITA

INIA-FAO collection code	Register Number of accessions	Type of material	Accesion name	No Trees/Plants	Origin*
ESP-046	1239	Field accession	San Francesco	2	ITA
ESP-046	1279	Field accession	San Francesco-1279	2	ITA
ESP-046	1240	Field accession	San Lazzero	2	ITA
ESP-046	72	Field accession	Santa Caterina	1	ITA
ESP-046	1238	Field accession	Santa Caterina	2	ITA
ESP-046	1280	Field accession	Santa Martinenga	2	ITA
ESP-046	2323	New accession at propagation facilities	Sari Habesi (Hatay)	1	TUR
ESP-046	2319	New accession at propagation facilities	Sari Ulak	3	TUR
ESP-046	1306	Field accession	Sassarese	3	ITA
ESP-046	2300	New accession at propagation facilities	Sati	3	TUR
ESP-046	2324	New accession at propagation facilities	Saurani	1	TUR
ESP-046	2218	New accession at propagation facilities	Sauzin Vert	3	FRA
ESP-046	1670	Field accession	Sayali	3	TUN
ESP-046	1677	Field accession	Sayali T-1677	3	TUN
ESP-046	1728	Field accession	Sayali T-1728	2	TUN
ESP-046	1875	Field accession	Sayali T-1875	3	TUN
ESP-046	1863	Field accession	Sayali-1863	3	TUN
ESP-046	1056	Field accession	Sayfi	2	SYR
ESP-046	2325	New accession at propagation facilities	Sayfi-2325	1	TUR
ESP-046	1749	Field accession	Sebhawy	1	EGY
ESP-046	893	Field accession	Selvatico	1	ITA
ESP-046	1323	Field accession	Semidana	2	ITA
ESP-046	1876	Field accession	Semni Djebeniana	2	TUN
ESP-046	2169	New accession at propagation facilities	Sennara	2	EGY
ESP-046	1971	Field accession	Sepoka	3	ISR
ESP-046	2179	New accession at propagation facilities	Sepriana	2	ESP
ESP-046	1483	Field accession	Sevillana	3	ESP
ESP-046	1452	Field accession	Sevillana (Azapa)	3	CHL
ESP-046	1454	Field accession	Sevillana (Tacna)	2	CHL
ESP-046	768	Field accession	Sevillana de Abla	2	ESP

INIA-FAO collection code	Register Number of accessions	Type of material	Accesion name	No Trees/Plants	Origin*
ESP-046	593	Field accession	Sevillano de Jumilla	1	ESP
ESP-046	227	Field accession	Sevillenca	2	ESP
ESP-046	608	Field accession	Sevillenca	2	ESP
ESP-046	1786	Field accession	Shame	4	SYR
ESP-046	1620	New accession at propagation facilities	Shame-1620	3	SYR
ESP-046	1568	Field accession	Shami Modabl	3	SYR
ESP-046	1041	Field accession	Shami-1041	1	SYR
ESP-046	141	Field accession	Shami-141	2	SYR
ESP-046	2259	New accession at propagation facilities	Shami-2259	2	JOR
ESP-046	953	Field accession	Shami-953	2	SYR
ESP-046	1142	Field accession	Shengeh Manjil	2	IRN
ESP-046	1855	Field accession	Shengeh Manjil	2	IRN
ESP-046	1856	Field accession	Shengeh Sur	2	IRN
ESP-046	119	Field accession	Sigoise	4	DZA
ESP-046	1515	Field accession	Sigoise	4	DZA
ESP-046	1979	Field accession	Simjaca	1	HRV
ESP-046	1750	Field accession	Sinawy	1	EGY
ESP-046	685	Field accession	Sinop	5	TUR
ESP-046	2305	New accession at propagation facilities	Sinop no 1	1	TUR
ESP-046	2299	New accession at propagation facilities	Sinop no 2	1	TUR
ESP-046	2298	New accession at propagation facilities	Sinop no 5	3	TUR
ESP-046	2304	New accession at propagation facilities	Sinop no 6	3	TUR
ESP-046	1993	Field accession	Sitncica	3	MNE
ESP-046	1989	Field accession	Sitnica	3	HRV
ESP-046	1318	Field accession	Sivigliana da Mensa	2	ITA
ESP-046	1304	Field accession	Sivigliana da Olio	2	ITA
ESP-046	2167	Field accession	Siwi	2	EGY
ESP-046	2303	New accession at propagation facilities	Siyah Salamuralik (Tekirdag)	1	TUR
ESP-046	2237	New accession at propagation facilities	Smertolia	3	GRC
ESP-046	1925	Field accession	Solimar	3	ESP

INIA-FAO collection code	Register Number of accessions	Type of material	Accesion name	No Trees/Plants	Origin*
ESP-046	871	Field accession	Sollana	2	ESP
ESP-046	787	Field accession	Sorani	3	SYR
ESP-046	1566	New accession at propagation facilities	Sorani-1566	2	SYR
ESP-046	1918	Field accession	Souabaa Aljia	3	TUN
ESP-046	1513	Field accession	Souidi	1	DZA
ESP-046	1478	Field accession	Sourani-1478	2	SYR
ESP-046	1165	Field accession	Souri	3	ISR
ESP-046	1589	New accession at propagation facilities	Souri-1589	3	SYR
ESP-046	1843	Field accession	Souri-1843	4	ISR
ESP-046	1766	Field accession	Soury Lari	3	LBN
ESP-046	991	Field accession	Stanboli-991	2	SYR
ESP-046	1590	Field accession	Stanbouli	3	SYR
ESP-046	1999	New accession at propagation facilities	Stara Maslina-1999	1	MNE
ESP-046	2005	Field accession	Stara Maslina-2005	3	HRV
ESP-046	2273	New accession at propagation facilities	Štorta	3	SVN
ESP-046	2385	New accession at propagation facilities	Studenci	1	BiH
ESP-046	996	Field accession	Suca	2	CHL
ESP-046	1790	Field accession	Sukare	4	SYR
ESP-046	2394	New accession at propagation facilities	Tachuno	3	ESP
ESP-046	1947	Field accession	Takesrit	2	DZA
ESP-046	2058	Field accession	Tarabelsi	3	SYR
ESP-046	2369	New accession at propagation facilities	Tasarasi (Aydin)	2	TUR
ESP-046	2370	New accession at propagation facilities	Tasarasi (Kusadasi)	1	TUR
ESP-046	1794	Field accession	Tebabs	4	SYR
ESP-046	1777	Field accession	Tebabs	2	SYR
ESP-046	1539	Field accession	Tefah	2	DZA
ESP-046	2308	New accession at propagation facilities	Tekirdag cizmelik	2	TUR
ESP-046	2175	New accession at propagation facilities	Temprana de Montán	3	ESP
ESP-046	2191	New accession at propagation facilities	Tempranillo de Ayora	3	ESP
ESP-046	756	Field accession	Tempranillo de Lucena	2	ESP

INIA-FAO collection code	Register Number of accessions	Type of material	Accesion name	No Trees/Plants	Origin*
ESP-046	1160	Field accession	Tempranillo de Lucena	1	ESP
ESP-046	1486	Field accession	Tempranillo de Lucena	3	ESP
ESP-046	561	Field accession	Tempranillo de Yeste	3	ESP
ESP-046	358	Field accession	Temprano	1	ESP
ESP-046	2362	New accession at propagation facilities	Ters Yaprak	2	TUR
ESP-046	1310	Field accession	Terza Grande	2	ITA
ESP-046	1311	Field accession	Terza Piccola	3	ITA
ESP-046	2339	New accession at propagation facilities	Tesbih Celebi	1	TUR
ESP-046	2100	New accession at propagation facilities	Teta de la Vaca	1	ESP
ESP-046	2239	New accession at propagation facilities	Thassitiki	3	GRC
ESP-046	1394	Field accession	Thiaki	3	GRC
ESP-046	2211	New accession at propagation facilities	Throubolia	1	GRC
ESP-046	2194	New accession at propagation facilities	Tío Blas	3	ESP
ESP-046	721	Field accession	Toffahi	4	EGY
ESP-046	1743	Field accession	Toffahi	1	EGY
ESP-046	2056	Field accession	Toffahi	1	EGY
ESP-046	1000	Field accession	Toffahi-1000	2	SYR
ESP-046	1508	Field accession	Toffehi Tataouine	1	TUN
ESP-046	1683	Field accession	Toffehi Tataouine	3	TUN
ESP-046	1729	Field accession	Toffehi Tataouine	1	TUN
ESP-046	1903	Field accession	Toffehi Tataouine	2	TUN
ESP-046	2052	New accession at propagation facilities	Tomatillo	2	ESP
ESP-046	1315	Field accession	Tonda di Cagliari	3	ITA
ESP-046	1312	Field accession	Tonda di Villacidro	3	ITA
ESP-046	1352	Field accession	Tonda Iblea	4	ITA
ESP-046	1242	Field accession	Tondello	2	ITA
ESP-046	2162	New accession at propagation facilities	Torcío	3	ESP
ESP-046	767	Field accession	Torcío de Cabra	2	ESP
ESP-046	357	Field accession	Torcío de Huelma	3	ESP
ESP-046	774	Field accession	Toruno	2	ESP

INIA-FAO collection code	Register Number of accessions	Type of material	Accesion name	No Trees/Plants	Origin*
ESP-046	1629	Field accession	Tounsi Gafsa	1	TUN
ESP-046	1674	Field accession	Tounsi Gafsa	2	TUN
ESP-046	1883	Field accession	Tounsi Gafsa	4	TUN
ESP-046	2212	New accession at propagation facilities	Tragolia	3	GRC
ESP-046	992	Field accession	Trylia	3	SYR
ESP-046	1617	New accession at propagation facilities	Tufahi Tadmori	3	SYR
ESP-046	2232	New accession at propagation facilities	Ubac	3	FRA
ESP-046	1149	Field accession	Ulliri i Bardhe Berat	2	ALB
ESP-046	1082	Field accession	Ulliri i Bardhe i Tiranes	1	ALB
ESP-046	2499	Field accession	Ulliri i Bardhe i Tiranes-2499	1	ALB
ESP-046	1715	Field accession	Ulliri i Bardhe Kruje	2	ALB
ESP-046	1083	Field accession	Ulliri i Holle i Himares	2	ALB
ESP-046	1078	Field accession	Ulliri i Kuq	2	ALB
ESP-046	2384	New accession at propagation facilities	Ulliri i Zi	1	ALB
ESP-046	2268	New accession at propagation facilities	Ulliri Shekullor Berat	3	ALB
ESP-046	1714	Field accession	Unafka	2	ALB
ESP-046	110	Field accession	Unknown	2	PAK
ESP-046	75	Field accession	Uovo di Piccione	2	ITA
ESP-046	1282	Field accession	Uovo di Piccione	2	ITA
ESP-046	1464	Field accession	Uovo di Piccione	3	ITA
ESP-046	95	Field accession	Uslu	1	TUR
ESP-046	1353	Field accession	Vaddarica	3	ITA
ESP-046	103	Field accession	Valanolia	2	GRC
ESP-046	1837	Field accession	Valanolia	1	GRC
ESP-046	2020	Field accession	Valentins	3	ESP
ESP-046	613	Field accession	Vallesa	2	ESP
ESP-046	664	Field accession	Vaneta	3	ESP
ESP-046	749	Field accession	Varudo	3	ESP
ESP-046	850	Field accession	Varudo	2	ESP
ESP-046	1978	Field accession	Velika Lastovka	3	HRV

INIA-FAO collection code	Register Number of accessions	Type of material	Accesion name	No Trees/Plants	Origin*
ESP-046	660	Field accession	Vera	2	ESP
ESP-046	532	Field accession	Verdal de Alhama	2	ESP
ESP-046	663	Field accession	Verdal de Manresa	1	ESP
ESP-046	394	Field accession	Verdala	4	ESP
ESP-046	76	Field accession	Verdale	4	FRA
ESP-046	585	Field accession	Verdalón	2	ESP
ESP-046	887	Field accession	Verde Verdelho	3	PRT
ESP-046	2028	Field accession	Verdeña	3	ESP
ESP-046	877	Field accession	Verdial de Badajoz	4	ESP
ESP-046	889	Field accession	Verdial de Badajoz	2	ESP
ESP-046	988	Field accession	Verdial de Badajoz	2	ESP
ESP-046	1162	Field accession	Verdial de Badajoz	2	ESP
ESP-046	759	Field accession	Verdial de Badajoz	2	ESP
ESP-046	370	Field accession	Verdial de Cádiz	3	ESP
ESP-046	6	Field accession	Verdial de Huévar	2	ESP
ESP-046	155	Field accession	Verdial de Huévar	2	ESP
ESP-046	156	Field accession	Verdial de Huévar	2	ESP
ESP-046	954	Field accession	Verdial de Huévar	1	ESP
ESP-046	397	Field accession	Verdial de Montefrío	2	ESP
ESP-046	883	Field accession	Verdial de Vélez-Málaga	2	ESP
ESP-046	51	Field accession	Verdial de Vélez-Málaga-51	2	ESP
ESP-046	677	Field accession	Verdiell	3	ESP
ESP-046	36	Field accession	Verdilla de Calatayud	2	ESP
ESP-046	2132	New accession at propagation facilities	Vidrillo	3	ESP
ESP-046	364	Field accession	Villalonga	2	ESP
ESP-046	793	Field accession	Villalonga	2	ESP
ESP-046	1065	Field accession	Vinyols	2	ESP
ESP-046	1932	Field accession	VS6	2	MAR
ESP-046	725	Field accession	Wardan	2	EGY
ESP-046	1741	Field accession	Wateken	2	EGY

INIA-FAO collection code	Register Number of accessions	Type of material	Accesion name	No Trees/Plants	Origin*
ESP-046	2344	New accession at propagation facilities	Yag Celebi (Tatayn)	3	TUR
ESP-046	2366	New accession at propagation facilities	Yag Zeytini	2	TUR
ESP-046	2336	New accession at propagation facilities	Yaglik Celebi	2	TUR
ESP-046	2328	New accession at propagation facilities	Yaglik Sari Zeytin	1	TUR
ESP-046	2367	New accession at propagation facilities	Yerli Yaglik	2	TUR
ESP-046	689	Field accession	Yun Celebi	4	TUR
ESP-046	2341	New accession at propagation facilities	Yuvarlak Celebi	1	TUR
ESP-046	2334	New accession at propagation facilities	Yuvarlak Halhali	2	TUR
ESP-046	1982	Field accession	Žabarka	3	HRV
ESP-046	1607	New accession at propagation facilities	Zada	2	SYR
ESP-046	1605	Field accession	Zael Almuhra	3	SYR
ESP-046	788	Field accession	Zaity	2	SYR
ESP-046	1108	Field accession	Zaity	2	SYR
ESP-046	1109	Field accession	Zaity	1	SYR
ESP-046	117	Field accession	Zalmati	2	TUN
ESP-046	1679	Field accession	Zalmati Zarzis	1	TUN
ESP-046	1886	Field accession	Zalmati Zarzis	2	TUN
ESP-046	1888	Field accession	Zarbout Louzir	4	TUN
ESP-046	1140	Field accession	Zard	2	IRN
ESP-046	1810	Field accession	Zard-1810	1	IRN
ESP-046	1669	Field accession	Zarrazi	2	TUN
ESP-046	1828	Field accession	Zarrazi Douirat	3	TUN
ESP-046	1733	Field accession	Zarrazi Injassi	2	TUN
ESP-046	1911	Field accession	Zarrazi Injassi	1	TUN
ESP-046	1407	Field accession	Zarrazi Zarzis	2	TUN
ESP-046	1630	Field accession	Zarrazi Zarzis	1	TUN
ESP-046	1680	Field accession	Zarrazi Zarzis	4	TUN
ESP-046	1731	Field accession	Zarrazi Zarzis	2	TUN
ESP-046	1897	Field accession	Zarrazi Zarzis	4	TUN
ESP-046	1877	Field accession	Zarrazyet Sned	4	TUN

INIA-FAO collection code	Register Number of accessions	Type of material	Accesion name	No Trees/Plants	Origin*
ESP-046	38	Field accession	Zarza	2	ESP
ESP-046	356	Field accession	Zarzariega de Orcera	4	ESP
ESP-046	1933	Field accession	ZDH4	1	MAR
ESP-046	1934	Field accession	ZDH6	3	MAR
ESP-046	1948	Field accession	Zeboudj Boudoudan	3	DZA
ESP-046	1890	Field accession	Zeitoun Boubezzoula	3	TUN
ESP-046	1941	Field accession	Zeitun lkhl	2	MAR
ESP-046	1523	Field accession	Zeletni	2	DZA
ESP-046	1945	Field accession	Zitoune DK	3	MAR
ESP-046	1935	Field accession	Zms_1	3	MAR
ESP-046	2345	New accession at propagation facilities	Zoncuk	3	TUR
ESP-046	2134	New accession at propagation facilities	Zorzaleño	3	ESP
ESP-046	1936	Field accession	Zsb2	1	MAR
ESP-046	1996	Field accession	Žutica	2	MNE
ESP-046	1985	Field accession	Zuzorka	3	HRV
ESP-046	1946	Field accession	ZZ_10	2	MAR

*ALB=Albania ; ARG=Argentina ; BiH=Bosnia and Herzegovina ; CYP=Cyprus ; CHL=Chile ; DZA=Algeria ; EGY=Egypt ; ESP=Spain ; FRA=France ; GRC=Greece ; HRV=Croatia ; IRN=Iran ; IRQ=Iraq ; ISR=Israel ; ITA=Italy ; JOR=Jordan ; LBN=Lebanon ; MAR=Morocco ; MEX=Mexico ; MNE=Montenegro ; PAK=Pakistan ; PER=Peru ; PRT=Portugal ; SVN=Slovenia ; SYR=Syria ; TUN=Tunisia ; TUR=Turkey ; URU=Uruguay ; USA=United States of America
