# Supplementary material for: Utility of EST-SNP Markers for Improving Management and Use of Olive Genetic Resources: A Case Study at the Worldwide Olive Germplasm Bank of Córdoba
Source: Plants (Basel). 2022 Mar 29;11(7):921. doi: 10.3390/plants11070921 (PMC9002360; doi:10.3390/plants11070921)
Supplement: Supplementary file 1 [file plants-11-00921-s001.zip › Table S2.pdf]

Supplemental Table S2: Sequences of the core set of 96 EST-SNP markers used in the study

Contig	Sequence
EContig10374	CGAAGATGACAAGATCGATGAATTGGACAAGTCGGAGTACGTGGGAACTTTTGCACAAGTACCCCATAGTCATAAAGGACCTAAAAAGGT[A/G]AAGACGTCGATTAGGCTGGGATTAACAGAGCTTTTGGAGGATTTGGATGTAGAAGATGATGATTCAATCTTAGTGAGTTTGGTGCCACGGGCTGGAGATGTGACC
EContig10531	AAAGCAAGATTTCTGTCAAGAAAGAGGCAGTAAAGTCACAAACTGATGAAAAAGGCAATGCTAAATCAGATTCTGGTGCAAACTTTGACA[C/G]CTTTAAAGAAAACAGATCATCTGATAGCACTGATTATTCAAGTGCAATCTTGAGTGAAGAAATCAACAATAGCCCGACTGCAGCTATTTCATCGTCGACCGAGAA
EContig10539	GCCCTTCAGCACCCCTTTTCTTGACGATATAATTTCGTGAAGAATAGTTGGAATCAAACTTTTATGCAACTGATCTCTGTCGATGGGTGC[A/G]CGCTGTTTCACGTGCTCAATCACAGTAAATACTACTGTCTCGAAATCCAAGGGAAAGATGCATTTATTATATAGCAGAGGCCAGTTTGCACACTGGTTTTATGTA
EContig10640	TGCTTTCAATCACACTGCTTACAAGGAGGTGTTGGAAGCTGAACCTCCTGAGTGGCTTCCAGACAGTTCCACTACAGTTTGTATGCATTG[T/C]AATGCTCCTTTCACAGCTTTGACTCGTGGAAGACATCACTGCCGGTTTTGTGGAGGTATTTTTTGCAGGCTCTGCTCAAAAGGAAGATGCTTATTACCTTTTAAG
EContig10787_1	GTAATACCCCATCAGTGCCTCTGGTGTTTCGAGTCACAAGAATCTCTTCACTGGTGATTTCTCTGTAGATCGGTGGATTTTCTTCGGTCA[A/G]TAACTCCTTTATTGGTCCAAACACTTTATTTTTACTTTTTTCGTGATAGTGCAATCTGAAAAAACTTGCCACTGAGATTCTCGGCCCCACAGTTTTCGCCAATAC
EContig1180_2	TGCTGCTGCACGTTGAGGCTCCTCGGCGCATTCGCCATTTCTGCTCCTYGACGCCAGCTGAGAAGGAACGCAACCTAAAGGTCCAGTTCC[A/G]GTGACAAGAACCCTTCTGGTTCCCAATTCATGTAGCCTCATCAAAATTTTCCTGTATTCAGAAATTAGATAACGGGAGAAATCTTGAAGATTATACTGAATCCTT
EContig12203_1	GGGGATAGAAAGGGGGAGAGTGATAGAGAAACTCAACGGTACGATGGTGGAGAACGGTGTTATCACGGAAGAGCAGCAGAGGCAGATCGC[C/G]GATAACAGAAACGACGTCGTTACCAACCATTCGAAGTCCGGGGAATCTACTYCCCCTACCGATCGTCCCGTCCGCGTATACGCTGACGGAATCTACGATCTCTTC
EContig12584	CAAGAAAGCGAACTCGTGCACAAAGTTCTAAAATGACAGAGAGTGAACTGGAGGCTGATCATAGTGAAGTACGATCTGAAAGTGTCATAG[C/G]AGGTGGCATCAAGAAGAGGCGCCAAACAGGTGCTCCAGCTGTGCAAAATGTTGGCGAGAAGCGTTACAATCTACGGCAACGGAACCGCAAGAATGTTGGCACGGA
EContig1263	CCATAACGGTTCCCCTGTTCGAGGCAAGAATTACACGCATCCAATAGAATGGATCCGCACCTGTTGTGGAGTGTATGCCATACAATGGCA[A/G]CTGACTGCAAACCAGGAAAATGAAAAGAGATATCACAGTGTAGATGACCTTCTCTCTAAATGGAATTTTCCTATCTGCGCTCTGAACCTCGGGCAGGAAAGAAAG

Contig	Sequence
EContig1372_4	GCACGAAAACGCGCCCTTCAAATTYCCCGGCACAAAAGCGGCCTGGAACTGTTATTCAGTGGCATCAGATGCAGACTTAATAGCCGAGCA[T/C]CAAATTTGGAGCGCGGTCGACCCCAATGGGAAGAACGAAGGTTTCAACTGCAGCAATGGGGACCTTTTCAAGTGGAAGCATATGTGGAAGATATTGGCTGAACAA
EContig1634	TAGGATTCCCAAGGTCCAGCAATTGTTGCAGGACTTCTTCAATGGGAAGGAACTTTGCAAGAGCATCAATCCTGATGAAGCTGTTGCTTA[T/C]GGTGCTGCTGTCCAGGCTGCCATTTTGAGTGGTGAGGGTAATGAGAAGGTGCAGGATCTTTTGCTTTTGGATGTCACTCCCCTCTCTCTTGGTCTGGAAACTGCT
EContig1775_5	GAGGGTATTGCTGTTATTGATCTCAAGGATTCATATCCCAATGTCATGGAAGATTTACAGGCTTTGAAAGCTTCGGGCCAAATTTGGCTG[T/C]TATCGAACTTCGACTCACAGGAGGACATAGCATATCCAAACGATCCCCGAGCAGTTWTTAAAGTTGATGATGATCTGAAACAATTATTCAGGGGGATGGAACTCC
EContig181_2	TGCAGCCAAATTTGATGAGTATGCAAGGGGTATGATAAGCTCTTCCGGTTCTACCTTCTTTGAGGAGCTCGGATTGTACTACATTGGTCC[A/G]GTGGATGGGCATAATATCGATGATCTTGTTACTATATTTCAAAAAGTAAAGACAATGCCAGCGCCAGGGCCGGTTCTAATTCATATTATAACAGAGAAGGGAAAA
EContig1813_3	CAAGATTCAAAGACCGATCCCCTCATTTTTGGATCATATKTTCTTCATAGAATAAGTGACTCATCTAGCAAATTAGGTGCTTATTTTAAC[T/C]GAAAATGAGCTAGTATATTGGTGGGAGTCCCATAACTTCTTCAACTTCATGATTTGCAGATTCATCCAGAAGCCAATTCTCAAGAAATGACAGAGGAGGATTATT
EContig1816_1	AACTGATTCTGCAGTAGCAGTAGTACCCTCACAACATTCAGCAGTGGGATTGTTTTGAGAACCAATCTTGGGAGATGACATCCATCCTTT[T/G]AGTAATCGGGATATATTCTCTGCGCTTGAGGCATATATTGAATTAGAATTTTCGGTTAACTTGACGTCRCTTGATGAATCATTCTGGCTGCTACTGTTGACACCC
EContig1839	AATAAAGGAACGAGAAATATACCACTGGAACAATTTTATGTTTATTTTTAACACATTTCTTGATACAGACCAGATGGCGACAGACCGACC[T/C]TATATATTTGCTTATCAAAGGCTGCTAGAAGAGTCCCTACGTTCAGTGCAGCGTTCATAGATTGATTTCTCGACCGAAAATCCAGCTGAAGGGGGTGGCCGATGC
EContig188	CGAGGATCCTGCAGAAGGAGAGGAAGCCCTGGTTGCGAAATTCAAGAAACTTCACGAGGATTTGACATCTGGTTTCCGCAACCTCGATGA[T/C]GAAACTCGGTAATCGATGCATAGGTACTAAACAAATTCATGCCAAATCAGCTTTGTCGTCTGCTTTCGTGCTGGTGGGAAAACTAACTTATTGTGAAATTCTTAA
EContig1947_3	CTAAACTATATGAAAAATAAACAAGGGTCTCATAATTCATAACCATCCACCTCTCTCATTACACACACAATCCAACAAATAACATCAAAC[A/T]GAAAATAGGACAGATGAATTCAATTTCCATACTCGTTTAGTTGGTGGGGTTGCTCCTGAAGGATCCCAAAGCCTTAAGGGCTGCAGCTCTAAGGATATACTGGTG

Contig	Sequence
EContig1962_1	AACATATTGATATTGGATATGCTCCACAGATCTTCAGTGATGAAAACCCTAATGCATGTTCAGTTATGTGAAATTTGGGATGAACAAGGG[A/G]TGGAGTTAGGGGGCACACCGCACATGGATARCTGTTCCATGACATGTTGTTGTGCTCTACATGTGGTCATGTCTGGGTTTCGTAGTATAAATCTACAGGAAGCTG
EContig1987_2	GAATTAGAGCCTTCATCCTGAGACTGAGATGATCTCCTTTATCGTATTAACTAAACAGCCTCTCGAATCAAAGCAGATCTGGGGCGAATT[A/G]AGAAGCAAGTCAACTCCGAGTTCAAGTCAGAAATTATTTTCTTTTCTTTCCCGCACTTATTGACAATCCCCAACGATTGGTTGTATCGTCAGGGTGTCTCTATTC
EContig2068_2	TGATTTGCTAGCAATTTTAGAAGAATATCAGGTTAATRAATGCATATTTGTTGGCCATTCTTTGTCCTCCATGACTGGTGCTATAGCTTC[T/C]ATCTTTCGGCCTGATCTCTTTCAAAAAATCATCACGATTTCTGCTTCTCCAAGGTTTATAAATACTGAGGATTACTATGGAGGATTTGAACAAGAAGACATTGAG
EContig2172_2	ATTGCTAAATGAAGTAGTTTCACAGAGCAATTAAGGGAGTAAATTCGTGCTTTTGTGTGGCTCCTTGCCTGAATAGTCATTTAAATGCGT[T/C]ATGTGGCCTTTGCAAGGGAAGTGGTCGAGGTTTAAACTGGTTCTTGAAGTAGTTAATTCGTTAAATGAGAGTCTCTATTGATGGACCTTGAGTCTAGAGGTTCTT
EContig2305_1	TCTATCTGGATAAACACTTGAACAAAACTTCAGCAGATTATTGTAGGTATCTTCGGAATTCCCTAGAAATAGAGAGACAATAAAATAAGC[A/T]AAACAATTATAAGCTACATGTGTCGCGTCCAACCTTTGATCTTTGTTCGCTGTTGCTTTGTCACCATTGAACTTCAAATTCAAGGAGGCCCATCAATCGAGCTTC
EContig2458_2	AATAGTTGCAAGCTGGCTCTTATCACCCCATTCCCCAAAGAAAGTAATTGAAAATGCCTTCAGCAAGATGGGCGAGAAGAATTGAGCAAG[A/G]AAAGGACGGTTTTGCTTCTTCAAATTGTCATCAWCCTTATTGTTCTCTTTGGATGATCCACCATTACCTTTTATATCACCACCCAATTTTGCTTCAACTTCAGCC
EContig2514	GGATGTAGCATAAGCATGAGGAATATCCCAATAAATCACAGAGGCGACACTAGAAGTAAAGATAATGCTCCCTTTCTTGGCCGGAATCAT[T/G]ACCCTTGCAGCGTGTTTGGCGCATAGAAACATGCCAAAAACATTAACGTCGAAAACCCTCTTGTAATCTTCATATTCGGTAGCCAAAATTGATGTGTCGCCCTTG
EContig2705_2	AAAGTATCAAGTTACACATCTCAAGACCCACTGCGAGAAGTTCCTGATGTCCAGACTGAACTGGGAAAACTCACTACCCTATTATGCTTT[T/C]GCTCACCAGCACTCTGCAAAATGCTTGCTTGATGCATCATTGTCGTTGATCATGGACAACATGGACAAGCTTAGTAAGCGAGAAGAGTACATGGAACTTGTGGAG
EContig2785_2	AATGAATGAGTGATAATCCCAGAATCCAACGGCATGAGCCACTGGCGTATTCCTCTTAGTGAAAAGATTCTCAAACTGATAGGTCTGAAA[A/G]TAATCTGTAATCGTCAAATTACAGCAGTAAATCGACAACCCTTTACACTCCCATCCTTTGTCGCACATTTTCTTCCCCTTCACCTTCTTCACTCTCAACGTCGGA

Contig	Sequence
EContig2906_2	ATATGTGAATCAGCTTTGTGAGAGCCTCCCWAGTCCAGCGGGCGAGTCTGCAATTGACTGCGATTCCATGTCAGACATGCCAACTGTCAC[T/G]TTCACCATTGGAGGCAAAGCTTACCCTCTCACACCAGAACAGTATATTCTCAAAACTGGTGAAGGCATCGCCWCCGTTTGCATCAGTGGATTTATGGCTCTGGAT
EContig2937_4	GTATGTGGACACTTTTGTGCAGAAGGACGCTGCTGGACATGCAGTTGGGGCTGCAGCTTAAAACCGCCTTTTACAAAATCTTGGAAACTA[T/G]TGTCTTTAAGCATAAACTTAGCCGTATGGAGTTGCCGAATTTGTGTACCTTTGGTTGGACGTATGGCTTGTGTGTTATTAAATAAAATAAATGTGTTTTAAATTG
EContig2950_2	GAGTCGAGTACTATATTAGTCGAGTGTACAAGTTTACARTGGAGTGGGAACAGCTCTGAGAGGATTTGCTTTCTGCAATGTTATTCCAAA[T/C]TTCTCTTCCATATCCAACTCCTTCGGTGCAATTCCCCCTTCAAGCTTCCAATCAAATGAATTCAAAAGTGAACCCAACATTACAGGAACCATTCTAATAGCCAAA
EContig298_1	ATGGATTAGTCCATCAGCTTGGAATTCCCAGTCGATAACATAATCATAGTTAGCAAGTGACGCTACCATCCTTGCAACTAATGACACCTT[A/T]GGTCTTGCCTCTCGGATCTCCTCGCCTACTATTGGGCTCTCGGTGTGACGCCAAGCAGCATCACCCGAGTASCGCTCGAACAGACAGATCATGTTGGACCTAACA
EContig2998	CCAGTACTTGAAAGAGAAGGGTAGCTTTGGAGATCTTACTCTTGGTCTCACTCAGAATTTTGAACCCAAAGGAGCTCCAACGGCAACTGA[A/G]ACCATGAACTTATTCCCAGTCATTGGGAACTCTGAAACTCCGACCCATGAAAAACTCAACATGTCCATTATCCCTCAGAAGAAAACTGATCTCAGTAGGGACAAG
EContig3075	GAATTTCCGAAGGAAAAGTGTGGTGGGTTTGAACTTTGATTTTCTGCTCCTGAATTTGACGAAGCACTCGTCGTATCTCATCTACAACGC[T/C]TCCCTCTACTTTAGCTCCACCGTGCAGAGGCAGTACCGCCAAAAATTTGGCCTCAATGAGATGATACCTGTGGCTGCCAATGATGTAGCTTTCTCCATTCACGCT
EContig3144_2	TCGAATCCTAATGTTAGATATCTTTCCATATGGTTCAAAATGCCTTTCAAGATCTCTGGTCCTGGTACTGATAGGATCAAAGTTGATGAT[A/G]AACAAAGTTTTTGTTGGTCTTGTGTTAGCTGCAGATCTTCTTGATCTATCAGGCTTCCTGCCACCACGTTCTTGCTGCTTTGTCAGTTCAACACGAAGTCGGCGA
EContig3225_1	ATTTAAAGCTGCGTTTGCGCGGAATGATTTTGACAAGTGTAATGAACTCCTATCCCAGCTCAAGGTTCTGCTGACAAAATTGAAGAGTCT[A/G]CCTCCATTATTCGAAGAAACGCCAAATGCAATCCATGAATTGACACTAGCAAGGGACATATATGAGCATGCRGTTGTTTTAAGTGTGAAGATTGAGGATCTGGAT
EContig3597_1	AATCCGGTATAGCAGAAACGGTAACAATCATTTATTTTATTCCAAGACATGATTGGCTAAACCTATTCCTTTTTCGAGTAGAGACGAAGA[A/G]CAAATGCTAATCCCAATATCAATAASGGCAGGATAAATAGCAGTATCTTGGATGAACTCCCCGAGTCCTGGTTTGCAGTTGAAGTTGCACTTGGAGGTGGGGTGT

Contig	Sequence
EContig3889	TGGTATATTTTGTCTTGGATCTGGGGCTACAATTGTTCATGGAGTTCAAAACTTGTGGACTTCTCAGCCTCCTGAGCATATTGAGTATGC[A/G]GCTCTGGTGATTGGTGGCTCTTTCATTATGGAAGGTGCTTCTCTTCTTGTTGCCATCCATGCTGTCAGAAAAGGTGCTGCTGCGGAAGGAATGAATGTGAGAGAC
EContig3935_2	ACTGACGATCTGTGCACCGATCCGGAGGCTTCTGGAGGYTTGTTGTGGCCTCCAGCCTCCAGGTAGTGCTCTGCTCCTCCTTAATTCTCC[T/C]ATTCGCGATCTTCGGTAGAACAATGACTATTCTCCTCGTGTTGATTTCTGATCCACTAATGTTTTCACTTCGCTGTTGGTCGGACTGTAGTGGTGTTTGCTGATT
EContig4112	TTGCAGCATACCCATCCTTCGCTGCCTCATTATGATTCCACCGAGTGGGATTGGTTACGCGGAGCTTTAGCAACTGCTGACAGAGATTAC[A/G]GGATTCTAAATAAGGTATTCCACAACATAACGGATACTCATGTCGCGCACCATCTTTTCTCAACAATGCCACACTATCATGCAATGGAGGCAACCAAGGCAATCA
EContig4297_2	TTGTAGCATCTGCGGTGCTTCCAGAAATTTCGGACTCTGAAAGTTTACTAAGGTAAGTTGACAATCCCATCTTTGAAAAGCTATCGACCA[A/G]TTCAGCAATCCCTGGAGATTTAGATTCCAACTCAGCGAATAGCTTTGTGAGAGTTTCACAGAGAGGCTCCGGTACTGATTTCAAAATTTCAGAAATATTAGACTT
EContig435_10	GAGGCCTTATGGACTATGCATTCGATTTCATAATAAGAAATGGTGGCATTGACACTGAAGAAGATTATCCTTATAAAGGCTACGATGACA[A/G]ATGTGACCAATACAGGAAAAATGCCAGGGTTGTGTCCATTGATAGTTATGAAGACGTAGCTCCATATAACGAAAAGGCACTAAAGAAGGCGGTTGCAAATCAACC
EContig4380_1	GAAAAATGCCATGGCAGGTATGGACGSTGATATGATGGAAACGGAGGCAAACGCTCCGCCGCAACCGGTGGCTGTAAATGCCGACCAGAC[T/C]GACGCTGTTCGGAATTTGCTGATAATGGCTAGGCAACTCATCGATCAAAACAAGCCTTCTCAGTCTCTTCAAGCGGTGGTGATGGCAATGWGATTACAAGGTGGG
EContig4424_1	AAAATTTGTGAAATACTCCTTCTAAGAAAGCTGCATGGGAAATTGCAGGTCATGTGAGCCTAGGTCCACGGTCACAGCCAAGTTAATTCT[T/C]GTAGACGGAGAGCTAGAAGAATTTCCATGTCCAATAAAAGTGTCAAATTTGCTTGAACAAAATCCAGATTGTTTCATATGCAATTCAGATGAAATGGGATTTGGC
EContig4490	CCATTACCTCGAACAACGGCTGTGTATAGTCGGAAAATGTACGAGTCTCATCTATGCTTCTTGG[A/G]TTGATGCCCGAGGCTGCGAGATCCATCGCCGTGACGCGGTGGCCCTCCGCCTCCAGCAGTGATTGTAACCTGTACCAGCACCAAGCTCCATGGCACATACCATGCACTAGAACAAAATTGTTTTTTGTTGA
EContig460_6	ATAAAGAGCCTCAACTTAGGTAATGACGAGAAGGTGCRCTATTAGCTACTGTTTTCTCACTCATTCCCTTCTACTGTACATATTCGAGCT[A/G]TAGACTTGGGAGAATCTGTACTTCTGTCTACTAAGGAAAGCTGGCTGAGGATATATCTAAGAATTGTGCAAAAAACACACAATAACGAAGAGGTACCGAAGATCT

Contig	Sequence
EContig465	ATGATCTTCACATTTTACAAGGCACAAGGTCTTTCAGTTGGGTCGTCCCTAGTGGAAGAAGACAAGCTGGATCTTGCAACAACCCTTATT[A/G]AGAAGGCTAAGGCCAAAGGAGTCAGTCTCTTGTTACCTTCAGACGTGGTTGTGGCAGACAAGTTTGCTCCTGATGCAGATAGCAAGATTGTGCCGGCATCTGGTA
EContig4794	TGTTACGTCAGAGAGATCCTTCAGAGTTGTCCCAACATATACCAGGGCAAAAGTAATTGGCATCATTCCCAACCACGATGCTAGCATGTA[A/C]TGTCCAATGGAGACTGGAGTCACGGATAAGAGGTAGTTCAACATGTTAAATGGAAGTAAAGGAACAAGCCGAAGAAGCAAAACAATCTTGAATCCAGATCTCCGA
EContig504_6	TCGATATGAACCCTATCGATTTCTTCTGGATTTTCTTGGCTGGTGTCAAGGAGGACATCTAACACATCACTGTTTTTGCTATTATCGGAC[A/T]TCCTTCTCTTCAACCTTTCATTAATCAAGCCACCGAAAAGCTCTATGATCTTGCCAAAATGAATAGTCATGCGGCGCCGTATACCTAGAGGGTCTAACTTTTCAA
EContig5140	GAATGGACTTTCCCCAGTGGAGACTCAGTCTGATGGCTGCCTGCCCCTTTCCAACTCAATGGAAAGTCAATGTACTTCAGAAGAACCAAG[A/G]TCTTCACATAGTGGCCATGCAACTTTGTTTACTGATACGAGATGTGCAGATATTTCACTGTTTCCTGAAAGTCACGTAGAGTTAAGTGAGTACACAGAAGAGAAG
EContig515_2	TTCAGCTGACTCGGCACCAACTTCCTCGTAGTCCTTCTCCAAAGCAGCAAGATCCTCTCTCGCCTCAGAAAATTCACCTTCCTCCATACC[T/C]TCTCCAACGTACCAGTGCACAAAAGCACGCTTAGCATACATCAGATCAAACTTGTGGTCAATTCTTGAGAAAACTTCTGCCACACTAGTCGAGTTGGAAATCATA
EContig515_3	GATAACTGGCGCATATGATGAAAGCATGAAGTGGATCCTAGGATAGGGAACCAAGTTTGTCTGGAACTCATTCACATCAACATTCAAAGC[T/C]CCATCAAATCGCAGAGATGCTGTCAATGAAGAAATCACCTGAGAAATGAGCCTGTTGAGGTTAGTATAAGTTGGCCTTTCAATGTCAAGTGATTTGCGGCAGATA
EContig5168_5	CGATGACTCCATTTGCATTAATAAGAGCCTCACGAGCTAAAGCATTGATCTCCATAGTGTATCGCAAATGAGGATGTAATAGTTTGTAAA[T/C]CGGGTGCATCGCACTTAGTTGTCGATTTGTTGCAATTACGTAAGGCTCCGTACAACAATGAGTTCTTAGCCAGTGACTGACTAACTGATGAAAACCTGAGTCAAT
EContig5183	TGTGAAGCTAAAGAGTGCACTTGTCGGGAATGCTTCAAAGGTAAAGCAGATAACGCGGGTTTTCAAAGTGGGTAATGAAGAATTATCTTA[T/C]GTGGTCGAAATGGCTACCAATCTGAATGATCTTCAACCACATTTGGAAGCTTCTCTTAAGAAGATTTGATGCTCGGATGTCTAGCTTATAATAAATAAGTTTGCA
EContig5193	TGCTGGAGCTGCACCTGAAGAGAAATCCAGCGAGGAGGAGCTGACTGAAGATGAGTATGCGTCCAGTGATGAAGAAGTTGCCGGGAAAGT[T/C]GACCCCTAGGGTCTGAATTTGCAGTTCGATTGGCTCTTATCTGTACATGATATGTTACCTATGTTAGTAAACCTTAACTAC

Contig	Sequence
EContig5258	GCTCAATACTCGGGACAATATCTTTCAATGTGGAAGACAGGGTTTTGAAATGGGCAATTAGTTTCTGGCATTCAGTTTCATACTCTATAG[A/G]CGAAATAATATCACGAACATAAGCCTTCTCGAGTTTCTCTGTGGCCTTTATAATCGCAAAAAGGTCAGCAAAACTATCATACATCTCTCTTTCGCGCTTGTCATT
EContig526_2	TGTCAATGATTTCTTGGGTCCAAAGCTCACTGGTGTTGATGTCAGGAATCAGGCTGATGTTGATGCGATTATGCTGGAGATTGATGGAAC[T/C]CCAAACAAATCAAAATTGGGGGCTAATGCAATTCTRGGAGTTTCTCTTAGTGTATGTCGAGCTGGAGCTGGAGCAAAAGGGATACCCCTCTACAAACACATTCAG
EContig5399_1	CCCCGTCTCTTCTTTCACCTCAGCAAGATACTCAGCAAGCCTTCTAGCACATTTGGGTCCACGAACCTGGCTGAGAGCACAATGGAAAAC[A/G]AGGGTATCTTTGTCCTTGGCAGCTTCAATAAGACTGGGTATTTTGTCAAGAAACGTATCGCTAGCATAATGAAGTGACCCCGCTATGTGCCCATCGTAGTTCCTC
EContig5478_1	CTCAACCTCGACACTACTRATGTTTTCGCCTCCTGATATAATTATATCCTTTGATCGATCTTTAATTTCTATATAACCATCTGGATGCTT[A/C]ACACCAAGATCTCCAGAATGAAACCAGCCACCAGCAAAAGATTCTTCATTTGCCTTAGGGTTCTTCAAGTAGCCCTTCATTAACAAGAACAATTGCCCCGGAAAA
EContig5674_1	GCCATCAATAAGGATCTGAAAGCTGATACGAACGACGAATACCTCACCTTGCTGCGAGCAACTATTAAGTGCTTAACTTGTCCCGATAAG[T/C]ATTTTGAGAAAACTCTTCGTYTGGCTATCAACAAGATGGGCACAGACGAATGGGCTCTCACTCGTGTTGTTGTTACACGAGCTGAGGTTGACATGCAGCGTATCA
EContig6361	ATCACCGCAACAAAGAAAGAAACTCACGAGCAAAAACATGGCAACTTCAGCAATCCAACAATCTGCATTTGCAGGCCAGACAGCCTTGAA[A/G]TCCCAGAATCAGCTCATCAGAAAGGTTGGCAACCTCGGAGGTGGCCGCTTCACCATGAGACGTACTGTTAAAAGTGCTCCCCAGAGCATTTGGTACGGACCTGAC
EContig6479	TTGCCTTCTGTATTGGTGTATCTACCGTATTTGCAAGTATCACAGGGATATTCAGAGCATGGAGGAAGTCGAGAAGAGGTACAAGGAGCA[T/C]TAGAAGATTCAGTGGCTTAGCCATTCAGTTGCATCGATAGTGAAGCATATTATGCATCTACCACTGCCATTATTTTTCGTTTTACAGTTTATGTTTACTGTTCT
EContig6629	AGTGGTTCAAACTTGCTAAAAAGATAAATAACTGAGAGCTCCGTTGTTATTCAATCCAACTGGTGTATGCATCAATCTTAATAAAATCGA[T/G]CTGGCATTCGATGCTCACCAGAGCTCACCCTTGATTTTTGTCCTTTTCTTATTTCTTTGGGGTCTCATTTATGCTAGCAATGGTTACCCCGCTGCAGTTTCCTTG
EContig6851	TATTTTCTTGAAGGTGGATGTGGATGAACTAAAGGATGTTGCTAAAGAGTACAATGTTGAGGCCATGCCCACGTTCGTGTTTCTCAAGGA[A/T]GGGAAAGAAGTGGATAGGCTCGTGGGTGCGAGGAAGGAAGATTTGCAGGCTACAATCACCAAGCATGCTACTGCTACTGCTACTGCTACTGCTAGTGCTTGAGGT

Contig	Sequence
EContig695_2	AAGCTATCGTTGTTGCTCATATTGAAAACCTAGAGAGAAAGTGAAAAACCCTCAGAAATGAAAAGAGATAGAAGAGAGAAAAGTAGTATC[T/C]AAGCCGTAAGCGCCTTCGAAGCTTTTGCTTCTCCTCTTCCTTCGCTCTCTTCTTCTCCCCCCGT
EContig698_3	GCTAAGGGCCCAAGGTTGTGGTATGGGAGTCGCAATGAATAGATTCATCAGTGGGGTGATCTTAATGTCATTTATATCCCTGTACCAGAG[T/C]ATTACAATTGGAGGGGCTTTCTTTTTATTTGCAGGGGTTGGAATTATAGCTTGGATTTTCTTCTACACATTGCTACCTGAGACGCGGGGAAGAAACCTAGAGGAT
EContig6995	TATTACGATGCTTGTATATTTCAATATATTTAGTTAAAAATAATAGATGGATAACCGTTATCCTCGAATCAAGATTACTGTTTGTAAATC[A/G]TGCTTAATTCTTAATGCAATTCAGCAAGATATTTAATATTTTTTTCTCTAGTCTATCTTACTGATCCAGCCAACAGTGAATAAATGCACAGTGATCTTGCTCTCA
EContig7442_2	CGTCRATTGTCCACCGAAAGTGCTTCTAAGATGATAATCTATTTCGTTGATGGTGAGTTCTTCTTTTCCTCATTTATTCAGTTTTCGTGT[T/C]ATGATTCAAGTGAACTCAGTTTGTTGATGTGGACAAGTTTTGAAGCGTGGTTATGAAAGGAAGGGATGATGTAATTCTAAATTCCGTGTCTCAGCCCGTCCAATT
EContig749_1	TATCTGTGATAGTTGTGCAGAGTCATCTGATTCTGGGTGGAGAAGTAGCTGTTTTCTTATGAAAGGTGGCAAGAATTCAAACAATGCAGA[T/C]GCCCAAGGTGACAATTGAGAAGAAATGTTATCCGCAGAGACACCTTGCCTGAGCAAACCATGGATTTCCTGCAATAAAGCATACACTTCAGGAATGCTTTCAATA
EContig8421_1	CATAAACAAGCTAAGGCCTAAGGGTGAGTAGAGTTAAACGACACACAAATTACATCCAGCGCSAAGGGATCACCAGCAGCGTGTGTGAGA[A/G]AGAAGGGGGAGAAAAATGACGCTAAAAGTGTATGCAGATCGGATGTCTCAACCTTCGCGTGCAGTAATTATCTTCTGCAAAATAAATGGGATTGAATTTGAAGAG
EContig845_1	TAATCCACCGTCCCGACCCAAATCCTACGACCCAGAAATCATAGCCATCAAGATGCCATGACTGCATGGTGTTCTCATTATTCTGCAAAA[T/C]GACTTCGATAAATTCGTGGAGAGAAGCTGGCATTACAGATGTAGCAAAGTATGGAGGACCATCAGAGGGGAAGCTCTGGATGGAACTCAGGCTGAAAACACCGGG
EContig86	TCTTAACAGGAAGAGACCGTCAGTCCTCGCCCTGTCTCGTCAAAAGCTGCCCCAGCTTGCTGGAACATCCATCGAAGGAGTAGAAAAGGG[A/C]GGCTACATCATATCAGATAATTCATCCAGTAACAAGCCCGATGTCATTTTGATTGGAACAGGTTCAGAGTTGGAAATTGCAGTTAAGGCTGCTGACGAACTCAGA
EContig8662	CTGGACGACATCGTCCAGCCCTTGAGCCTCTTTCAGAAGTCGCTTATTCTTGGTCTCAAGAACACTAGCAGCAAGGAAAACAGCAAGAGC[A/G]CCTCTTTGGTCTTTCCATCCAGTTTGCACATTTTTCCTCTCAAATTTTCCATACTGCTTGAGCATCTTTCTGTTTACTTTAGCTTCGTCATTTTTGTTGGTTCGA

Contig	Sequence
EContig894_1	GCTGCAAAAGAATCTCAAGGGAACAAATTACAACCTCTATGTTCACAGTTACTTGAAATACGGTAGCAAGGCAGCTCGAGCTGAGATTTT[T/G]AAGGCTTCTAGAAACTCCACGAATCCTTGCATTTTGGAGGGATATGAAGGTACCTACACATATGGAGGAGTTGTTTATARAGTATCAGCTCCTCGAAAAGGTRCA
EContig8993_1	CGTCGATTATGCCTTCATTGTTGCACTCGTGGTCATTCTCGAGGAAATTAACGAGGACAGATCGGGTGAAGATTGAAATCACCATCAGAC[A/G]ATGATCGTGTTATTCCGTAAATGTAACCTCATCCATGTATTTGGATATTTCCCTTCAACCAATCATGTCAAATAAATGTAGAGTTGAAGGAGAGATCTGAGCCCG
EContig9136_3	CATACAGCAGGGCATATCTAATGGTTTACCAGATGTWGATTCGGTTTTCTATTCAACTAGGAAGACCGGYTCGGTGCCATTTGATATAAG[A/G]TTTGATGAACAAGCACCAAAAGTTGCCTTGCCCCGAGATACTATGGTGCCTATAAATTCATTCAATACATTGTTTCATTATAATGCATTTTGGGCTTTGATGCTT
EContig9157_1	GTGGCGGAGCCTGTGCCTGTACCTGTACTGACAGTTGATCAATAGAATGCTGAAAAAATCTACCGTGAACTCTAATGTGTATCCCATCAG[T/C]GCCTTTCCTGTTTCTTTCAATTGTTACTCTCTGCCCTGCTTACCTCACCTTMTGCTGGTGAAATGGGTTTGGTGTGGATATTTCATGTTTTACATAAAGCCTGTT
EContig9302_3	ATGAACTGAGGTGGCTYAAAGCCAAATATMAAATGCAGATGAGAGAGCTTAAATTTGGAGATCAACATAAGGGGATCGTGTCAAAATCTT[T/C]GACTTTGAAGTTCAATAACGAGAAGGATAAATTGTCACCGAGCTCCTCAAGTATGTCGCCAGAAGAGAAGCGTTCKACCTCATTTTTCCCGGCTGATGCAGAAAA
EContig9302_4	CGTGTCAAAATCTTYGACTTTGAAGTTCAATAACGAGAAGGATAAATTGTCACCGAGCTCCTCAAGTATGTCGCCAGAAGAGAAGCGTTC[T/G]ACCTCATTTTTCCCGGCTGATGCAGAAAACAACTGTGCTAACCAGAAAGTTCACAGTTATGAATCTGTTTATAATTCTTTTAGTCCAGTACATATGATCACTGCA
EContig9677	TCGACACGAGGTTGAATCTGATTCTATGACATCCATTCAGGATAGAGGGAAACAAATCCGGAGTCCCAGGGAAGTAATGGATAACTGTCC[C/G]GAATTGAAGGAAGCATCAAAGGAGAACATCAAAGACAGTGTTGATACCGATTCAGAATTAAGGAATTTCGATCTGAATGCTGGAGTGGATGAGGTTGCAGATAAG
EContig9922_3	CTGAGTGGTCAAAACTTGCCCRGAAGGCCAGAGTTTAAYGCATGTATTGACAATTGAGGTTTGATTACTGCAATTGTTCTATATTGGGTG[T/C]TACTGAGGCATTTGTTTGAATTTGAGTTGTATCTTTTGACTAGGATTTCCTGCTAAGGCATGTT
IContig1226_3	AGGCCTGGTTGGTGAAATCATTGGCAGGTTTGAGAAGAAAGGCTTCACTTTGAAAGGTTTAAAGYTCATCACTGTGGATCGCCATTTTGC[T/C]GAGCAGCATTATGCTGACTTGTCTGCAAAGCCTTTCTTTAATGGGCTTGTTGAGTATATCATCTCTGGCCCAGTTACGGCTATGGTTTGGGAGGGTAAGAATGTG

Contig	Sequence
IContig125_1	TTCCTTAGTTTTGCTCTTCAGTTTAAGCTTTGCTCAACAGCAGCTAAAGGGCCAGTGCCAAATCCAAAAAATCAATGCCCTTCAGCCCCA[A/G]TACAAAATTCAATCCGAAGGTGGAGTCACTGAGTCCTATGATTACAATGATGATCAGTTCCGGTGCGCCGGTGCCGCCTTTTTACGCCATACAATTTACCGCAGA
IContig1260_3	AGTCAAGGACGACATGTTCTGCTTGTTTGAAGGAGCGCTCCACAACTTGGGGAGTCTGAAACAACAATACGGCCTTGCAAAGTCTGCCAA[T/C]GAGGTACTTTTGGTGATTGAAGCCTACAAGGCACTTCGTGACAGGGCACCTTACCCTGCAAACCGAGTGGTTGGCCACCTCGAGGGGARTTTCGCGTTCATGGTA
IContig19_3	TATGCTGGACTTTGTCGACGATGCTGAGAAGCYGCACCACCCGTCGCGGGCGTATATTCGGGACACAAAGGCCATGAAAGCTACACCAGC[A/G]GACATCATAGAATACCCAAATGCTTACCATTTTGTGGTGGATATGCCAGGGCTGAAAGTTGATCAAATCAAGGTACACATAGAGGAMGGCAATGTCCTGGTGGTG
IContig2083_2	TGATGGGAGCTGCTTGTACACATTTTAATTTTTATCTGTGTTTTGGGTGCTGGAAATTATTAGACATTTATGAGCCAACACTATGGGAAC[C/G]TAGACTATTTGGTTCCATTGGTTTAATGATTGTAGTCCTATGATTTTGTGTTGTGTAATACAGTATATTTCTTTTAATCTTATAGTAACTTTTGATGTG
IContig2350_3	TACCATTTCTGGTACCGGAATCCAGTTCAAGAGATCAAAAACGTCAAAATAGGAGGAATTCTAGGTGGGCTACCCGAAATCGGGTTTCCC[A/C]ATTGGGAGAAGCTCAGGTTATGAGGAAAGCTGAGTTGGGTTTTGCGAAAGAAGAGGACTCTGCGGGTAGTTTTGACCAAGCATTGAACCCTGATCTGAAAAATGA
IContig257_8	CCTGCAAACTGGAAACCTGRAGAGCCCGTGGTRATTGCTCCGAGTGTGTCGAACGAAGAGGCTAAAAAGATGTTYCCACAGGGTTTCGAC[A/G]CTGCTGATCTTCCTTCGGGGAAGGATTACCTGCGATTCACAAATGTCTGAGCATATACATATACATATATCTGCAATTTCGGAATTGTCTGAATATTACTAGTAA
IContig2640_3	CTTACAGCAGGACTGATTAGCTTCAGGCRGGGAAATTCACATTTAGGTCAAAAGCTAATGAGAGCTCGTGTGGTCGTCCAAGGCGCGACT[A/G]TTGCCCTTATGGTTGGGACTGCTTATTACTATGGGGAGAAATTTTAACTTGTACTAGAGTATGAACTGCCAATGATTACCATGTTCATTCATATAGTGTACTAGT
IContig2753_2	GCTCAGGCTGCTGGTCGTGGTGCTGCGCCTGGCAGGGGTGCTGTTCCACCAGTGAGGAGGTGATCACTTCCCTGATTTGGTGTCTCACTT[T/C]GAAGTGTCGTCGTGCACAAGTGATAATCCTTGTTTCCAATGATAAAATTGTCAGTGGTGTACAATGAATTAACCTTCTTCCAACGATTACTTAAGATGGTTTTCA
IContig2783_3	TTTCTCGACATTGAGCGCGTGTGATCCTGCCTTTGCTGCACAACAGATTGCTGAGATAGCCGAAGGTGATAGTCGGGGACTAGCACTTCT[A/G]ATTCCGATCATTCCGGCCATAGCTTGGGTACTCTACAACATCCTGCAACCAGCACTTAACCAGATCAACAAAATGAGAAGCGCCAAAGGAGTTATTGTSGGACTC

Contig	Sequence
IContig2981_4	TACTTGAAAAGTACTATGGGGAAGCCRCAGCGTATTTTTTAAGAAGATATACATCTCAGCCACAACTGTTTTGGGAGAATTGTACGAGTT[T/C]AAATAATTAAATGTTTCCAAAGACGAGTTTTGAATGTCTATCATCTTGTTTCGTCATGGACGTTGTTTCCCTCAATGTTTCTGGGTTGAAGCTTACTATTATTAA
IContig3160_2	AAAACATACGAGATATTCGTCGGAGTATGATGAGCGGTGGAGATCAATCGAACAAGCAAGACAACGTAAAACCAATTGAAGATAAGCCGC[C/G]GGCGCCCGTGGTGGCTTGGAATCCGTTGTTTCAACCAGAAGATTTCATGGATGATCCGCCGCAAACGCCTCTTAATTTTACAGCTCAATTAGATTCCGGCGGCCA
IContig353	ACCGGTCGCCGGCGGGCGAAAAGAGTGAACCCAAGGATGAGAAGGTGGAGATAACTCATGTATTTCCGTCAGATGACAAGCCCGGAACTC[A/G]TAAAGTTGTCGGTGAAGCTGTTGGCAAGGTGGTCGAAAATGTCCAGTCGGCAAAGGAAGCAATGTCTAAGACCCAAAAAACTGAGTAATATACGAGAAAAATTTC
IContig3651	GCCTCATCTAGATCCATCATCTGGCAGAATGAAAGAAGTCGTTGATTACAGAGTCCTATCTCCAGGAGACCTAGCCAACTCTGTGATGAC[T/C]GAAGGGGGATGGTCGGACTTCCTGTGCATGAGAAAGGAAGCTCAGCCAGCTCTGGATCTTGCACTTATTTTTGTAGGAGAACAGTTGCAGTCTCTGGATATATCT
IContig52_2	GCAGCAGCTCTACCACTGCAAGATGTATTTAACTCAACGGAGTCCATCTGAAAAAATTTCACTCAGAAGTTYCAAGAACCTACAAACAGA[C/G]GAGCATCTCAACGATTGCTGCCAACAATTGGAAAAAATCAAGGAGGGTTGCAGGYGCAGAAACCTGAAGCGAGTTGTGCAGCAGCAACAGCAGGAAGAAGGAAGC
IContig667_1	ACGGCCGGGGGCATTCAATTCATATATAGCTTCTCTAATATTTTGAAAATTCAAG[A/G]GAGTTTGCAAATGGCTGAGCAGTATCAGCAACAACATTATGGGCAACAGCAGAAGGGTCAGCAGCAGCAACAGAAGCAGCCGGTGTCCCACCAGGTGGTGAAAGCGGCGACAGGCGCCACAGCCGGCGGTTCCCTCCTGG
