# Supplementary material for: Utility of EST-SNP Markers for Improving Management and Use of Olive Genetic Resources: A Case Study at the Worldwide Olive Germplasm Bank of Córdoba
Source: Plants (Basel). 2022 Mar 29;11(7):921. doi: 10.3390/plants11070921 (PMC9002360; doi:10.3390/plants11070921)
Supplement: Supplementary file 1 [file plants-11-00921-s001.zip › Table S3_revised.pdf]

Supplemental Table S3: Accessions included in each synonymy group detected by means of the 96 EST-SNP markers and previous bibliographic references.

Accesions/cultivars included in each synonymy group	Previous bibliographic reports of synonymy	Sinonymy Group
*Ileña_1** (PER), ***Passulunara***** (ITA)		1
Filare (ITA), **Moraiolo** (ITA), Moraiolo T. Corsini (ITA), Morcaio (ITA), Petit Ribier (FRA), Tondello (ITA)	Filare ([3]; [24]; [29]), Moraiolo T. Corsini ([3]; [6]; [16]; [24]; [30]), Morcaio ([3]), Petit Ribier ([79]), Tondello ([3]; [24])	2
Olivar de la Marina (ESP), Olivo Macho de Santisteban Pto (ESP), **Picual** (ESP), Picual de Hoja Clara (ESP), Picual de Hoja Oscura (ESP),	Olivo Macho de Santisteban Pto ([6]; [16]; [24]; [42]), Picual de Hoja Clara ([6]; [16]; [24]; [42]), Picual de Hoja Oscura ([6]; [16]; [24]; [42])	3
Alameño de Marchena (ESP), Cañivano Blanco (ESP), Hamrani (MAR), Haouzia (MAR), Menara (MAR), Misión de San Vicente (MEX), Mission Nieland (USA), **Picholine Marocaine** (MAR), *PM-M16* (MAR), *PM-S8* (MAR), Sigoise (DZA), *ZDH4* (MAR), *ZDH6* (MAR), Zeitun lkhl (MAR), Zitoune DK (MAR), ZZ 10 (MAR)	Alameño de Marchena ([3]; [6]; [16]; [24]), Cañivano Blanco ([6]; [16]; [17]; [80]; [81]), Hamrani ([3]), Haouzia ([3]; [6]), Menara ([3]; [6]), Misión de San Vicente ([3]; [6]), Mission Nieland ([3]; [6]), Sigoise ([3]; [5]; [6]; [17]; [82]), Zeitun lkhl ([3]), Zitoune DK ([3])	4
Dafnelia (GRC), **Lechín de Granada** (ESP),	([3])	5
Dužica (MNE), *Esek Zeytini (Odemis)* (TUR), **Gaydoyrelia** (GRC), Grossa di Spagna (ITA)	Grossa di Spagna ([24])	6
Augellina (ITA), Baladi (Ain) (LBN), Baladi Tawil (LBN), Cailleitier (FRA), Correggiolo di Pallesse (ITA), Corsicana da Olio (ITA), *Dan* (SYR), **Frantoio** (ITA), Frantoio A. Corsini (ITA), Jlot-1965 (LBN), Larcianese (ITA), Maelia (ISR), Oblonga (USA), Puntino (ITA), Razzo (ITA), Razzola (ITA), San Lazzero (ITA)	Augellina ([1]; [3]) Baladi (Ain) ([3]), Baladi Tawil ([3]), Cailleitier ([3]; [79]), Correggiolo di Pallesse ([3]), Corsicana da Olio ([3]; [24]; [83]), Frantoio A. Corsini ([3]; [6]; [16]), Jlot-1965 ([3]), Larcianese ([1]; [3]), Maelia ([3]; [6]; [24]), Oblonga ([3]; [6]; [81]), Puntino ([3]), Razzo ([3]; [30]), Razzola ([3]; [30]; [85]), San Lazzero ([3])	7

Accesions/cultivars included in each synonymy group	Previous bibliographic reports of synonymy	Sinonymy Group
Kelb et Ter-144 (SYR), **Lechín de Sevilla** (ESP), Mawi Stanboli (SYR), Nevadillo de San Vicente (MEX), Nevadillo Valle las Palmeras (MEX), S. George Greys (USA), *Souri-1589* (SYR), Zarza (ESP)	Nevadillo de San Vicente ([3]; [6]), Nevadillo Valle las Palmeras ([3]; [6]), S. George Greys ([3]; [6]; [86]), Zarza ([3]; [6]; [16]; [42])	8
Confetto (ITA), Majorca (ITA), Manna (ITA), Nera di Gonnos (ITA), **Nocellara Messinese** (ITA), Sivigliana da Mensa (ITA), Tonda di Cagliari (ITA)	Confetto ([1]; [3]; [83]; [87]; [88]; [89]), Majorca ([3]; [24]), Manna ([29]; [83]), Nera di Gonnos ([3]; [24]; [29]), Sivigliana da Mensa ([3]; [24]), Tonda di Cagliari ([3]; [24]; [29])	9
Corsicana da Mensa (ITA), **Itrana** (ITA), Nera di Oliena (ITA), Paschixedda (ITA), *Pulazeqin* (ALB), Terza Grande (ITA), Terza Piccola (ITA), Tonda di Villacidro (ITA)	Corsicana da Mensa ([83]), Nera di Oliena ([3]; [24]; [29]; [83]), Paschixedda ([83]), Terza Grande ([3]; [24]; [83]), Terza Piccola ([29]; [83]), Tonda di Villacidro ([83])	10
Cordobés de la Aliseda (ESP), Cordovil de Castelo Branco (PRT), Corneja (ESP), Enagua de Arenas (ESP), Llorón de Iznalloz (ESP), **Verdial de Badajoz** (ESP)	Cordobés de la Aliseda ([6]), Cordovil de Castelo Branco ([3]; [6]; [24]), Corneja ([3]; [6]), Enagua de Arenas ([3]; [6]), Llorón de Iznalloz ([3]; [6]), Verdial de Badajoz ([3]; [6]; [24])	11
**Alameño de Montilla** (ESP), *Castacabra* (ESP), Marteño de la Carlota (ESP), Nevadillo Blanco de Osuna (ESP), Zarzariega de Orcera (ESP)	Marteño de la Carlota ([6]), Nevadillo Blanco de Osuna ([16]; [24]), Zarzariega de Orcera ([6]; [24])	12
Bella di Spagna (ITA), Giarraffa (ITA), **Gordal Sevillana** (ESP), Gordal Valle las Palmeras (MEX), Pizzo di Corvo (ITA), Santa Caterina (ITA), *Ters Yaprak* (TUR)	Bella di Spagna ([24]), Giarraffa -Pizzo di Corvo ([29]; [91]), Gordal Valle las Palmeras ([6]), Santa Caterina ([6]; [16]; [17]; [24]; [90])	13
**Americano** (ITA), Firenzuolo (ITA),	Firenzuolo ([3])	14
Manzanilla de Ayora (ESP), **Pequeña de Casas Ibáñez** (ESP)	Manzanilla de Ayora ([6]; [16])	15
Arancino (ITA), Ciliegino (ITA), Pesciatino (ITA), Rosino (ITA), **Rossellino** (ITA)	Ciliegino ([3]), Pesciatino ([3]), Rosino ([3]), Rossellino ([3])	16

Accesions/cultivars included in each synonymy group	Previous bibliographic reports of synonymy	Sinonymy Group
Rosciola (ITA), *Rossello* (ITA)		17
Bjelica (HRV), *Maiatica di Ferrandina* (ITA), **Ulliri i Bardhe i Tiranes** (ALB), *Ulliri i Bardhe Kruje* (ALB), Žutica (MNE)	Bjelica-Zutica ([40])	18
Gremignolo (ITA), *Kallmet Kokerrvogel* (ALB), **Leccino** (ITA), *Manzanilla Chilena* (CHL), *Palmarola* (ITA)	Gremignolo ([3])	19
Ginestrino (ITA), **Maurino** (ITA),	Ginestrino ([3])	20
**Faresana** (ITA), *Olivo da Mensa* (ITA)		21
**Bosana** (ITA), Olieddu (ITA), Palma (ITA), Sassarese (ITA)	Bosana ([3]; [6]), Olieddu ([3]; [83]), Palma ([3]; [83]), Sassarese ([3])	22
Antawi (SYR), *Baladi Remadieh-1769 (* LBN), *Celebi (Silifke)* (TUR), Cirujal (ESP), *Dal* (LBN), Dan-136 (SYR), *Dilmit* (TUR), *Erkence* (TUR), Grossolana (ITA), *Hurma Kaba* (TUR), *Kanabisi* (JOR), *Marksi* (ALB), **Safrawi** (SYR), *Sari Habesi (Hatay)* (TUR), Shami-141 (SYR), Throubolia (GRC), *Yag Zeytini* (TUR)	Antawi described as molecular variant of Cirujal ([3]), Dan-136-Cirujal ([3]; [6]), Grossolana-Cirujal ([3]), Safrawi-Cirujal ([6]), Shami-141-Cirujal ([6]	23
**Bianchera** (ITA), Istarska Bjelica (HRV), Istrka Belica (SVN), Plementa Bjelica (HRV)	Bianchera ([3]; [41]), Istarska Bjelica ([3]; [24]; [41]), Plementa Bjelica ([3]; [6]; [41])	24
Barouni-1823 (TUN), Besbessi-1870 (TUN), Novo (ISR), **Uovo di Piccione** (ITA)	Barouni-Besbessi ([43]), Novo-Uovo Piccione (Olea DB ([1]; [3]; [6])	25
Kokerrmadhi i Beratit (ALB), **Konservolia** (GRC), *Sant' Agostino* (ITA), *Tekirdag Cizmelik* (TUR)	Sant' Agostino syn of Amphissis ([3]) which at the same time is syn of Konservolia ([1])	26
**Hojiblanca** (ESP), Verdial de Montefrío (ESP),	Verdial de Montefrío ([6]; [24])	27
Blanquette de Gastu (DZA), Blanquette de Guelma (DZA), Chaïbi Antha (TUN), **Chetoui** (TUN), *Chetoui Tunis* (TUN), *Chetous* (DZA), *Sayali T-1677* (TUN)	Blanquette de Gastu ([1]), Blanquette de Guelma ([3]; [92]; [93]), Chaïbi Antha ([3])	28
Jaén-1 (ESP), Negrillo de Arjona (ESP), Nevadillo Negro de Jaén (ESP)	Nevadillo Negro de Jaén ([6])	29
Carrasqueño de Alburquerque (ESP), **Carrasqueño de Elvas** (PRT)	([16])	30
**Callosina** (ESP), Cornicabra Murciana (ESP), Dehbia (MAR)	Cornicabra Murciana ([6]), Dehbia ([3])	31

**Accesions/cultivars included in each synonymy group**	**Previous bibliographic reports of synonymy**	**Sinonymy Group**
Nevadillo Blanco de Lucena (ESP), **Tempranillo de Lucena** (ESP)	([16])	32
Cakir (TUR), *Hurma Karaca* (TUR), **Valanolia** (GRC),	Valanolia-Cakir ([3]; [6]; [16])	33
Hojiblanca de Navarrés (ESP), *Ocal-25* (ESP), *Tomatillo* (ESP)		34
**Gerboui** (TUN), Marsaline (TUN),	([6])	35
**Drobnica** (HRV), Morchiaio (ITA), Razzaio (ITA), Sitnica (HRV)	Drobnica-Morchiaio-Sitnica ([3])	36
Beldi (TUN), Fakhari (TUN), Meski Zarzis (TUN), ***Nocellara del Belice*** (ITA), *Zarrazi* (TUN)	Beldi-Fakhari ([33]), Meski Zarzis -Fakhari (Tatouine) ([93]), Zarrazi syn of Giarfara ([3]) which at the same time is syn. of Nocellara del Bellice ([1])	37
Blancal (ESP), **Bolvino** (ESP), Negral de Sabiñán (ESP), Verdilla de Calatayud (ESP)	Negral de Sabiñán ([16]), Verdilla de Calatayud ([6])	38
Picudo de Montoro (ESP), **Varudo** (ESP)	Picudo de Montoro ([6]; [16]; [24])	39
Coratina (ITA), *San Pedro* (CHL)		40
**Abunara** (ITA), Biancolilla (ITA)	([3])	41
**Moresca** (ITA), Nerba Catanese (ITA), Olivo di Castiglione (ITA)	Nerba Catanese ([3]), Olivo di Castiglione ([3])	42
Abou Chawkeh (LBN), *Balah* (LBN), Jlot-976 (SYR), **Picholine** (FRA)	Abou Chawkeh ([3]), Jlot-976 ([3]; [6])	43
Cordobés de Arroyo de la Luz (ESP), Gordal de Granada (ESP), Gordalejo (ESP), Kalb el Tair (LBN), Manzanilla de Jaén (ESP), Manzanilla del Centro (ESP), Nabali (ISR), Nabali Lari (LBN), Nabali Muhasan (JOR), Rasei (JOR), Rosai-1809 (JOR), Soury Lari (LBN)	Cordobés de Arroyo de la Luz, ([3]; [6]; [16]), Gordalejo ([3]; [6]; [16]), Manzanilla de Jaén ([3]; [6]; [16]), Manzanilla del Centro ([3]; [6]; [16]), Gordal de Granada ([3]; [6]; [16]), Nabali ([3]; [6]; [16])	44
Adramitini (GRC), **Ayvalik** (TUR), Beyaz Yaglik (TUR), Edremit Yaglik (TUR)	Adramitini ([1]; [24]), Ayvalik ([42]), Beyaz Yaglik ([16]; [24]; [81]), Edremit Yaglik ([6])	45
**Kaissy** (SYR), Khalkhali-789 (SYR), Shami-953 (SYR)	Kaissy ([1]; [3]), Khalkhali-789 ([1]), Shami-953 ([6]; Baldoni et al., unpublished data)	46

**Accesions/cultivars included in each synonymy group**	**Previous bibliographic reports of synonymy**	**Sinonymy Group**
Bouchouk laghlid (MAR), Gatuno (ESP), Gordal de Archidona (ESP), Gordal de Écija (ESP), **Ocal** (ESP), Picual de Estepa (ESP), Verdal de Alhama (ESP), Zms_1 (MAR)	Bouchouk laghlid ([3]), Gatuno ([6]; [16]; [24]), Gordal de Archidona ([3]; [6]), Gordal de Ecija ([42]), Picual de Estepa ([16]; [24]), Verdal de Alhama ([24]; [16]), Zms_1 ([3])	47
Manzanilla Picúa (ESP), **Menya** (ESP),	([6])	48
**Gerboua** (TUN), Gerboui-1551 (TUN),		49
Chesna (ESP), Dulzal (ESP**), Manzanilla de Sevilla** (ESP), Manzanilla Valle las Palmeras (MEX), Redondil (ESP), Redondil (PRT), Stanboli-991 (SYR), *Stanbouli* (SYR)	Chesna ([6]; [16]; [17]; [24]), Dulzal ([6]; [16]; [24]), Manzanilla Valle las Palmeras ([6]), Redondil ([6]; [17]; [42]), Stanboli-991 ([6]; [16])	50
Diferente (ESP), Jaén_2 (ESP)		51
Carrasqueña de Cañada (ESP), *Fraga* (ESP), *Morruda de Salinas* (ESP),		52
Aimel (Setif) (DZA), ***Limli*** (DZA)		53
Chemlali Nord (TUN), Chemlali Ouled Youssef (TUN), **Chemlali Sfax** (TUN), Mellahi Kotti (TUN), Zalmati (TUN), Zalmati Zarzis (TUN)	Chemlali Nord ([41]), Chemlali Ouled Youssef ([93]), Mellahi Kotti ([93]), Zalmati ([1]; [33]; [98]), Zalmati Zarzis ([93])	54
Arauco (ARG), **Azapa** *(CHL), Sevillana (Azapa)* (CHL), *Sevillana (Tacna)* (CHL), *Suca* (CHL)	Arauco ([6]; [24]; [81])	55
Cerasuola (ITA), Zarrazi Douirat (TUN), Zarrazi Injassi (TUN), **Zarrazi Zarzis** (TUN), *Zarrazyet Sned* (TUN)	Zarrazi Douirat ([1]), Zarrazi Injassi ([1]); [94]), Zarrazi Zarzis ([94])	56
Abou Satl Mohazam (SYR), *Mohazam Abou Satl* (SYR)	([1])	57
Chemlali Mahares (TUN), **Injassi Hchichina** (TUN),	([93])	58
Chemchali (TUN), Chemchali Gafsa (TUN), Ferkani (DZA), **Jemri Bouchouka** (TUN), Mekki (DZA)	Chemchali Gafsa-Jemri Bouchouka ([93]), Ferkani-Jemri Bouchouka-Mekki ([3])	59
Boukaila (DZA), **Bouricha** (DZA), Hamra (DZA)	Boukaila-Bouricha-Hamra ([3])	60
Derras (TUN), **R'Khami** (TUN),	([3])	61
Chemlali Bent Louzir (TUN), *Khchinet Sig* (TUN), ***Zeitoun Boubezzoula*** (TUN)		62
Abani (DZA), **Aeleh** (DZA)	([3]; [67])	63

**Accesions/cultivars included in each synonymy group**	**Previous bibliographic reports of synonymy**	**Sinonymy Group**
Aggezi (EGY), **Aggizi Shame** (EGY)	([1])	64
**Koutsourelia** (GRC), Rachati I.Y.E.X (GRC)	([44])	65
**Aghchren de Titest** (DZA), Atounsi (Setif) (DZA)	([3])	66
Majhol-1063 (SYR), ***Tebabs*** (SYR)		67
Royal de Belchite (ESP), **Royal de Calatayud** (ESP), Verdeña (ESP)	Verdeña ([95])	68
Falsa Gordal Sevillana (URU), *General Hornos* (URU), ***Liguria*** (CHL)		69
Cordovil de Serpa (PRT), **Madural** (PRT)	([3]; [6]; ([16])	70
Manzanilla de Tortosa (ESP), **Morrut** (ESP)	([3]; [6])	71
Sevillana (ESP), **Sevillana de Abla** (ESP)	([6])	72
Royal (ESP), *Royuela* (ESP),		73
Girit Zeytini (TUR), **Koroneiki** (GRC)	([16]; [24]; [46])	74
**Blanqueta** (ESP), *Blanqueta Gorda* (ESP)		75
**Changlot Real** (ESP), Torcio de Cabra (ESP)	([6])	76
Ayrouni (LBN), **Verdial de Huevar** (ESP)	([6])	77
Ahia-Ousbaa (DZA), *Balbal* (DZA)		78
Oueslati T-1919 (TUN), ***Ouslati*** (TUN),		79
Faruke (SYR), **Jlot** (SYR), Korakoy (CYP), *Nasouhi Jaba* (JOR)	Faruke-Korakoy-Jlot ([3])	80
Chemlali Tataouine (TUN), ***Chemlali Zarzis*** (TUN),		81
Asi Yeli (TUR), **Memecik** (TUR), Tasarasi (Aydin) (TUR), Tasarasi (Kusadasi) (TUR), Yerli Yaglik (TUR)	Asi Yeli-Yerli Yaglik-Memecik ([1]; [42]; [46]; [99]), Tasarasi ([1]; [42]; [46])	82
Halhali (Hatay) (TUR), *Kalembezi* (TUR), *Kilis Yaglik* (TUR), Sayfi-2325 (TUR), *Tesbih Celebi* (TUR), Yaglik Sari Zeytin (TUR), ***Zaity*** (SYR),	Halhali (Hatay)-Sayfi-Yaglik Sari Zeytin ([42])	83
Baladi (Fekhe) (LBN), ***Saifi*** (SYR),		84
Trylia (SYR), *Ulliri i Zi* (ALB),		85

Accesions/cultivars included in each synonymy group	Previous bibliographic reports of synonymy	Sinonymy Group
Abou Choki-1126 (SYR), *Arabi Altafila* (JOR), Aredhioy_2 (CYP), Athalassa (CYP), Ayitrimihias (CYP), *Ayrouni Amioun* (LBN), *Ayrouni-2077* (LBN), Bakuri (SYR), *Baladi (Ain Baal)* (LBN), *Baladi (Aitaroun)* (LBN), *Baladi (Koura)* (LBN), *Baladi Deir Aamass* (LBN), *Baladi Deir Memass (LBN), Baladi Hasrout* (LBN), *Baladi Janata (LBN), Baladi Jowaya (Qana)* (LBN), *Baladi Kfarzaina* (LBN), *Baladi (Qana)* (LBN), *Baladi Zgharta* (LBN), **Baladi** (LBN), Flasou (CYP), *Karamani-2326* (TUR), *Ketat* (JOR), *Kfari Beladi* (JOR), *Kfari Romi* (JOR), *Khodeiri* (SYR), *Kucuk Topak Ulak* (TUR), *Ladoelia* (CYP), Lagoydhera (CYP), *Nabali Baladi* (JOR), *Remmani-2081* (LBN), Roumani (Qana) (LBN), *Sabbighi* (LBN), *Saurani* (TUR), Sourani-1478 (SYR), Souri-1843 (ISR)	Abou Choki-1126 ([6]; [16]; [24]), Aredhioy_2 ([3]; [96]), Athalassa ([3]; [6]; [16]; [24]), Ayitrimihias ([3]; [96]), Bakuri ([3]), Baladi ([6]; [97]); Flasou ([96]), Lagoydhera ([3]; [96]), Roumani (Qana) ([3]), Sourani - Souri ([24])	86
Bouchoukra (DZA), *Derdi* (DZA)		87
**Belluti** (TUR), *Hirhali Celebi* (TUR), Yun Celebi (TUR)	Yun Celebi-Belluti ([6]; [16]; [24])	88
Farga (ESP), *Solimar* (ESP)		89
**Grappolo** (ITA), Leccio del Corno (ITA), Marzio (ITA),	Leccio del Corno ([3]; [17]), Marzio ([3])	90
Evrihoy_1 (CYP), Flasoy (CYP), **Kato Drys** (CYP), Klirou (CYP)	Evrihoy_1 ([3]; [100]), Flasoy ([3]; [100]), Klirou ([3]; [6]; [16]; [96])	91
**Buga** (HRV), Crnica-1976 (HRV), *Crnjaka* (MNE),	Crnica-Buga ([3])	92
Erdek Yaglik (TUR), **Gemlik** (TUR), Kfarzeta (SYR), Samsun Ufak Tuzlamalik (TUR), *Sinop no 2* (TUR), Siyah Salamuralik (Tekirdag) (TUR), *Sukare* (SYR)	Erdek Yaglik ([3]; [6]; [16]; [24]; [42]), Kfarzeta ([3]), Samsun Ufak Tuzlamalik ([3]; [6]); Siyah Salamuralik (Tekirdag)([42])	93
Cañivano Negro-55 (ESP), *Zsb2* (MAR)		94
Baladi Ijd Ibrine (LBN), *Baladi Mabroum* (LBN), *Baladi Roumani* (LBN), *Bissani* (LBN), Doebli-1596 (SYR), Drmlali (SYR), Remmani-1762 (LBN), **Toffahi-1000** (SYR),	Doebli-Drmlali ([1]), Remmani-Drmlali ([3])	95
**Djabali Kini-1572** (SYR), Killin (SYR)	([3])	96
Shame-1620 (SYR), ***Shami Modabl*** (SYR)	([1])	97
Hembalassi (SYR), ***Shame*** (SYR)		98

**Accesions/cultivars included in each synonymy group**	**Previous bibliographic reports of synonymy**	**Sinonymy Group**
Abadi Shlal (SYR), Abbadi (**SYR), Abbadi Shalal** (SYR)	Abbadi-Abbadi Shalal ([3]; [6])	99
Habichuelero de Grazalema (ESP), *Hiloso* (ESP)		100
**Alameño de Cabra** (ESP), Negrillo de Estepa (ESP), Negro de Aguilar (ESP)	Negrillo de Estepa ([16]; [24])	101
Ljubuški Stari Grad (BiH), **Oblica** (HRV), *Studenci* (BiH)		102
**Picudo** (ESP), Picudo de Fruto Rojo (ESP)	([6]; [16]; [24]; [42])	103
Llorón de Ronda (ESP), **Morona** (ESP)	([3]; [6])	104
Lianolia Kerkyras (GRC), *Ulliri i Holle i Himares* (ALB)		105
**Alfafara** (ESP), Royal de Villena (ESP)	([3]; [6])	106
Azeitera (PRT), *Azulejo* (ESP), **Manzanilla Cacereñ**a (ESP), Negrinha (PRT)	Azeitera ([3]; [6]; [17]; [81]), Negrinha ([3]; [6]; [17]; [81])	107
Hamza Celebi (TUR), ***Kan Celebi*** *(TUR)*		108
Castellana (ESP), *Racimo* (ESP)		109
Manzanilla de Requena (ESP), Meloncillo (ESP), **Mollar de Cieza** (ESP), Ojua (ESP), Verdalón (ESP)	Manzanilla de Requena ([42]), Meloncillo-Ojua-Verdalón ([3]; [6]; [16])	110
Pecoso (ESP), **Pico Limón** (ESP)	([6]; [24])	111
Chemlal (DZA), **Chemlal de Kabilye** (DZA)	([92])	112
Palomillo (ESP), **Rechino** (ESP),	([6])	113
Belgentieroise (FRA), ***Merhavia*** (ISR)		114
Cerezuela (ESP), **Morejona** (ESP)	([3]; [6]; [16]; [24])	115
**Nevado Rizado** (ESP), Torcio de Huelma (ESP)	([6])	116
Branquita de Elvas Red (PRT), **Villalonga** (ESP)	([3]; [6]; [81])	117
Escarabajuelo de Ubeda (ESP), **Jabaluna** (ESP)	([16])	118
Galega Vulgar (PRT), *Getsemani* (MEX)		119
**Empeltre** (ESP), Grosal de Albocácer (ESP)	([101])	120
**Kalokerida** (GRC), Oliviere (FRA)	([3])	121
**Mixani** (ALB), Ulliri i Bardhe Berat (ALB)	([6])	122
Abbadi Abou Gabra-1033 (SYR), **Bent al Kadi** (SYR)	([6]; [24])	123
Janude-2 (SYR), **Karamani** (SYR)	Janude-2 ([3])	124
**Masabi** (SYR), Mossabi (SYR), *Shami-2259* (JOR)	Masabi-Mossabi ([1])	125

Accesions/cultivars included in each synonymy group	Previous bibliographic reports of synonymy	Sinonymy Group
Ansasi (SYR), ***Ensasi*** (SYR)		126
Cayet Roux (FRA), ***Mission Moojeski*** (USA)		127
Aggizi Akse (EGY), *Agizi Aas* (EGY)		128
Cayon (FRA), *Rougette de Metidja* (DZA)		129
Gjykatesi (ALB), ***Kokerrmadh Elbasani*** (ALB), *Krypsi i Krujes* (ALB)		130
**Longue de Meliana** (DZA), Ronde de Meliana (DZA)	([3])	131
Hanefa (SYR), ***Zada*** (SYR)		132
**Abo Anaked** (SYR), Om Kanane (SYR)	([84])	133
Buyuk Topak Ulak (TUR), *Cilli* (TUR),		134
Hendero (ESP), *Olivo de Nueva Carteya* (ESP)		135
Carrasqueta De Ayora (ESP), *Tempranillo de Ayora* (ESP)		136
Eşek Zeytini (Tekirdag) (TUR), ***Thassitiki*** (GRC)		137
Brava (ESP), *Desconocida Vx1* (ESP)		138
Celebi (Iznik) (TUR), *Samsun Kirmizi Tuzlamalik* (TUR)		139
**Halhali Celebi** (TUR), Yuvarlak Halhali (TUR)	([99])	140

* In italics are indicated new observed synonymy cases

** In bold are included the representative cultivars at WOGBC of each synonymy group
