# Supplementary material for: Utility of EST-SNP Markers for Improving Management and Use of Olive Genetic Resources: A Case Study at the Worldwide Olive Germplasm Bank of Córdoba
Source: Plants (Basel). 2022 Mar 29;11(7):921. doi: 10.3390/plants11070921 (PMC9002360; doi:10.3390/plants11070921)
Supplement: Supplementary file 1 [file plants-11-00921-s001.zip › Table S4_revised.pdf]

Supplemental Table S4: Cultivars included in each homonymy group considered in the present study. General denomination of each homonymy group, possible meaning of homonyms´ denomination and related bibliographic references are also indicated.

Homonymy Group	Accesions/cultivars included in each homonymy group	Origin*	General denomination of homonymy groups	General meaning of homonymy´s denomination	Bibliographic reference
1	Abade Helo-1623	SYR	Abadi / e / Abbadi / Abbadi Abou Gabra	Black fruit	[3], [4], [6], [24], [102]
	Abade Mour	SYR			
	Abadi Rassasi	SYR			
	Abbadi	SYR			
	Abbadi Shalal	SYR			
	Abbadi Abou Gabra-1033	SYR			
	Abbadi Abou Gabra-842	SYR			
2	Abo Akfa	SYR	Abo / Abou	Fruits having a certain trait or shape	[3], [6], [24], [102]
	Abo Anaked	SYR			
	Abou Satl Mohazam	SYR			
	Abou Kanani	SYR			
3	Abo Shawke	SYR	Abo Shawke / Abou Chawkeh / Abou Choki / Bouchouk	Fruits or stones with a thorn	[3], [6], [43]
	Abou Chawkeh	LBN			
	Abou Choki-1115	SYR			
	Abou Choki-1126	SYR			
	Bouchouk Lafayette	DZA			
	Bouchouk Laghlid	MAR			
4	Acebuche	ESP	Acebuche / Acebuchera / Azeboudi / Zeboudj	Wild olive	
	Acebuche de Caravaca-800	ESP			
	Acebuchera	ESP			
	Azeboudi de Kirane-1549	DZA			
	Azeboudi de Kirane-1659	DZA			
	Zeboudj Boudoudan	DZA			

Homonymy Group	Accesions/cultivars included in each homonymy group	Origin*	General denomination of homonymy groups	General meaning of homonymy´s denomination	Bibliographic reference
5	Aceitunero-2022	ESP	Aceitunero / Zeitoun / Zeitun / Zeitini / Zeytin	Olive / olive farmer	[46]
	Aceitunero-2249	ESP			
	Zeitoun Boubezzoula	TUN			
	Zeitun lkhl	MAR			
	Zitoune DK	MAR			
	Girit Zeytini	TUR			
	Ak Zeytin	TUR			
6	Aggezi	EGY	Aggezi / Aggizi	Old or sacred	[3]
	Aggizi Akse	EGY			
	Aggizi Oshime	EGY			
	Aggizi Shame	EGY			
7	Aimel	DZA	Aimel	From Aimel (toponymy)	
	Aimel (Setif)	DZA			
8	Alameño Blanco	ESP	Alameño	Erect like an aspen tree	[3], [4], [6], [28], [42]
	Alameño de Cabra	ESP			
	Alameño de Marchena	ESP			
	Alameño de Montilla	ESP			
9	Aloreña de Iznalloz	ESP	Aloreña	From Álora (toponymy)	
	Aloreña	ESP			
	Aloreña-829	ARG			
10	Arabi Altafeleh	JOR	Arabi / Arabi Altafila	Arabic	
	Arabi Altafila	JOR			
11	Ascolana Huasco	CHL	Ascolana	From Ascoli (toponymy)	
	Ascolana Tenera	ITA			
12	Ayrouni	LBN	Ayrouni	Very small fruits	
	Ayrouni Amioun	LBN			
	Ayrouni-1599	SYR			
	Ayrouni-2077	LBN			

Homonymy Group	Accesions/cultivars included in each homonymy group	Origin*	General denomination of homonymy groups	General meaning of homonymy´s denomination	Bibliographic reference
13	Baid El Hamam<br>Bidh Hmam<br>Bed al Igel	EGY<br>TUN<br>SYR	Baid / Bidh / Bed	Big like an egg	
14	Baladi (Ain)<br>Baladi<br>Baladi (Fekhe)<br>Baladi Ijd Ibrine<br>Baladi Mabroum<br>Baladi Roumani<br>Baladi Tawil<br>Balady<br>Beldi	LBN<br>LBN<br>LBN<br>LBN<br>LBN<br>LBN<br>LBN<br>EGY<br>TUN	Baladi / Balady / Beldi	Local / from the country	[33]
15	Baladi Hermel-2072<br>Baladi Hermel-2074	LBN<br>LBN	Baladi / Baladi Hermel	Local from Hermel (toponymy)	
16	Baladi Remadieh-1769<br>Baladi Remadieh-2082	LBN<br>LBN	Baladi / Baladi Remadieh	Local from Remadieh (toponymy)	
17	Balhi Sig<br>Balah	TUN<br>LBN	Balah / Balhi	Date fruit shape	
18	Barouni-1661<br>Barouni-1823	TUN<br>TUN	Barouni	High-class	
19	Barri<br>Berri Meslal 3	SYR<br>MAR	Barri / Berri	Wild olive	
20	Bianchera<br>Biancolilla<br>Biancolilla-1814<br>Blanqueta<br>Blanqueta Gorda<br>Blanquette de Gastu<br>Blanquette de Guelma<br>Branquita de Elvas Red	ITA<br>ITA<br>ARG<br>ESP<br>ESP<br>DZA<br>DZA<br>PRT	Bianchera / Biancolilla / Blanquet a / tte / Branquita	White olive	[92]

Homonymy Group	Accesions/cultivars included in each homonymy group	Origin*	General denomination of homonymy groups	General meaning of homonymy´s denomination	Bibliographic reference
21	Bjelica	HRV	Bjelica	White olive	[103]
	Istarska Bjelica	HRV			
	Istrka Belica	SVN			
	Plementa Bjelica	HRV			
22	Cañivano Blanco	ESP	Cañivano	Wood (black or white)	[3], [4], [6], [28], [42]
	Cañivano Negro	ESP			
	Cañivano Negro-55	ESP			
23	Cariasina	ITA	Cariasina / Cerasuola / Cerezuela / Cerisier / Ciliegino / Kiraz	Cherry fruit shape or colour	
	Cerasuola	ITA			
	Cerezuela	ESP			
	Cerisier	FRA			
	Ciliegino	ITA			
	Kiraz	TUR			
24	Carrasco	ESP	Carrarsco / Carrasqueño (a) / Carrasqueta / Carrasquillo	Robust leaves like holm oak and oak	[3], [4], [6], [42]
	Carrasqueña de Cañada	ESP			
	Carrasqueña Huasco	CHL			
	Carrasqueño de Alburquerque	ESP			
	Carrasqueño de Alcaudete	ESP			
	Carrasqueño de Elvas	PRT			
	Carrasqueño de Jumilla	ESP			
	Carrasqueño de la Sierra	ESP			
	Carrasqueño de Porcuna	ESP			
	Carrasquillo	ESP			
	Carrasqueta De Ayora	ESP			
25	Cayet Noir	FRA	Cayet	Mottled fruits	
	Cayet Roux	FRA			

Homonymy Group	Accesions/cultivars included in each homonymy group	Origin*	General denomination of homonymy groups	General meaning of homonymy´s denomination	Bibliographic reference
26	Hamza celebi	TUR	Celebi	High status / big size of the fruit	[21]
	Kan Celebi	TUR			
	Tesbih Celebi	TUR			
	Yun Celebi	TUR			
	Celebi (Iznik)	TUR			
	Celebi (Silifke)	TUR			
	Hirhali Celebi	TUR			
27	Cerruda de Liesa	ESP	Cerruda	Closed and densed branches	[95], [101]
	Cerruda de Olvena	ESP			
28	Chemlal	DZA	Chemlal / i	Small fruits	[41], [92], [93]
	Chemlal de Kabilye	DZA			
	Chemlali Balhi	TUN			
	Chemlali Bent Louzir	TUN			
	Chemlali Ghraiba	TUN			
	Chemlali Mahares	TUN			
	Chemlali Meliane	TUN			
	Chemlali Nord	TUN			
	Chemlali Ouled Msallem	TUN			
	Chemlali Sfax	TUN			
	Chemlali Sig	TUN			
	Chemlali Tataouine	TUN			
	Chemlali Zarzis	TUN			
29	Chemlali Ontha Tataouine	TUN	Antha / Ontha	Female / fertile, high prodcuctive tree	[52], [92], [93]
	Chaïbi Antha	TUN			
30	Chorreao de Montefrío	ESP	Chorreao / Chorrúo	Weeping tree (branches)	[42]
	Chorrúo	ESP			
	Chorrúo de Castro del Río-361	ESP			

Homonymy Group	Accesions/cultivars included in each homonymy group	Origin*	General denomination of homonymy groups	General meaning of homonymy´s denomination	Bibliographic reference
31	Cirujal<br>Cirujal-2027<br>Cirujal-2153	ESP<br>ESP<br>ESP	Cirujal	Plum fruit / tree shape	
32	Cordobés de Arroyo de la Luz<br>Cordobés de la Aliseda<br>Cordovil de Castelo Branco<br>Cordovil de Serpa	ESP<br>ESP<br>PRT<br>PRT	Cordobés / Cordovil	From Córdoba (toponymy)	[3], [4], [6], [24], [42]
33	Cornicabra<br>Cornicabra de Jerez Caballeros<br>Cornicabra de Mérida<br>Cornicabra Murciana<br>Cornezuelo de Jaén	ESP<br>ESP<br>ESP<br>ESP<br>ESP	Cornicabra / Cornezuelo	Long curved fruits (like horns)	[3], [4], [6], [24], [42]
34	Corscicana da Mensa<br>Corsicana da Olio	ITA<br>ITA	Corsicana	From Corse Island (toponymy)	[1], [83]
35	Crnica<br>Crnjaka	HRV<br>MNE	Crnica / Crnjaka	Black fruits	[103]
36	Cuquello de la Jana<br>Cuquello-1	ESP<br>ESP	Cuquello	Bird (sweet for birds)	[101]
37	Dan<br>Dan-136	SYR<br>SYR	Dan	Jewel / gem	
38	Datilera de Caudiel<br>Datilero	ESP<br>ESP	Datilero / a	Date fruit shape	
39	Desconocida Vx1<br>Desconocida-1481<br>Desconocido-2021<br>Desconocido-2026	ESP<br>ESP<br>ESP<br>ESP	Desconocida	Uknown	
40	Dhokkar Nafti<br>Dhokkar Tataouine<br>Dokkar	TUN<br>TUN<br>TUN	Dhokkar / Dokkar	Male tree (pollinator)	[41]

Homonymy Group	Accesions/cultivars included in each homonymy group	Origin*	General denomination of homonymy groups	General meaning of homonymy´s denomination	Bibliographic reference
41	Djabali Kini-1572	SYR	Djabali	Mountainous	
	Djabali Kini-2057	SYR			
	Jabali	SYR			
42	Doebli	SYR	Doebli	Big and round	
	Doebli-1596	SYR			
43	Dolce	EGY	Dolce / Dolça / Dulzal	Sweet	[3], [4], [42]
	Dolce Agogia	ITA			
	Dolça	ESP			
	Dulzal	ESP			
	Dulzal de Carmona	ESP			
44	Dopia Pierias	GRC	Dopia	Local / from the country	[44]
	Dopia Verias	GRC			
45	Egriburum (Nizip)	TUR	Egriburum	Crooked nose	[21]
	Egriburum (Tatayn)	TUR			
46	Escarabajillo	ESP	Escarabaj(o) / (illo) / Escarabajuelo	Little beetle / beetle	[3], [4], [6]
	Escarabajo	ESP			
	Escarabajuelo de Atarfe	ESP			
	Escarabajuelo de Posadas	ESP			
	Escarabajuelo de Ubeda	ESP			
47	Esek Zeytini (Odemis)	TUR	Eşek zeytini / Zeytini	Donkey i.e. big olive fruit	[21]
	Esek Zeytini (Tekirdag)	TUR			
48	Gerboua	TUN	Gerboui / Gerboua	Family name in Tunisia (well known)	
	Gerboui	TUN			
	Gerboui-1551	TUN			
	Gerboui-1672	TUN			
	Gerboui-1862	TUN			

Homonymy Group	Accesions/cultivars included in each homonymy group	Origin*	General denomination of homonymy groups	General meaning of homonymy´s denomination	Bibliographic reference
49	Gorda Limoncillo	ESP	Gorda / Gordal / (Grossal / Grossane / Grossa) / Grossolana	Large / big / huge fruit size	[3], [4], [6], [24], [42], [101]
	Gordal de Archidona	ESP			
	Gordal de Écija	ESP			
	Gordal de Granada	ESP			
	Gordal de Hellín	ESP			
	Gordal de Somontano	ESP			
	Gordal de Vélez Rubio	ESP			
	Gordal Sevillana	ESP			
	Gordal Valle las Palmeras	MEX			
	Grosal de Albocácer	ESP			
	Grosal de Cieza	ESP			
	Grosal Vimbodí	ESP			
	Grossa di Spagna	ITA			
	Grossanne	FRA			
	Grossanne-67	FRA			
	Grossolana	ITA			
50	Gremignolo	ITA	Gremignolo		[3]
	Gremignolo di Bolgheri	ITA			
51	Habichuelero de Baena	ESP	Habichuelero	Small bean	[3], [6], [42]
	Habichuelero de Grazalema	ESP			
52	Halhali (Hatay)	TUR	Halhali / Khalkhali	Pending like anklets / or aboundant	[46], [102]
	Halhali Celebi	TUR			
	Khalkhali	SYR			
	Khalkhali-1107	SYR			
	Khalkhali-1595	SYR			
	Khalkhali-789	SYR			
53	Hamra	DZA	Hamra / Hamrani	Red colour	
	Hamrani	MAR			

Homonymy Group	Accesions/cultivars included in each homonymy group	Origin*	General denomination of homonymy groups	General meaning of homonymy´s denomination	Bibliographic reference
54	Hembalassi<br>Hemblasi	SYR<br>SYR	Hembalassi / Hemblasi	Myrtle / myrtle fruit shape	
55	Hojiblanca<br>Hojiblanca de Navarrés	ESP<br>ESP	Hojiblanca	White leaf	[42], [101]
56	Hurma Kaba<br>Hurma Karaca	TUR<br>TUR	Hurma	Date fruit shape	[46], [104]
57	Imperial<br>Imperial de Jaén	ESP<br>ESP	Imperial	Imperial / magnificient	[4]
58	Injassi Gafsa<br>Injassi Hchichina	TUN<br>TUN	Injassi	Pear fruit shape	[41]
59	Jaén-1<br>Jaén-2	ESP<br>ESP	Jaén	From Jaén (toponymy)	
60	Janude-1<br>Janude-2	SYR<br>SYR	Janude		
61	Jemri Bouchouka<br>Jemri Dhokkar-1732<br>Jemri Dhokkar-1907	TUN<br>TUN<br>TUN	Jermri / Jemri Dhokkar	Red as a burning coal	[41]
62	Jlot<br>Jlot-1965<br>Jlot-841<br>Jlot-976	SYR<br>LBN<br>SYR<br>SYR	Jlot	Big	[3], [6]
63	Kalb-el-tair<br>Kelb et Ter-144<br>Kelb et Ter-145	LBN<br>SYR<br>SYR	Kalb / Kelb / et tair / ter	Heart of the bird	[4]
64	Kallmet<br>Kallmet Kokerrvogel	ALB<br>ALB	Kallmet	From Kallmet (toponymy)	[105], [106]

Homonymy Group	Accesions/cultivars included in each homonymy group	Origin*	General denomination of homonymy groups	General meaning of homonymy´s denomination	Bibliographic reference
65	Karamani<br>Karamani-1564<br>Karamani-2326	SYR<br>SYR<br>TUR	Karamani	Generous	
66	Kfari Beladi<br>Kfari Romi<br>Kfarzeta	JOR<br>JOR<br>SYR	Kfar(i)	Oily	
67	Klon-14-1081<br>Klon-14-1812	ALB<br>ALB	Klon	Clonal	[18]
68	Kokerrmadh Elbasani<br>Kokerrmadhi i Beratit	ALB<br>ALB	Kokerrmadh	Big / large fruit	[105], [106]
69	Leccio del Corno<br>Leccio Maremmano<br>Leccione	ITA<br>ITA<br>ITA	Leccio / Leccione	Oak (little or big oak)	[3]
70	Lechín de Granada<br>Lechín de Sevilla	ESP<br>ESP	Lechín	White flesh olive fruits	[3], [6], [24], [28], [42]
71	Limoncillo<br>Llimonenca<br>Pico Limón<br>Pico Limón de Grazalema	ESP<br>ESP<br>ESP<br>ESP	Limoncillo / Llimonenca / Pico Limón	Lemon fruit shape / nipple like lemon	[6], [3]
72	Llorón de Atarfe<br>Llorón de Iznalloz<br>Llorón de Ronda	ESP<br>ESP<br>ESP	Llorón	Weeping tree (branches)	[3], [6], [42]
73	Macho de Jaén<br>Machorron<br>Olivo Macho de Santisteban Pto	ESP<br>ESP<br>ESP	Macho / Machorron / Olivo Macho	Male olive tree (pollinator)	
74	Mahati-1010<br>Mahati-1581<br>Mahati-846	SYR<br>SYR<br>SYR	Mahati	Big black fruits (like the eyes of a cow)	[3], [6], [102]

Homonymy Group	Accesions/cultivars included in each homonymy group	Origin*	General denomination of homonymy groups	General meaning of homonymy´s denomination	Bibliographic reference
75	Majhol-1059<br>Majhol-1063<br>Majhol-1122<br>Majhol-152	SYR<br>SYR<br>SYR<br>SYR	Majhol	Unknown	[3], [4], [6], [24], [102]
76	Manzanil de Piedra Buena-489<br>Manzanilla Aceitera<br>Manzanilla Cacereña<br>Manzanilla Chilena<br>Manzanilla de Abla<br>Manzanilla de Agua<br>Manzanilla de Almería<br>Manzanilla de Ayora<br>Manzanilla de Caudiel<br>Manzanilla de Hellín<br>Manzanilla de Jaén<br>Manzanilla de Montefrío<br>Manzanilla de Requena<br>Manzanilla de San Vicente<br>Manzanilla de Sevilla<br>Manzanilla de Tortosa<br>Manzanilla del Centro<br>Manzanilla del Piquito<br>Manzanilla Picua<br>Manzanilla Prieta<br>Manzanillera de Huércal Overa<br>Manzanillo Cordobés de Cabra	ESP<br>CHL<br>ESP<br>CHL<br>ESP<br>ESP<br>ESP<br>ESP<br>ESP<br>ESP<br>ESP<br>ESP<br>ESP<br>MEX<br>ESP<br>ESP<br>ESP<br>ESP<br>ESP<br>ESP<br>ESP<br>ESP	Manzanil / Manzanilla / o / Manzanillera	Apple fruit shape	[3], [4], [6], [17], [24], [28], [42]
77	Marsaline<br>Marsaline-1509<br>Marsaline-1684	TUN<br>TUN<br>TUN	Marsaline		[41]

Homonymy Group	Accesions/cultivars included in each homonymy group	Origin*	General denomination of homonymy groups	General meaning of homonymy´s denomination	Bibliographic reference
78	Mawi<br>Mawi Stanboli	SYR<br>SYR	Mawi	High vigor	
79	Meski<br>Meski Zarzis	TUN<br>TUN	Meski	Perfumed / scented	[93]
80	Mignolo<br>Mignolo Cerretano	ITA<br>ITA	Mignolo	Small fruit / the name of the inflorescence of the olive (mignola)	[3]
81	Misión de San Vicente<br>Mission Moojeski<br>Mission Nieland	MEX<br>USA<br>USA	Misión / Mission	Mission (religious)	[3], [6]
82	Mollar Basto<br>Mollar de Cieza	ESP<br>ESP	Mollar	Soft pulp of the fruit	[3], [4]
83	Moresca<br>Morisca<br>Morisca de Mancor	ITA<br>ESP<br>ESP	Moresca / Morisca	Dark or black fruit	[3], [4], [6], [42]
84	Morona<br>Morona de Castellon	ESP<br>ESP	Morona		[42], [101]
85	Morruda de Salinas<br>Morruda de Segorbe<br>Morrut<br>Morrut-607	ESP<br>ESP<br>ESP<br>ESP	Morruda / Morrut	Beak shape	[101], [107]
86	Nabali<br>Nabali Baladi<br>Nabali Muhasan	ISR<br>JOR<br>JOR	Nabali	Local from Beit Nabala (toponymy) / arrow shaped	
87	Neb Djemel<br>Neb Tataouine	DZA<br>TUN	Neb	Tooth / fang of the camel	[41]

Homonymy Group	Accesions/cultivars included in each homonymy group	Origin*	General denomination of homonymy groups	General meaning of homonymy´s denomination	Bibliographic reference
88	Negral de Bierge	ESP	Negral / Negrillo / Negro / Nera / Noirette	Black fruits / early ripening	[29], [42]
	Negral de Sabiñán	ESP			
	Negrillo de Arjona	ESP			
	Negrillo de Estepa	ESP			
	Negrillo de Iznalloz	ESP			
	Negrillo de la Carlota	ESP			
	Negrillo Redondo	ESP			
	Negro del Carpio	ESP			
	Negro de Aguilar	ESP			
	Nera di Gonnos	ITA			
	Nera di Oliena	ITA			
	Noirette de l´Herault	FRA			
89	Nerba	ITA	Nerba	Suitable for whipping or shaking (tree)	
	Nerba Catanese	ITA			
90	Nevadillo de San Vicente	MEX	Nevadillo / Nevado	Snowy / white colour (dust) of the fruit and / or leaves	[3], [4], [42]
	Nevadillo de Santisteban Pto	ESP			
	Nevadillo Valle las Palmeras	MEX			
	Nevadillo Blanco de Jaén	ESP			
	Nevadillo Blanco de Lucena	ESP			
	Nevadillo Blanco de Osuna	ESP			
	Nevadillo Negro de Jaén	ESP			
	Nevadillo Negro-2282	ESP			
	Nevado Basto	ESP			
	Nevado Basto de Zuheros	ESP			
	Nevado Rizado	ESP			
	Nevado Azul	ESP			
91	Nocellara del Belice	ITA	Nocellara / Nociara	Walnut	[29]
	Nocellara Messinese	ITA			
	Nociara	ITA			
92	Ocal de Alburquerque	ESP	Ocal	Hollow fruit (sweet and delicate)	[4], [6]
	Ocal-25	ESP			
	Ocal	ESP			

Homonymy Group	Accesions/cultivars included in each homonymy group	Origin*	General denomination of homonymy groups	General meaning of homonymy´s denomination	Bibliographic reference
93	Ogliarola del Bradano	ITA	Ogliarola	Oil / oily	[3], [29]
	Ogliarola del Vulture	ITA			
94	Olivastra di Populonia	ITA	Olivastra	Wild olive	[3], [29]
	Olivastra Seggianese	ITA			
95	Olivo da Mensa	ITA	Olivo	Olive	[3], [29], [91]
	Olivo de Aguamarga	ESP			
	Olivo de Galicia	ESP			
	Olivo de Mancha Real	ESP			
	Olivo de Maura	ESP			
	Olivo de Nueva Carteya	ESP			
	Olivo del Mulino	ITA			
	Olivo di Casavecchia	ITA			
	Olivo di Castiglione	ITA			
	Olivo di Mandanici	ITA			
	Olivo di San Lorenzo	ITA			
	Olivos de Cotillas	ESP			
	Olivos de El Barranco del Lobo	ESP			
96	Oueslati T-1919	TUN	Ouslati / Oueslati	From Ouslati region (toponymy)	
	Ouslati	TUN			
97	Pajarera	ESP	Pajarera / o	Birds' nest (sweet fruits preferred by the birds)	[42]
	Pajarero	ESP			
98	Palomar	ESP	Palomar / Palomillo	Pigeon nest / dovecote	[42]
	Palomillo	ESP			
99	Pendolino	ITA		Weeping tree (branches)	
	Penjoll	ESP	Pendolino / Penjoll		
100	Picholine	FRA	Picholine	Small thorny fruits	[3], [6], [24]
	Picholine Marocaine	MAR			
	Picholine-1091	FRA			

Homonymy Group	Accesions/cultivars included in each homonymy group	Origin*	General denomination of homonymy groups	General meaning of homonymy´s denomination	Bibliographic reference
101	Picual de Almería	ESP	Picual	Pointed shape of fruits	[3], [4], [6], [28], [42]
	Picual de Estepa	ESP			
	Picual	ESP			
102	Picuda de Luis	ESP	Picudo / Picuda	Fruits with curved apex / nippled	[3], [4], [6], [28], [42], [101]
	Picudo	ESP			
	Picudo Blanco de Estepa	ESP			
	Picudo de Fruto Rojo	ESP			
	Picudo de la Axerquía	ESP			
	Picudo de la Rioja	ESP			
	Picudo de Labata	ESP			
	Picudo de Montoro	ESP			
103	Pizz'e Carroga	ITA	Pizzè / Pizzo	Crow's beak	[1], [29]
	Pizzo di Corvo	ITA			
104	Racimal	ESP	Racimo / Racimal	Fructification in bunch like grapes	[42]
	Racimal de Jaén	ESP			
	Racimo	ESP			
105	Redondil	PRT	Redondil / Redondilla / Ronde / Tonda / Tondello	Round shaped fruits	[3], [42]
	Redondilla de Grazalema	ESP			
	Redondilla de Logroño	ESP			
	Ronde de la Ménara	MAR			
	Ronde de Meliana	DZA			
	Tonda di Cagliari	ITA			
	Tonda di Villacidro	ITA			
	Tonda Iblea	ITA			
	Tondello	ITA			
106	Remmani-1762	LBN	Remmani	Pomegrenate	
	Remmani-2081	LBN			

Homonymy Group	Accesions/cultivars included in each homonymy group	Origin*	General denomination of homonymy groups	General meaning of homonymy´s denomination	Bibliographic reference
107	Rojal	ESP	Rojal / Rossellino / Rosciola / Rosino / Rosselo / Rougette / Royal / Royeta / Royuela	Reddish / red fruit	[3], [29], [42], [79], [95]
	Rojal de Valencia	ESP			
	Rosciola	ITA			
	Rosino	ITA			
	Rossellino	ITA			
	Rossello	ITA			
	Rougette de l'Ardèche	FRA			
	Rougette de Metidja	DZA			
	Rougette de Pignans	FRA			
	Royal	ESP			
	Royal de Belchite	ESP			
	Royal de Calatayud	ESP			
	Royal de Calatayud-47	ESP			
	Royal de Cazorla	ESP			
	Royal de Sabiñán	ESP			
	Royeta de Abizanda	ESP			
	Royeta de Asque	ESP			
	Royuela	ESP			
108	Rosai-1809	JOR	Rosai	Hitting for preparing olives for table olives	
	Rosai-2254	JOR			
109	Safrawi	SYR	Safrawi	Yellow fruis	[3], [6]
	Safrawi-1116	SYR			
110	Saifi	SYR	Saifi / Sayfi	Summer / summerly / early bearing	
	Sayfi	SYR			
	Sayfi-2325	TUR			
111	Samsun Kirmizi Tuzlamalik	TUR	Samsun / Tuzlamalik	From Samsun (toponymy) / Salted (fruits)	[46]
	Samsun Tuzlamalik	TUR			
	Samsun Ufak Tuzlamalik	TUR			

Homonymy Group	Accesions/cultivars included in each homonymy group	Origin*	General denomination of homonymy groups	General meaning of homonymy´s denomination	Bibliographic reference
112	Samsun Salamuralik	TUR	Salamuralik	Brined (fruits)	[46]
	Siyah Salamuralik (Tekirdag)	TUR			
113	San Pedro	CHL	San / Sant(a)	Saint	[1], [29]
	Sant' Agostino	ITA			
	San Francesco	ITA			
	San Lazzero	ITA			
	Santa Caterina	ITA			
	Santa Martinenga	ITA			
114	Sari Hasebi (Hatay)	TUR	Sari	Yellow fruits	[46]
	Sari ulak	TUR			
115	Sayali	TUN	Sayali	Oil	
	Sayali T-1677	TUN			
	Sayali T-1728	TUN			
116	Sevillana	ESP	Sevillano / Sevillana / Sevilenca / Sivigliano	From Sevilla (toponymy)	[1], [3], [83]
	Sevillana (Azapa)	CHL			
	Sevillano de Jumilla	ESP			
	Sevillenca	ESP			
	Sivigliana da Olio	ITA			
	Sivigliana da Mensa	ITA			
117	Shame	SYR	Shami / Shame	From Damasco (toponymy)	[3], [102]
	Shame-1620	SYR			
	Shami Modabl	SYR			
	Shami-1041	SYR			
	Shami-141	SYR			
	Shami-2259	JOR			
	Shami-953	SYR			
118	Shengeh Manjil	IRN	Shengeh		[47], [108]
	Shengeh Sur	IRN			

Homonymy Group	Accesions/cultivars included in each homonymy group	Origin*	General denomination of homonymy groups	General meaning of homonymy´s denomination	Bibliographic reference
119	Sinop<br>Sinop no 2<br>Sinop no 6	TUR<br>TUR<br>TUR	Sinop	From Sinop (toponymy)	[46]
120	Saurani<br>Sorani<br>Sorani-1566<br>Souri<br>Souri-1589<br>Soury Lari	TUR<br>SYR<br>SYR<br>ISR<br>SYR<br>LBN	Saurani / Sorani / Souri	From Tyre / Sor (toponymy)	
121	Stanboli-991<br>Stanbouli	SYR<br>SYR	Stanbouli / Stanboli	From Istanbul (toponymy)	
122	Stara Maslina-1999<br>Stara Maslina-2005	MNE<br>HRV	Stara maslina	Old olive	
123	Tefah<br>Toffahi<br>Toffahi-1000<br>Toffehi Tataouine<br>Tufahi Tadmori	DZA<br>EGY<br>SYR<br>TUN<br>SYR	Tefah / Toffahi / Toffehi	Apple fruit shape	[3], [6], [24], [102]
124	Temprana de Montán<br>Tempranillo de Ayora<br>Tempranillo de Lucena<br>Tempranillo de Yeste<br>Temprano	ESP<br>ESP<br>ESP<br>ESP<br>ESP	Temprana / Tempranillo / Temprano	Early bearing or ripening	[4], [24], [42]
125	Torcío<br>Torcío de Cabra<br>Torcío de Huelma	ESP<br>ESP<br>ESP	Torcío	Crooked (olive tree)	[42]

Homonymy Group	Accesions/cultivars included in each homonymy group	Origin*	General denomination of homonymy groups	General meaning of homonymy´s denomination	Bibliographic reference
126	Ulliri i Holle i Himares	ALB	Ulliri	Olive	[105], [106]
	Ulliri Shekullor Berat	ALB			
	Ulliri i Kuq	ALB			
	Ulliri i Zi	ALB			
127	Ulliri i Bardhe Berat	ALB	Ulliri i Bardhe	White olive fruit	[105], [106]
	Ulliri i Bardhe i Tiranes	ALB			
	Ulliri i Bardhe Kruje	ALB			
128	Verdal de Alhama	ESP	Verde / Verdal / Verdala / Verdalón / Verdeña / Verdial / Verdiell / Verdilla	Green / Greenish	[3], [4], [6], [24], [28], [42]
	Verdal de Manresa	ESP			
	Verdala	ESP			
	Verdale	FRA			
	Verdalón	ESP			
	Verde Verdelho	PRT			
	Verdeña	ESP			
	Verdial de Badajoz	ESP			
	Verdial de Cádiz	ESP			
	Verdial de Huévar	ESP			
	Verdial de Montefrío	ESP			
	Verdial de Vélez-Málaga	ESP			
	Verdial de Vélez-Málaga-51	ESP			
	Verdiell	ESP			
	Verdilla de Calatayud	ESP			

Homonymy Group	Accesions/cultivars included in each homonymy group	Origin*	General denomination of homonymy groups	General meaning of homonymy´s denomination	Bibliographic reference
129	Yag Celebi (Tatayn)	TUR	Yag / Yaglik	Oil / oily	[46]
	Beyaz Yaglik	TUR			
	Erdek Yaglik	TUR			
	Kilis Yaglik	TUR			
	Samsun Yaglik	TUR			
	Yaglik Celebi	TUR			
	Yerli Yaglik	TUR			
	Edremit Yaglik	TUR			
	Yag Zeytini	TUR			
	Yaglik Sari Zeytin	TUR			
130	Yuvarlak Celebi	TUR	Yuvarlak	Round	[46]
	Yuvarlak Halhali	TUR			
	Iri Yuvarlak	TUR			
131	Zard	IRN	Zard	Yellow	
	Zard-1810	IRN			
132	Zarrazi	TUN	Zarrazi	Bitter taste (table olives)	[41]
	Zarrazi Douirat	TUN			
	Zarrazi Injassi	TUN			
	Zarrazi Zarzis	TUN			
	Zarrazyet Sned	TUN			

*ALB=Albania ; ARG=Argentina ; BiH=Bosnia and Herzegovina ; CYP=Cyprus ; CHL=Chile ; DZA=Algeria ; EGY=Egypt ; ESP=Spain ; FRA=France ; GRC=Greece ; HRV=Croatia ; IRN=Iran ; IRQ=Iraq ; ISR=Israel ; ITA=Italy ; JOR=Jordan ; LBN=Lebanon ; MAR=Morocco ; MEX=Mexico ; MNE=Montenegro ; PAK=Pakistan ; PER=Peru ; PRT=Portugal ; SVN=Slovenia ; SYR=Syria ; TUN=Tunisia ; TUR=Turkey ; URU=Uruguay ; USA=United States of America
