# Supplementary material for: Utility of EST-SNP Markers for Improving Management and Use of Olive Genetic Resources: A Case Study at the Worldwide Olive Germplasm Bank of Córdoba
Source: Plants (Basel). 2022 Mar 29;11(7):921. doi: 10.3390/plants11070921 (PMC9002360; doi:10.3390/plants11070921)
Supplement: Supplementary file 1 [file plants-11-00921-s001.zip › Table S5_revised.pdf]

**Supplemental Table S5**: Diversity parameters of the 96 EST-SNPs genotyped in the 668 non-redundant genotypes*

| Locus | N | $N_{avg}$ | $N_e$ | *I* | $H_o$ | $H_e$ | *MAF* | *PIC*** |
|---|---|---|---|---|---|---|---|---|
| **EContig10374** | 668 | 2,000 | 1,774 | 0,628 | 0,502 | 0,436 | 0,47231 | 0,341 |
| **EContig10531** | 668 | 2,000 | 1,994 | 0,692 | 0,523 | 0,498 | 0,25187 | 0,374 |
| **EContig10539** | 668 | 2,000 | 1,782 | 0,631 | 0,522 | 0,439 | 0,47754 | 0,343 |
| **EContig10640** | 666 | 2,000 | 1,812 | 0,640 | 0,495 | 0,448 | 0,31811 | 0,348 |
| **EContig10787_1** | 668 | 2,000 | 1,800 | 0,637 | 0,499 | 0,444 | 0,38323 | 0,346 |
| **EContig1180_2** | 664 | 2,000 | 1,894 | 0,665 | 0,478 | 0,472 | 0,43413 | 0,361 |
| **EContig12203_1** | 630 | 2,000 | 1,764 | 0,625 | 0,352 | 0,433 | 0,38906 | 0,339 |
| **EContig12584** | 667 | 2,000 | 1,997 | 0,693 | 0,563 | 0,499 | 0,32111 | 0,375 |
| **EContig1263** | 666 | 2,000 | 1,734 | 0,615 | 0,462 | 0,423 | 0,3735 | 0,334 |
| **EContig1372_4** | 666 | 2,000 | 1,633 | 0,576 | 0,445 | 0,388 | 0,45502 | 0,313 |
| **EContig1634** | 667 | 2,000 | 1,728 | 0,612 | 0,463 | 0,421 | 0,42748 | 0,333 |
| **EContig1775_5** | 662 | 2,000 | 1,679 | 0,594 | 0,463 | 0,404 | 0,4753 | 0,323 |
| **EContig181_2** | 667 | 2,000 | 1,993 | 0,691 | 0,460 | 0,498 | 0,33308 | 0,374 |
| **EContig1813_3** | 668 | 2,000 | 1,986 | 0,690 | 0,670 | 0,496 | 0,43713 | 0,373 |
| **EContig1816_1** | 667 | 2,000 | 1,971 | 0,686 | 0,654 | 0,493 | 0,36036 | 0,371 |
| **EContig1839** | 667 | 2,000 | 1,961 | 0,683 | 0,554 | 0,490 | 0,48952 | 0,370 |
| **EContig188** | 667 | 2,000 | 1,606 | 0,565 | 0,436 | 0,377 | 0,34656 | 0,306 |
| **EContig1947_3** | 668 | 2,000 | 1,969 | 0,685 | 0,543 | 0,492 | 0,38275 | 0,371 |
| **EContig1962_1** | 668 | 2,000 | 1,880 | 0,661 | 0,565 | 0,468 | 0,38235 | 0,359 |
| **EContig1987_2** | 668 | 2,000 | 1,808 | 0,639 | 0,423 | 0,447 | 0,32485 | 0,347 |
| **EContig2068_2** | 668 | 2,000 | 2,000 | 0,693 | 0,465 | 0,500 | 0,49925 | 0,375 |
| **EContig2172_2** | 667 | 2,000 | 1,744 | 0,618 | 0,485 | 0,427 | 0,19611 | 0,336 |
| **EContig2305_1** | 666 | 2,000 | 1,955 | 0,682 | 0,483 | 0,489 | 0,45045 | 0,369 |
| **EContig2458_2** | 650 | 2,000 | 1,864 | 0,656 | 0,462 | 0,463 | 0,43713 | 0,356 |
| **EContig2514** | 668 | 2,000 | 1,967 | 0,685 | 0,507 | 0,492 | 0,38253 | 0,371 |
| **EContig2705_2** | 664 | 2,000 | 1,920 | 0,672 | 0,492 | 0,479 | 0,33757 | 0,364 |
| **EContig2785_2** | 668 | 2,000 | 1,998 | 0,693 | 0,652 | 0,500 | 0,47081 | 0,375 |
| **EContig2906_2** | 668 | 2,000 | 1,819 | 0,643 | 0,493 | 0,450 | 0,30689 | 0,349 |
| **EContig2937_4** | 667 | 2,000 | 1,401 | 0,460 | 0,295 | 0,286 | 0,35263 | 0,245 |
| **EContig2950_2** | 661 | 2,000 | 1,598 | 0,562 | 0,329 | 0,374 | 0,39752 | 0,304 |
| **EContig298_1** | 668 | 2,000 | 1,996 | 0,692 | 0,504 | 0,499 | 0,46632 | 0,374 |
| **EContig2998** | 668 | 2,000 | 1,999 | 0,693 | 0,698 | 0,500 | 0,33784 | 0,375 |
| **EContig3075** | 667 | 2,000 | 1,756 | 0,622 | 0,537 | 0,431 | 0,48503 | 0,338 |
| **EContig3144_2** | 666 | 2,000 | 1,856 | 0,654 | 0,373 | 0,461 | 0,25655 | 0,355 |
| **EContig3225_1** | 667 | 2,000 | 1,983 | 0,689 | 0,573 | 0,496 | 0,4512 | 0,373 |
| **EContig3597_1** | 668 | 2,000 | 1,462 | 0,496 | 0,327 | 0,316 | 0,32175 | 0,266 |
| **EContig3889** | 668 | 2,000 | 1,993 | 0,691 | 0,519 | 0,498 | 0,28097 | 0,374 |
| **EContig3935_2** | 668 | 2,000 | 1,958 | 0,682 | 0,480 | 0,489 | 0,30885 | 0,370 |
| **EContig4112** | 668 | 2,000 | 2,000 | 0,693 | 0,525 | 0,500 | 0,42665 | 0,375 |
| **EContig4297_2** | 668 | 2,000 | 1,881 | 0,661 | 0,414 | 0,468 | 0,39955 | 0,359 |
| **EContig435_10** | 664 | 2,000 | 1,814 | 0,641 | 0,493 | 0,449 | 0,23802 | 0,348 |
| **EContig4380_1** | 611 | 2,000 | 1,997 | 0,692 | 0,649 | 0,499 | 0,39446 | 0,375 |
| **EContig4424_1** | 668 | 2,000 | 1,769 | 0,626 | 0,477 | 0,435 | 0,40344 | 0,340 |
| **EContig4490** | 649 | 2,000 | 1,618 | 0,570 | 0,388 | 0,382 | 0,31667 | 0,309 |
| **EContig460_6** | 651 | 2,000 | 1,953 | 0,681 | 0,513 | 0,488 | 0,34281 | 0,369 |

| Locus | N | $N_{avg}$ | $N_e$ | *I* | $H_o$ | $H_e$ | *MAF* | *PIC*** |
|---|---|---|---|---|---|---|---|---|
| **EContig465** | 668 | 2,000 | 1,999 | 0,693 | 0,555 | 0,500 | 0,42243 | 0,375 |
| **EContig4794** | 668 | 2,000 | 1,741 | 0,617 | 0,432 | 0,426 | 0,44378 | 0,335 |
| **EContig504_6** | 667 | 2,000 | 1,923 | 0,673 | 0,554 | 0,480 | 0,38772 | 0,365 |
| **EContig5140** | 663 | 2,000 | 1,831 | 0,646 | 0,566 | 0,454 | 0,47076 | 0,351 |
| **EContig515_2** | 666 | 2,000 | 1,953 | 0,681 | 0,600 | 0,488 | 0,42492 | 0,369 |
| **EContig515_3** | 668 | 2,000 | 1,614 | 0,568 | 0,425 | 0,381 | 0,49775 | 0,308 |
| **EContig5168_5** | 668 | 2,000 | 1,902 | 0,667 | 0,534 | 0,474 | 0,42342 | 0,362 |
| **EContig5183** | 667 | 2,000 | 1,780 | 0,630 | 0,406 | 0,438 | 0,37856 | 0,342 |
| **EContig5193** | 668 | 2,000 | 1,512 | 0,522 | 0,375 | 0,339 | 0,33458 | 0,281 |
| **EContig5258** | 655 | 2,000 | 1,960 | 0,683 | 0,476 | 0,490 | 0,36069 | 0,370 |
| **EContig526_2** | 668 | 2,000 | 1,789 | 0,633 | 0,498 | 0,441 | 0,48126 | 0,344 |
| **EContig5399_1** | 667 | 2,000 | 1,918 | 0,672 | 0,635 | 0,479 | 0,17241 | 0,364 |
| **EContig5478_1** | 668 | 2,000 | 1,897 | 0,666 | 0,453 | 0,473 | 0,34842 | 0,361 |
| **EContig5674_1** | 668 | 2,000 | 1,828 | 0,645 | 0,532 | 0,453 | 0,37575 | 0,350 |
| **EContig6361** | 665 | 2,000 | 1,841 | 0,649 | 0,523 | 0,457 | 0,37725 | 0,352 |
| **EContig6479** | 668 | 2,000 | 1,570 | 0,549 | 0,390 | 0,363 | 0,45808 | 0,297 |
| **EContig6629** | 667 | 2,000 | 1,889 | 0,663 | 0,482 | 0,471 | 0,36462 | 0,360 |
| **EContig6851** | 668 | 2,000 | 1,693 | 0,599 | 0,472 | 0,409 | 0,37425 | 0,326 |
| **EContig695_2** | 666 | 2,000 | 1,980 | 0,688 | 0,468 | 0,495 | 0,32384 | 0,372 |
| **EContig698_3** | 666 | 2,000 | 1,981 | 0,688 | 0,495 | 0,495 | 0,28668 | 0,373 |
| **EContig6995** | 667 | 2,000 | 1,975 | 0,687 | 0,567 | 0,494 | 0,3203 | 0,372 |
| **EContig7442_2** | 665 | 2,000 | 1,995 | 0,692 | 0,532 | 0,499 | 0,34522 | 0,374 |
| **EContig749_1** | 664 | 2,000 | 2,000 | 0,693 | 0,549 | 0,500 | 0,30405 | 0,375 |
| **EContig8421_1** | 668 | 2,000 | 1,883 | 0,662 | 0,457 | 0,469 | 0,24962 | 0,359 |
| **EContig845_1** | 668 | 2,000 | 1,966 | 0,684 | 0,567 | 0,491 | 0,25599 | 0,371 |
| **EContig86** | 661 | 2,000 | 1,896 | 0,665 | 0,452 | 0,473 | 0,36751 | 0,361 |
| **EContig8662** | 668 | 2,000 | 1,869 | 0,658 | 0,553 | 0,465 | 0,42954 | 0,357 |
| **EContig894_1** | 644 | 2,000 | 1,921 | 0,672 | 0,285 | 0,479 | 0,44003 | 0,364 |
| **EContig8993_1** | 666 | 2,000 | 1,953 | 0,681 | 0,550 | 0,488 | 0,43563 | 0,369 |
| **EContig9136_3** | 668 | 2,000 | 1,915 | 0,671 | 0,484 | 0,478 | 0,33886 | 0,364 |
| **EContig9157_1** | 667 | 2,000 | 1,649 | 0,583 | 0,440 | 0,394 | 0,32784 | 0,316 |
| **EContig9302_3** | 668 | 2,000 | 1,803 | 0,638 | 0,428 | 0,445 | 0,47594 | 0,346 |
| **EContig9302_4** | 665 | 2,000 | 1,772 | 0,627 | 0,518 | 0,436 | 0,4274 | 0,341 |
| **EContig9677** | 668 | 2,000 | 1,995 | 0,692 | 0,487 | 0,499 | 0,47305 | 0,374 |
| **EContig9922_3** | 668 | 2,000 | 1,969 | 0,685 | 0,549 | 0,492 | 0,26276 | 0,371 |
| **IContig1226_3** | 668 | 2,000 | 1,958 | 0,682 | 0,492 | 0,489 | 0,48653 | 0,370 |
| **IContig125_1** | 662 | 2,000 | 1,775 | 0,628 | 0,448 | 0,437 | 0,38623 | 0,341 |
| **IContig1260_3** | 667 | 2,000 | 1,990 | 0,691 | 0,699 | 0,498 | 0,42192 | 0,374 |
| **IContig19_3** | 668 | 2,000 | 1,904 | 0,668 | 0,516 | 0,475 | 0,42665 | 0,362 |
| **IContig2083_2** | 668 | 2,000 | 1,887 | 0,663 | 0,456 | 0,470 | 0,42954 | 0,360 |
| **IContig2350_3** | 667 | 2,000 | 1,961 | 0,683 | 0,518 | 0,490 | 0,39759 | 0,370 |
| **IContig257_8** | 667 | 2,000 | 1,908 | 0,669 | 0,493 | 0,476 | 0,482 | 0,363 |
| **IContig2640_3** | 663 | 2,000 | 1,895 | 0,665 | 0,617 | 0,472 | 0,3973 | 0,361 |
| **IContig2753_2** | 668 | 2,000 | 1,957 | 0,682 | 0,610 | 0,489 | 0,49548 | 0,369 |
| **IContig2783_3** | 668 | 2,000 | 1,991 | 0,691 | 0,644 | 0,498 | 0,46477 | 0,374 |
| **IContig2981_4** | 668 | 2,000 | 1,928 | 0,674 | 0,478 | 0,481 | 0,40719 | 0,366 |
| **IContig3160_2** | 664 | 2,000 | 1,858 | 0,654 | 0,519 | 0,462 | 0,30135 | 0,355 |

| Locus | N | $N_{avg}$ | $N_e$ | $I$ | $H_o$ | $H_e$ | *MAF* | *PIC*** |
|---|---|---|---|---|---|---|---|---|
| **IContig353** | 659 | 2,000 | 1,826 | 0,645 | 0,485 | 0,452 | 0,31334 | 0,350 |
| **IContig3651** | 668 | 2,000 | 1,995 | 0,692 | 0,601 | 0,499 | 0,21557 | 0,374 |
| **IContig52_2** | 668 | 2,000 | 1,933 | 0,676 | 0,625 | 0,483 | 0,26987 | 0,366 |
| **IContig667_1** | 667 | 2,000 | 1,780 | 0,630 | 0,448 | 0,438 | 0,32459 | 0,342 |
| **MEAN** | **664,9** | **2,000** | **1,862** | **0,652** | **0,501** | **0,460** | **0,380** | **0,353** |

*N = Number of non-redundant genotypes ; $N_{avg}$ = Average number of observed alleles ; $N_e$ = Number of effective alleles ; I = Shannon's Information Index ; $H_o$ = Observed Heterozygosity ; $H_e$ = Expected Heterozygosity ; MAF = Minor Allele Frequency ; PIC = Polymorphic Information Content ;

**Calculated by CERVUS v.3.0 software following Marshall *et al*. (1998) [72], while the rest of the data were calculated by GenAlex 6.5 software following Peakall and Smouse (2012) [71].
