# Supplementary material for: Utility of EST-SNP Markers for Improving Management and Use of Olive Genetic Resources: A Case Study at the Worldwide Olive Germplasm Bank of Córdoba
Source: Plants (Basel). 2022 Mar 29;11(7):921. doi: 10.3390/plants11070921 (PMC9002360; doi:10.3390/plants11070921)
Supplement: Supplementary file 1 [file plants-11-00921-s001.zip › Table S6.pdf]

**Supplemental Table S6:** Analysis of molecular variance (AMOVA) for the partitioning of genetic variation in olive genotypes within and between Mediterranean regions (eastern/ central/ western)

| Source of variation | df | SS | MS | Est. Var. | % | $\varphi_{ST}$ | $P_{(\varphi)}$ |
|---|---|---|---|---|---|---|---|
| **Among Regions** | 2 | 1746.69 | 873,350 | 3883,000 | 9.08% | 0.091 | <0.0001 |
| **Within Regions** | 665 | 25880.81 | 38.86 | 38.86 | 90.92% | | |

*(df = degree of freedom, SS = sum of squares, MS = mean squares, Est. var. = estimate of variance, % = Percentage of total variation,* $\varphi_{ST}$ *= Phi statistic, P(*$\phi$*)* $\varphi_{ST}$ *probability level after 9999 permutations)*
