# Supplementary material for: Utility of EST-SNP Markers for Improving Management and Use of Olive Genetic Resources: A Case Study at the Worldwide Olive Germplasm Bank of Córdoba
Source: Plants (Basel). 2022 Mar 29;11(7):921. doi: 10.3390/plants11070921 (PMC9002360; doi:10.3390/plants11070921)
Supplement: Supplementary file 1 [file plants-11-00921-s001.zip › Table S7_revised.pdf]

Supplemental Table S7: Proportion of genome of the 668 different genotypes assigned to the three clusters (gene pools) defined with the model-based clustering method from Pritchard *et al* . (2000) [75].

Register Number	ORIGIN*	REGION	K=3**		
			A	B	C
848	CYP	EM	0,922	0,025	0,053
721	EGY	EM	0,535	0,018	0,448
1737	EGY	EM	0,603	0,022	0,376
1094	EGY	EM	0,019	0,346	0,634
1750	EGY	EM	0,245	0,471	0,284
1739	EGY	EM	0,648	0,024	0,328
1744	EGY	EM	0,562	0,029	0,409
1754	EGY	EM	0,199	0,360	0,441
1753	EGY	EM	0,315	0,314	0,371
724	EGY	EM	0,408	0,412	0,180
725	EGY	EM	0,390	0,460	0,150
1742	EGY	EM	0,814	0,112	0,075
1738	EGY	EM	0,676	0,021	0,303
1749	EGY	EM	0,063	0,031	0,906
1741	EGY	EM	0,733	0,104	0,164
1745	EGY	EM	0,468	0,436	0,096
1747	EGY	EM	0,927	0,050	0,023
1748	EGY	EM	0,702	0,028	0,270
1099	EGY	EM	0,271	0,421	0,309
2167	EGY	EM	0,824	0,097	0,079
2169	EGY	EM	0,273	0,358	0,369
1139	IRN	EM	0,899	0,060	0,041
1810	IRN	EM	0,806	0,068	0,126
1141	IRN	EM	0,827	0,088	0,085
1142	IRN	EM	0,915	0,033	0,053
1140	IRN	EM	0,886	0,081	0,033
1143	IRN	EM	0,953	0,024	0,023
1856	IRN	EM	0,838	0,093	0,069
1854	IRN	EM	0,867	0,075	0,059
1853	IRN	EM	0,677	0,034	0,290
2278	IRQ	EM	0,652	0,052	0,296
2279	IRQ	EM	0,781	0,050	0,169
2280	IRQ	EM	0,742	0,141	0,117
158	ISR	EM	0,076	0,051	0,873
1165	ISR	EM	0,027	0,185	0,788
1970	ISR	EM	0,366	0,024	0,611
1972	ISR	EM	0,336	0,041	0,624
1971	ISR	EM	0,593	0,076	0,332
711	ISR	EM	0,238	0,421	0,341
120	ISR	EM	0,496	0,042	0,462
1808	JOR	EM	0,913	0,034	0,053
2254	JOR	EM	0,826	0,029	0,145
2261	JOR	EM	0,512	0,417	0,071

Register Number	ORIGIN*	REGION	K=3**		
			A	B	C
133	LBN	EM	0,869	0,036	0,095
2069	LBN	EM	0,923	0,055	0,022
2072	LBN	EM	0,872	0,101	0,027
2074	LBN	EM	0,889	0,057	0,055
2082	LBN	EM	0,817	0,085	0,098
787	SYR	EM	0,890	0,027	0,083
1010	SYR	EM	0,916	0,032	0,052
142	SYR	EM	0,973	0,015	0,012
1059	SYR	EM	0,915	0,019	0,066
140	SYR	EM	0,852	0,025	0,123
1054	SYR	EM	0,967	0,011	0,022
1043	SYR	EM	0,932	0,033	0,035
1794	SYR	EM	0,969	0,011	0,020
846	SYR	EM	0,952	0,026	0,022
1004	SYR	EM	0,970	0,011	0,019
788	SYR	EM	0,956	0,012	0,032
1562	SYR	EM	0,744	0,049	0,206
1000	SYR	EM	0,898	0,025	0,077
1572	SYR	EM	0,915	0,019	0,066
1568	SYR	EM	0,887	0,057	0,056
1557	SYR	EM	0,830	0,068	0,102
1780	SYR	EM	0,734	0,118	0,148
1788	SYR	EM	0,930	0,048	0,022
1786	SYR	EM	0,874	0,052	0,074
1034	SYR	EM	0,971	0,016	0,013
1115	SYR	EM	0,898	0,021	0,080
1605	SYR	EM	0,897	0,029	0,074
2058	SYR	EM	0,940	0,019	0,041
152	SYR	EM	0,736	0,167	0,097
145	SYR	EM	0,841	0,025	0,134
840	SYR	EM	0,969	0,014	0,017
1041	SYR	EM	0,804	0,074	0,122
842	SYR	EM	0,966	0,017	0,017
841	SYR	EM	0,965	0,017	0,018
1056	SYR	EM	0,924	0,024	0,052
990	SYR	EM	0,915	0,035	0,050
992	SYR	EM	0,602	0,373	0,025
1044	SYR	EM	0,965	0,012	0,023
1026	SYR	EM	0,850	0,113	0,037
1029	SYR	EM	0,944	0,013	0,043
1119	SYR	EM	0,934	0,017	0,050
1121	SYR	EM	0,955	0,018	0,027
1120	SYR	EM	0,883	0,036	0,082
1116	SYR	EM	0,910	0,047	0,043
1125	SYR	EM	0,947	0,018	0,035
1118	SYR	EM	0,824	0,024	0,152

Register Number	ORIGIN*	REGION	K=3**		
			A	B	C
1122	SYR	EM	0,967	0,013	0,021
1124	SYR	EM	0,872	0,050	0,078
2501	SYR	EM	0,912	0,043	0,045
1002	SYR	EM	0,839	0,124	0,037
1559	SYR	EM	0,878	0,018	0,104
1561	SYR	EM	0,902	0,018	0,080
1563	SYR	EM	0,947	0,027	0,026
1564	SYR	EM	0,897	0,020	0,084
1595	SYR	EM	0,954	0,020	0,026
1599	SYR	EM	0,917	0,023	0,060
1600	SYR	EM	0,425	0,243	0,333
1602	SYR	EM	0,801	0,026	0,174
1606	SYR	EM	0,746	0,030	0,225
1607	SYR	EM	0,924	0,024	0,052
1609	SYR	EM	0,874	0,019	0,106
1613	SYR	EM	0,908	0,043	0,049
1615	SYR	EM	0,969	0,017	0,014
1616	SYR	EM	0,968	0,019	0,013
1617	SYR	EM	0,933	0,043	0,025
1618	SYR	EM	0,915	0,019	0,066
1623	SYR	EM	0,961	0,016	0,023
1624	SYR	EM	0,960	0,025	0,015
1628	SYR	EM	0,850	0,027	0,123
2057	SYR	EM	0,780	0,038	0,182
685	TUR	EM	0,870	0,093	0,037
97	TUR	EM	0,662	0,321	0,018
93	TUR	EM	0,880	0,021	0,099
690	TUR	EM	0,866	0,105	0,030
92	TUR	EM	0,594	0,017	0,389
686	TUR	EM	0,794	0,025	0,181
679	TUR	EM	0,527	0,039	0,433
688	TUR	EM	0,855	0,108	0,036
95	TUR	EM	0,355	0,024	0,621
99	TUR	EM	0,778	0,025	0,197
94	TUR	EM	0,804	0,164	0,032
2318	TUR	EM	0,678	0,090	0,232
2290	TUR	EM	0,587	0,032	0,381
2291	TUR	EM	0,655	0,025	0,320
2292	TUR	EM	0,608	0,020	0,372
2317	TUR	EM	0,728	0,054	0,218
2295	TUR	EM	0,453	0,023	0,524
2297	TUR	EM	0,617	0,027	0,356
2298	TUR	EM	0,412	0,040	0,548
2300	TUR	EM	0,487	0,017	0,496
2304	TUR	EM	0,531	0,029	0,440
2306	TUR	EM	0,751	0,019	0,230

Register Number	ORIGIN*	REGION	K=3**		
			A	B	C
2311	TUR	EM	0,529	0,016	0,455
2315	TUR	EM	0,666	0,044	0,290
2316	TUR	EM	0,502	0,013	0,485
2319	TUR	EM	0,900	0,051	0,049
2329	TUR	EM	0,862	0,023	0,115
2332	TUR	EM	0,581	0,245	0,174
2336	TUR	EM	0,962	0,018	0,020
2338	TUR	EM	0,936	0,019	0,045
2340	TUR	EM	0,892	0,021	0,087
2341	TUR	EM	0,900	0,025	0,075
2343	TUR	EM	0,942	0,034	0,024
2344	TUR	EM	0,947	0,024	0,030
2345	TUR	EM	0,836	0,061	0,103
2347	TUR	EM	0,658	0,230	0,113
2349	TUR	EM	0,882	0,041	0,077
2351	TUR	EM	0,630	0,027	0,343
2359	TUR	EM	0,940	0,036	0,024
2363	TUR	EM	0,767	0,211	0,023
2364	TUR	EM	0,526	0,063	0,411
2365	TUR	EM	0,083	0,027	0,890
1082	ALB	CM	0,809	0,177	0,014
1079	ALB	CM	0,839	0,141	0,020
1081	ALB	CM	0,031	0,567	0,403
1077	ALB	CM	0,762	0,222	0,017
2499	ALB	CM	0,057	0,883	0,060
1812	ALB	CM	0,175	0,773	0,052
1078	ALB	CM	0,430	0,358	0,213
1151	ALB	CM	0,592	0,378	0,030
1171	ALB	CM	0,771	0,153	0,076
1716	ALB	CM	0,879	0,103	0,019
1714	ALB	CM	0,074	0,333	0,593
1711	ALB	CM	0,512	0,471	0,018
2268	ALB	CM	0,337	0,614	0,049
2270	ALB	CM	0,458	0,501	0,041
1537	DZA	CM	0,724	0,070	0,206
1655	DZA	CM	0,030	0,948	0,022
1541	DZA	CM	0,045	0,636	0,319
1542	DZA	CM	0,659	0,079	0,261
1525	DZA	CM	0,601	0,015	0,384
1523	DZA	CM	0,037	0,490	0,473
1638	DZA	CM	0,544	0,414	0,042
1659	DZA	CM	0,045	0,479	0,477
1634	DZA	CM	0,067	0,342	0,591
1948	DZA	CM	0,292	0,382	0,326
1947	DZA	CM	0,116	0,832	0,052
1530	DZA	CM	0,516	0,040	0,443

Register Number	ORIGIN*	REGION	K=3**		
			A	B	C
1951	DZA	CM	0,141	0,710	0,149
118	DZA	CM	0,024	0,611	0,365
1513	DZA	CM	0,057	0,899	0,044
1539	DZA	CM	0,605	0,063	0,332
1543	DZA	CM	0,216	0,331	0,454
2500	DZA	CM	0,071	0,877	0,052
1518	DZA	CM	0,049	0,733	0,218
1548	DZA	CM	0,244	0,651	0,105
1549	DZA	CM	0,266	0,635	0,099
1516	DZA	CM	0,469	0,025	0,506
1534	DZA	CM	0,442	0,052	0,506
1529	DZA	CM	0,029	0,947	0,024
1524	DZA	CM	0,095	0,871	0,034
1547	DZA	CM	0,073	0,561	0,366
1850	DZA	CM	0,041	0,919	0,040
1849	DZA	CM	0,019	0,955	0,026
695	GRC	CM	0,620	0,037	0,344
219	GRC	CM	0,670	0,025	0,306
692	GRC	CM	0,934	0,048	0,018
103	GRC	CM	0,806	0,090	0,104
228	GRC	CM	0,630	0,093	0,277
699	GRC	CM	0,169	0,756	0,075
1385	GRC	CM	0,877	0,109	0,015
2227	GRC	CM	0,280	0,692	0,028
1394	GRC	CM	0,264	0,589	0,147
1392	GRC	CM	0,071	0,684	0,245
218	GRC	CM	0,062	0,899	0,039
1681	GRC	CM	0,209	0,284	0,507
345	GRC	CM	0,296	0,685	0,019
694	GRC	CM	0,016	0,966	0,018
105	GRC	CM	0,302	0,120	0,578
702	GRC	CM	0,709	0,157	0,135
691	GRC	CM	0,052	0,259	0,689
220	GRC	CM	0,611	0,036	0,354
2206	GRC	CM	0,683	0,234	0,083
2207	GRC	CM	0,512	0,038	0,451
2208	GRC	CM	0,895	0,037	0,067
2209	GRC	CM	0,271	0,591	0,138
2212	GRC	CM	0,382	0,499	0,119
2234	GRC	CM	0,712	0,041	0,248
2236	GRC	CM	0,656	0,020	0,324
2237	GRC	CM	0,080	0,883	0,037
2238	GRC	CM	0,657	0,014	0,330
2239	GRC	CM	0,699	0,035	0,266
1990	HRV	CM	0,407	0,497	0,096
1982	HRV	CM	0,094	0,480	0,427

Register Number	ORIGIN*	REGION	K=3**		
			A	B	C
1991	HRV	CM	0,054	0,703	0,243
1974	HRV	CM	0,475	0,064	0,461
1987	HRV	CM	0,289	0,053	0,658
1985	HRV	CM	0,362	0,598	0,040
733	HRV	CM	0,304	0,051	0,645
1977	HRV	CM	0,304	0,598	0,098
1979	HRV	CM	0,125	0,110	0,765
1981	HRV	CM	0,020	0,447	0,533
1986	HRV	CM	0,280	0,038	0,682
1978	HRV	CM	0,255	0,063	0,682
2005	HRV	CM	0,082	0,580	0,337
703	HRV	CM	0,048	0,363	0,590
704	HRV	CM	0,344	0,608	0,048
706	HRV	CM	0,290	0,161	0,548
734	HRV	CM	0,155	0,464	0,381
2387	HRV	CM	0,179	0,045	0,776
2390	HRV	CM	0,276	0,366	0,358
2388	HRV	CM	0,046	0,569	0,385
1349	ITA	CM	0,584	0,035	0,381
1227	ITA	CM	0,266	0,575	0,159
1269	ITA	CM	0,094	0,537	0,369
85	ITA	CM	0,256	0,670	0,074
1254	ITA	CM	0,408	0,531	0,061
87	ITA	CM	0,075	0,564	0,361
80	ITA	CM	0,066	0,901	0,033
1268	ITA	CM	0,522	0,024	0,454
68	ITA	CM	0,430	0,055	0,515
1348	ITA	CM	0,222	0,703	0,075
1189	ITA	CM	0,197	0,579	0,224
1236	ITA	CM	0,251	0,698	0,051
1194	ITA	CM	0,658	0,308	0,034
88	ITA	CM	0,192	0,726	0,082
1210	ITA	CM	0,273	0,673	0,054
82	ITA	CM	0,129	0,590	0,281
84	ITA	CM	0,098	0,667	0,236
1253	ITA	CM	0,084	0,110	0,806
1170	ITA	CM	0,351	0,589	0,061
1239	ITA	CM	0,086	0,345	0,570
1247	ITA	CM	0,420	0,268	0,312
75	ITA	CM	0,580	0,019	0,401
1241	ITA	CM	0,196	0,405	0,399
1323	ITA	CM	0,308	0,054	0,638
1352	ITA	CM	0,497	0,021	0,482
1280	ITA	CM	0,304	0,071	0,625
62	ITA	CM	0,373	0,033	0,594
1304	ITA	CM	0,022	0,445	0,533

Register Number	ORIGIN*	REGION	K=3**		
			A	B	C
1341	ITA	CM	0,442	0,138	0,421
1267	ITA	CM	0,624	0,142	0,234
736	ITA	CM	0,574	0,019	0,407
79	ITA	CM	0,042	0,524	0,434
1324	ITA	CM	0,492	0,045	0,464
1340	ITA	CM	0,346	0,042	0,612
1277	ITA	CM	0,020	0,032	0,948
1255	ITA	CM	0,279	0,279	0,443
1332	ITA	CM	0,061	0,390	0,549
1337	ITA	CM	0,537	0,054	0,409
1211	ITA	CM	0,208	0,619	0,172
1329	ITA	CM	0,102	0,075	0,823
1350	ITA	CM	0,490	0,456	0,053
1334	ITA	CM	0,135	0,403	0,462
1193	ITA	CM	0,231	0,440	0,329
1250	ITA	CM	0,132	0,673	0,195
90	ITA	CM	0,562	0,300	0,138
1271	ITA	CM	0,398	0,582	0,021
1331	ITA	CM	0,280	0,457	0,263
1327	ITA	CM	0,006	0,982	0,012
77	ITA	CM	0,022	0,938	0,040
1325	ITA	CM	0,910	0,059	0,031
1328	ITA	CM	0,661	0,017	0,322
1207	ITA	CM	0,126	0,349	0,525
1219	ITA	CM	0,161	0,553	0,287
1222	ITA	CM	0,025	0,877	0,098
1187	ITA	CM	0,248	0,503	0,249
1224	ITA	CM	0,254	0,071	0,675
1226	ITA	CM	0,047	0,513	0,440
1223	ITA	CM	0,081	0,865	0,054
1188	ITA	CM	0,198	0,696	0,106
1342	ITA	CM	0,527	0,027	0,446
1203	ITA	CM	0,125	0,589	0,286
1230	ITA	CM	0,139	0,668	0,193
1221	ITA	CM	0,039	0,871	0,091
1216	ITA	CM	0,093	0,761	0,147
1215	ITA	CM	0,042	0,562	0,396
1212	ITA	CM	0,381	0,566	0,053
1225	ITA	CM	0,038	0,434	0,529
1195	ITA	CM	0,534	0,381	0,085
1353	ITA	CM	0,132	0,464	0,405
1322	ITA	CM	0,548	0,038	0,413
1201	ITA	CM	0,104	0,529	0,368
2018	ITA	CM	0,012	0,944	0,044
65	ITA	CM	0,352	0,610	0,038
893	ITA	CM	0,048	0,818	0,134

Register Number	ORIGIN*	REGION	K=3**		
			A	B	C
1270	ITA	CM	0,066	0,858	0,076
2496	ITA	CM	0,096	0,490	0,414
1994	MNE	CM	0,374	0,477	0,149
1995	MNE	CM	0,811	0,166	0,023
1997	MNE	CM	0,302	0,646	0,052
1999	MNE	CM	0,639	0,341	0,020
2273	SVN	CM	0,440	0,144	0,416
115	TUN	CM	0,898	0,017	0,085
113	TUN	CM	0,274	0,239	0,487
538	TUN	CM	0,926	0,028	0,046
1674	TUN	CM	0,636	0,334	0,030
1872	TUN	CM	0,302	0,558	0,141
1725	TUN	CM	0,623	0,043	0,334
1509	TUN	CM	0,542	0,109	0,349
1683	TUN	CM	0,547	0,432	0,022
1661	TUN	CM	0,161	0,028	0,811
1684	TUN	CM	0,187	0,501	0,312
1832	TUN	CM	0,685	0,275	0,040
1871	TUN	CM	0,034	0,937	0,029
1917	TUN	CM	0,579	0,067	0,355
1680	TUN	CM	0,227	0,555	0,218
1893	TUN	CM	0,023	0,950	0,027
1873	TUN	CM	0,299	0,558	0,143
1898	TUN	CM	0,023	0,957	0,020
1908	TUN	CM	0,711	0,256	0,034
1670	TUN	CM	0,104	0,269	0,626
1887	TUN	CM	0,015	0,959	0,026
1864	TUN	CM	0,055	0,537	0,408
1511	TUN	CM	0,053	0,804	0,143
1888	TUN	CM	0,023	0,939	0,038
1890	TUN	CM	0,016	0,963	0,021
1912	TUN	CM	0,019	0,950	0,031
1869	TUN	CM	0,018	0,961	0,021
1889	TUN	CM	0,658	0,244	0,099
1914	TUN	CM	0,072	0,540	0,388
1876	TUN	CM	0,012	0,973	0,015
1910	TUN	CM	0,037	0,941	0,022
1916	TUN	CM	0,154	0,738	0,108
1728	TUN	CM	0,046	0,259	0,695
1732	TUN	CM	0,017	0,967	0,016
114	TUN	CM	0,236	0,743	0,021
1902	TUN	CM	0,069	0,825	0,106
1825	TUN	CM	0,023	0,962	0,015
1675	TUN	CM	0,774	0,115	0,111
539	TUN	CM	0,008	0,977	0,015
1918	TUN	CM	0,051	0,557	0,393

Register Number	ORIGIN*	REGION	K=3**		
			A	B	C
1907	TUN	CM	0,253	0,716	0,032
1899	TUN	CM	0,017	0,967	0,016
1827	TUN	CM	0,022	0,963	0,015
1814	ARG	WM	0,458	0,021	0,521
829	ARG	WM	0,087	0,050	0,863
726	CHL	WM	0,028	0,025	0,947
1451	CHL	WM	0,038	0,361	0,601
1459	CHL	WM	0,033	0,939	0,028
1457	CHL	WM	0,040	0,075	0,885
1455	CHL	WM	0,127	0,287	0,586
9	ESP	WM	0,073	0,080	0,847
54	ESP	WM	0,052	0,508	0,440
5	ESP	WM	0,029	0,356	0,616
753	ESP	WM	0,031	0,073	0,895
877	ESP	WM	0,026	0,071	0,903
39	ESP	WM	0,126	0,023	0,851
234	ESP	WM	0,056	0,008	0,936
562	ESP	WM	0,066	0,502	0,432
770	ESP	WM	0,068	0,025	0,907
414	ESP	WM	0,035	0,059	0,907
35	ESP	WM	0,057	0,063	0,880
384	ESP	WM	0,042	0,060	0,899
44	ESP	WM	0,080	0,063	0,857
2	ESP	WM	0,021	0,033	0,946
27	ESP	WM	0,142	0,085	0,773
781	ESP	WM	0,136	0,267	0,597
394	ESP	WM	0,053	0,057	0,890
60	ESP	WM	0,103	0,066	0,832
756	ESP	WM	0,041	0,030	0,929
1373	ESP	WM	0,231	0,058	0,711
46	ESP	WM	0,932	0,033	0,035
749	ESP	WM	0,324	0,037	0,640
784	ESP	WM	0,065	0,057	0,879
779	ESP	WM	0,032	0,095	0,873
406	ESP	WM	0,042	0,082	0,876
51	ESP	WM	0,263	0,024	0,714
31	ESP	WM	0,023	0,091	0,886
309	ESP	WM	0,071	0,137	0,792
282	ESP	WM	0,054	0,026	0,920
669	ESP	WM	0,270	0,638	0,092
21	ESP	WM	0,051	0,028	0,921
1717	ESP	WM	0,310	0,590	0,100
1365	ESP	WM	0,064	0,081	0,856
1372	ESP	WM	0,127	0,042	0,831
970	ESP	WM	0,507	0,050	0,443
10	ESP	WM	0,057	0,042	0,901

Register Number	ORIGIN*	REGION	K=3**		
			A	B	C
1369	ESP	WM	0,483	0,105	0,412
965	ESP	WM	0,373	0,124	0,502
607	ESP	WM	0,074	0,028	0,899
1481	ESP	WM	0,060	0,017	0,923
224	ESP	WM	0,542	0,382	0,076
567	ESP	WM	0,054	0,155	0,791
768	ESP	WM	0,057	0,114	0,829
817	ESP	WM	0,167	0,665	0,169
1182	ESP	WM	0,284	0,076	0,640
26	ESP	WM	0,081	0,030	0,889
11	ESP	WM	0,360	0,616	0,024
227	ESP	WM	0,065	0,100	0,835
15	ESP	WM	0,185	0,261	0,555
757	ESP	WM	0,057	0,638	0,305
370	ESP	WM	0,112	0,044	0,844
41	ESP	WM	0,292	0,583	0,125
6	ESP	WM	0,042	0,070	0,888
306	ESP	WM	0,038	0,079	0,884
231	ESP	WM	0,256	0,723	0,021
1923	ESP	WM	0,336	0,417	0,248
790	ESP	WM	0,047	0,038	0,915
1818	ESP	WM	0,063	0,034	0,903
665	ESP	WM	0,350	0,463	0,187
256	ESP	WM	0,055	0,064	0,881
489	ESP	WM	0,052	0,062	0,885
255	ESP	WM	0,027	0,033	0,940
2022	ESP	WM	0,393	0,076	0,531
12	ESP	WM	0,350	0,570	0,080
2020	ESP	WM	0,544	0,146	0,310
2030	ESP	WM	0,511	0,459	0,030
2017	ESP	WM	0,179	0,167	0,653
2021	ESP	WM	0,522	0,426	0,052
2024	ESP	WM	0,645	0,034	0,321
16	ESP	WM	0,473	0,046	0,482
758	ESP	WM	0,046	0,069	0,884
645	ESP	WM	0,281	0,253	0,466
390	ESP	WM	0,259	0,032	0,709
226	ESP	WM	0,351	0,590	0,059
55	ESP	WM	0,070	0,049	0,881
2027	ESP	WM	0,668	0,078	0,254
2026	ESP	WM	0,486	0,273	0,241
1973	ESP	WM	0,487	0,467	0,046
2044	ESP	WM	0,613	0,180	0,207
2041	ESP	WM	0,529	0,035	0,436
2035	ESP	WM	0,586	0,157	0,256
366	ESP	WM	0,073	0,142	0,784

Register Number	ORIGIN*	REGION	K=3**		
			A	B	C
285	ESP	WM	0,050	0,078	0,873
2034	ESP	WM	0,494	0,049	0,457
2029	ESP	WM	0,943	0,043	0,014
2032	ESP	WM	0,668	0,051	0,281
2248	ESP	WM	0,318	0,330	0,352
2040	ESP	WM	0,388	0,078	0,534
2036	ESP	WM	0,430	0,055	0,515
663	ESP	WM	0,483	0,104	0,413
674	ESP	WM	0,219	0,496	0,285
333	ESP	WM	0,027	0,061	0,912
286	ESP	WM	0,070	0,042	0,889
3	ESP	WM	0,062	0,124	0,813
47	ESP	WM	0,730	0,254	0,016
23	ESP	WM	0,034	0,093	0,872
652	ESP	WM	0,104	0,311	0,585
593	ESP	WM	0,035	0,053	0,912
270	ESP	WM	0,023	0,131	0,846
297	ESP	WM	0,061	0,037	0,902
664	ESP	WM	0,113	0,571	0,316
677	ESP	WM	0,284	0,636	0,079
534	ESP	WM	0,060	0,038	0,903
371	ESP	WM	0,028	0,031	0,941
605	ESP	WM	0,062	0,056	0,883
123	ESP	WM	0,086	0,042	0,872
591	ESP	WM	0,164	0,040	0,796
750	ESP	WM	0,037	0,060	0,903
367	ESP	WM	0,464	0,097	0,440
305	ESP	WM	0,036	0,053	0,911
667	ESP	WM	0,087	0,812	0,101
653	ESP	WM	0,076	0,386	0,538
576	ESP	WM	0,040	0,047	0,913
666	ESP	WM	0,147	0,687	0,166
410	ESP	WM	0,083	0,025	0,892
269	ESP	WM	0,076	0,022	0,902
295	ESP	WM	0,035	0,085	0,880
17	ESP	WM	0,082	0,075	0,844
661	ESP	WM	0,262	0,591	0,147
613	ESP	WM	0,280	0,580	0,140
440	ESP	WM	0,123	0,023	0,854
20	ESP	WM	0,513	0,026	0,461
34	ESP	WM	0,162	0,052	0,787
273	ESP	WM	0,025	0,040	0,935
358	ESP	WM	0,028	0,143	0,829
437	ESP	WM	0,054	0,058	0,888
561	ESP	WM	0,080	0,107	0,813
418	ESP	WM	0,034	0,025	0,941

Register Number	ORIGIN*	REGION	K=3**		
			A	B	C
403	ESP	WM	0,120	0,031	0,849
372	ESP	WM	0,045	0,047	0,908
335	ESP	WM	0,044	0,057	0,899
402	ESP	WM	0,149	0,112	0,739
448	ESP	WM	0,135	0,051	0,814
349	ESP	WM	0,072	0,067	0,861
773	ESP	WM	0,036	0,032	0,932
565	ESP	WM	0,039	0,046	0,915
49	ESP	WM	0,051	0,035	0,914
364	ESP	WM	0,402	0,062	0,536
272	ESP	WM	0,031	0,029	0,940
392	ESP	WM	0,247	0,041	0,712
28	ESP	WM	0,057	0,039	0,904
408	ESP	WM	0,019	0,041	0,940
411	ESP	WM	0,099	0,054	0,847
29	ESP	WM	0,104	0,030	0,866
293	ESP	WM	0,091	0,066	0,843
649	ESP	WM	0,091	0,369	0,540
343	ESP	WM	0,042	0,024	0,935
361	ESP	WM	0,064	0,033	0,903
785	ESP	WM	0,038	0,062	0,901
277	ESP	WM	0,022	0,211	0,767
13	ESP	WM	0,452	0,502	0,046
332	ESP	WM	0,069	0,059	0,872
522	ESP	WM	0,025	0,215	0,760
387	ESP	WM	0,099	0,105	0,796
413	ESP	WM	0,040	0,050	0,911
427	ESP	WM	0,100	0,079	0,821
401	ESP	WM	0,065	0,044	0,891
348	ESP	WM	0,048	0,032	0,921
798	ESP	WM	0,117	0,049	0,834
778	ESP	WM	0,026	0,092	0,882
801	ESP	WM	0,182	0,070	0,748
284	ESP	WM	0,022	0,042	0,936
740	ESP	WM	0,499	0,036	0,465
802	ESP	WM	0,038	0,098	0,864
800	ESP	WM	0,077	0,587	0,336
883	ESP	WM	0,388	0,538	0,074
871	ESP	WM	0,663	0,261	0,077
360	ESP	WM	0,059	0,088	0,853
276	ESP	WM	0,035	0,027	0,938
957	ESP	WM	0,037	0,061	0,902
864	ESP	WM	0,082	0,093	0,826
774	ESP	WM	0,034	0,039	0,926
994	ESP	WM	0,570	0,062	0,369
355	ESP	WM	0,045	0,036	0,919

Register Number	ORIGIN*	REGION	K=3**		
			A	B	C
822	ESP	WM	0,435	0,418	0,147
1065	ESP	WM	0,175	0,471	0,354
660	ESP	WM	0,370	0,588	0,042
1069	ESP	WM	0,554	0,379	0,068
962	ESP	WM	0,747	0,228	0,025
1073	ESP	WM	0,525	0,420	0,056
966	ESP	WM	0,923	0,064	0,013
1068	ESP	WM	0,080	0,185	0,735
1072	ESP	WM	0,055	0,387	0,558
795	ESP	WM	0,074	0,064	0,862
969	ESP	WM	0,303	0,590	0,107
631	ESP	WM	0,392	0,036	0,573
365	ESP	WM	0,032	0,050	0,918
956	ESP	WM	0,020	0,114	0,866
797	ESP	WM	0,092	0,048	0,860
1448	ESP	WM	0,130	0,366	0,504
1720	ESP	WM	0,049	0,031	0,920
1440	ESP	WM	0,146	0,352	0,502
1445	ESP	WM	0,032	0,857	0,111
1442	ESP	WM	0,084	0,350	0,566
30	ESP	WM	0,055	0,054	0,892
1920	ESP	WM	0,432	0,340	0,228
771	ESP	WM	0,136	0,039	0,825
2253	ESP	WM	0,638	0,117	0,245
2042	ESP	WM	0,740	0,177	0,083
2100	ESP	WM	0,081	0,119	0,800
2134	ESP	WM	0,064	0,130	0,806
2136	ESP	WM	0,124	0,053	0,823
2144	ESP	WM	0,197	0,238	0,565
2148	ESP	WM	0,050	0,037	0,913
2151	ESP	WM	0,564	0,046	0,390
2153	ESP	WM	0,402	0,099	0,499
2155	ESP	WM	0,131	0,170	0,699
2156	ESP	WM	0,532	0,154	0,315
2157	ESP	WM	0,680	0,147	0,173
2160	ESP	WM	0,026	0,629	0,345
2162	ESP	WM	0,026	0,072	0,902
2165	ESP	WM	0,099	0,019	0,882
2171	ESP	WM	0,165	0,551	0,284
2173	ESP	WM	0,299	0,504	0,197
2175	ESP	WM	0,179	0,132	0,689
2176	ESP	WM	0,527	0,033	0,440
2177	ESP	WM	0,154	0,053	0,794
2179	ESP	WM	0,272	0,470	0,258
2180	ESP	WM	0,126	0,519	0,355
2181	ESP	WM	0,126	0,615	0,260

Register Number	ORIGIN*	REGION	K=3**		
			A	B	C
2182	ESP	WM	0,227	0,565	0,208
2183	ESP	WM	0,259	0,484	0,257
2184	ESP	WM	0,408	0,330	0,262
2185	ESP	WM	0,468	0,231	0,301
2186	ESP	WM	0,065	0,503	0,433
2187	ESP	WM	0,499	0,043	0,458
2188	ESP	WM	0,244	0,189	0,567
2189	ESP	WM	0,083	0,606	0,311
2196	ESP	WM	0,114	0,056	0,830
2192	ESP	WM	0,047	0,594	0,359
2193	ESP	WM	0,168	0,351	0,482
2194	ESP	WM	0,623	0,274	0,103
2197	ESP	WM	0,297	0,299	0,404
2198	ESP	WM	0,105	0,511	0,384
2199	ESP	WM	0,093	0,118	0,789
2201	ESP	WM	0,032	0,044	0,925
2242	ESP	WM	0,222	0,051	0,727
2244	ESP	WM	0,779	0,207	0,014
2246	ESP	WM	0,309	0,445	0,246
2247	ESP	WM	0,379	0,368	0,253
2249	ESP	WM	0,762	0,051	0,187
2250	ESP	WM	0,387	0,440	0,172
2251	ESP	WM	0,396	0,107	0,498
2252	ESP	WM	0,473	0,023	0,504
2276	ESP	WM	0,047	0,056	0,897
2282	ESP	WM	0,138	0,068	0,794
2283	ESP	WM	0,496	0,180	0,324
2132	ESP	WM	0,087	0,039	0,874
2394	ESP	WM	0,021	0,036	0,943
420	ESP	WM	0,194	0,044	0,762
322	FRA	WM	0,480	0,108	0,412
70	FRA	WM	0,251	0,162	0,587
63	FRA	WM	0,555	0,021	0,424
76	FRA	WM	0,188	0,100	0,712
73	FRA	WM	0,822	0,078	0,100
67	FRA	WM	0,555	0,056	0,390
708	FRA	WM	0,100	0,404	0,496
1848	FRA	WM	0,186	0,428	0,385
1091	FRA	WM	0,425	0,309	0,265
1845	FRA	WM	0,685	0,069	0,246
1846	FRA	WM	0,788	0,050	0,162
2204	FRA	WM	0,227	0,475	0,299
2210	FRA	WM	0,045	0,809	0,146
2214	FRA	WM	0,440	0,157	0,403
2215	FRA	WM	0,147	0,745	0,109
2216	FRA	WM	0,291	0,242	0,467

Register Number	ORIGIN*	REGION	K=3**		
			A	B	C
2218	FRA	WM	0,847	0,068	0,085
2219	FRA	WM	0,122	0,577	0,301
2220	FRA	WM	0,342	0,527	0,131
2222	FRA	WM	0,525	0,152	0,323
2224	FRA	WM	0,054	0,281	0,665
2228	FRA	WM	0,268	0,091	0,641
2229	FRA	WM	0,565	0,355	0,080
2230	FRA	WM	0,143	0,266	0,591
2231	FRA	WM	0,347	0,309	0,343
2232	FRA	WM	0,245	0,086	0,669
101	MAR	WM	0,024	0,027	0,949
1932	MAR	WM	0,013	0,630	0,357
1930	MAR	WM	0,141	0,822	0,038
1944	MAR	WM	0,092	0,063	0,845
1927	MAR	WM	0,015	0,513	0,472
1931	MAR	WM	0,012	0,651	0,337
1937	MAR	WM	0,087	0,065	0,848
1414	MEX	WM	0,194	0,160	0,647
1130	MEX	WM	0,337	0,064	0,599
1134	MEX	WM	0,035	0,377	0,588
2047	PER	WM	0,440	0,206	0,354
124	PRT	WM	0,107	0,036	0,857
125	PRT	WM	0,033	0,050	0,917
130	PRT	WM	0,038	0,050	0,912
128	PRT	WM	0,020	0,208	0,772
887	PRT	WM	0,078	0,377	0,545
707	USA	WM	0,093	0,056	0,851
	**Mean**	**EM**	**0,751**	**0,077**	**0,172**
		**CM**	**0,299**	**0,448**	**0,253**
		**WM**	**0,214**	**0,197**	**0,588**
		**Total**	**0,299**	**0,448**	**0,253**

*For same genotypes identified in different countries, a representative registration code and country of origin were chosen according to WOGBC historical information

** Lowest value — Percentile 50 — Highest value
